# Substituent effects on aromatic interactions in water†

**DOI:** 10.1039/d3sc01027a

**Published:** 2023-05-24

**Authors:** Gloria Tobajas-Curiel, Qingqing Sun, Jeremy K. M. Sanders, Pablo Ballester, Christopher A. Hunter

**Affiliations:** a Yusuf Hamied Department of Chemistry, University of Cambridge Cambridge CB2 1EW UK herchelsmith.orgchem@ch.cam.ac.uk; b Institute of Chemical Research of Catalonia (ICIQ), Barcelona Institute of Science and Technology (BIST) Av. Països Catalans, 16, 43007 Tarragona Spain pballester@iciq.es; c Yangzhou University, School of Chemistry and Chemical Engineering Yangzhou 225002 Jiangsu China sunqingqing@snnu.edu.cn; d ICREA Passeig Lluís Companys 23 08010 Barcelona Spain

## Abstract

Molecular recognition in water involves contributions due to polar functional group interactions, partial desolvation of polar and non-polar surfaces and changes in conformational flexibility, presenting a challenge for rational design and interpretation of supramolecular behaviour. Conformationally well-defined supramolecular complexes that can be studied in both water and non-polar solvents provide a platform for disentangling these contributions. Here 1 : 1 complexes formed between four different calix[4]pyrrole receptors and thirteen different pyridine *N*-oxide guests have been used to dissect the factors that govern substituent effects on aromatic interactions in water. H-bonding interactions between the receptor pyrrole donors and the guest *N*-oxide acceptor at one end of the complex lock the geometrical arrangement of a cluster of aromatic interactions at the other end of the complex, so that a phenyl group on the guest makes two edge-to-face and two stacking interactions with the four aromatic side-walls of the receptor. The thermodynamic contribution of these aromatic interactions to the overall stability of the complex was quantified by chemical double mutant cycles using isothermal titration calorimetry and ^1^H NMR competition experiments. Aromatic interactions between the receptor and a phenyl group on the guest stabilise the complex by a factor of 1000, and addition of substituents to the guest phenyl group further stabilises the complex by an additional factor of up to 1000. When a nitro substituent is present on the guest phenyl group, the complex has a sub-picomolar dissociation constant (370 fM). The remarkable substituent effects observed in water for these complexes can be rationalised by comparison with the magnitude of the corresponding substituent effects measured in chloroform. In chloroform, the double mutant cycle free energy measurements of the aromatic interactions correlate well with the substituent Hammett parameters. Electron-withdrawing substituents increase the strength of the interactions by a factor of up to 20, highlighting the role of electrostatics in stabilising both the edge-to-face and stacking interactions. The enhanced substituent effects observed in water are due to entropic contributions associated with the desolvation of hydrophobic surfaces on the substituents. The flexible alkyl chains that line the open end of the binding site assist the desolvation of the non-polar π-surfaces of polar substituents, like nitro, but at the same time allow water to interact with the polar H-bond acceptor sites on the substituent. This flexibility allows polar substituents to maximise non-polar interactions with the receptor and polar interactions with the solvent, leading to remarkably high binding affinities.

## Abstract 2

Measurements using chemical double mutant cycles show that electron-withdrawing groups lead to remarkable increases in the stability of aromatic interactions, and the magnitude of the effect is much larger in water than in chloroform solution.

## Graphical abstract

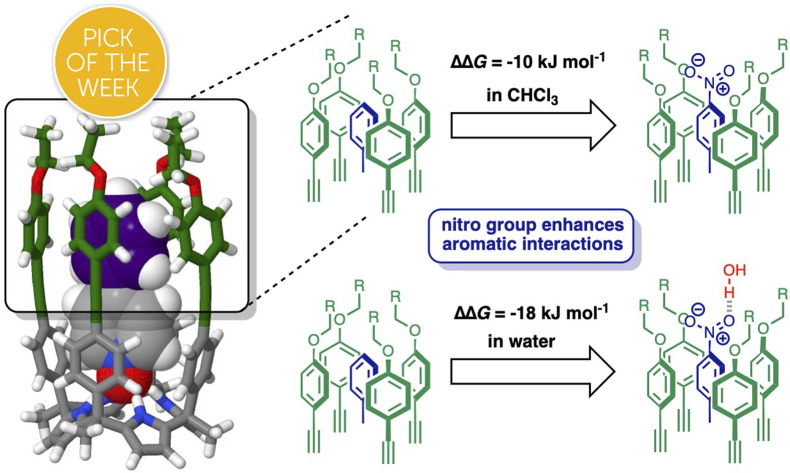

## Introduction

Non-covalent interactions govern many of the phenomena of contemporary interest in chemistry, biology, and materials science.^1,2^ Interactions between aromatic rings are one of the most important classes of intermolecular interaction, but they are particularly sensitive to changes in solvent, interaction geometry and the nature and location of substituents, which has hampered the development of a unified understanding.^3–6^ Although aromatic rings are generally considered non-polar, the 1990 paper by Hunter and Sanders brought to the fore the importance of electrostatics in determining how the thermodynamic properties of aromatic interactions depend on both the substituents and the orientation of the two rings, and this idea was confirmed by subsequent studies using higher levels of theory.^7,8^

A range of different supramolecular systems have been developed to quantify aromatic interactions in organic solvents.^9–38^ Experimental measurements of substituent effects on both edge-to-face and stacking interactions using chemical double mutant cycles and molecular torsion balances correlate well with Hammett substituent constants, confirming a major role for electrostatic interactions in aromatic interactions in non-polar solvents. However, it is not clear whether such effects persist in more polar solvent environments like water.^39^ It is well-established that aromatic interactions are more favourable in water than in organic solvents, due to the solvophobic contribution to the solvation free energy (hydrophobic effect),^40,41^ but substituent effects have not been quantified. A key question is to what extent the polar solvent damps the thermodynamic contribution from electrostatic interactions.

Here we describe a supramolecular system for measuring aromatic interactions in water and show that substituents have a remarkable effect on interaction strength, with an increase of three orders of magnitude in the stability of a complex when a single nitro group is added to one of the aromatic rings. The role of desolvation in enhancing substituent effects in water has been quantified by comparing the measurements with the same experiments carried out in chloroform.

## Approach

**Fig. 1:**
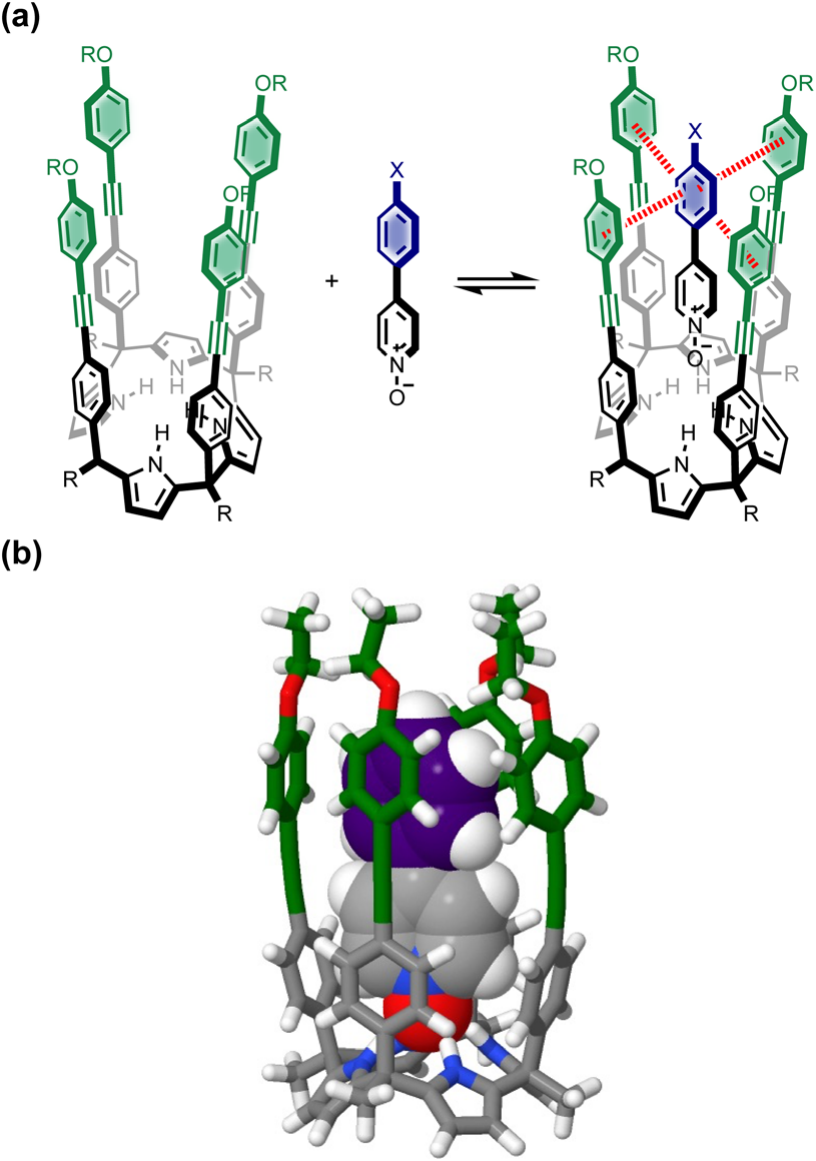
(a) Complex formed by a super-aryl-extended calix[4]pyrrole and a 4-arylpyridine *N*-oxide, highlighting aromatic interactions between the blue ring of the guest and the four green rings of the host. (b) Energy-minimized structure of the complex (X = H). Geometry optimizations were performed at the RI-BP6-D3BJ-def2-TZVP level of theory with COSMO water continuum model as implemented in TURBOMOLE v7.0 2015, a development of University of Karlsruhe and Forschungszentrum Karlsruhe GmbH, 1989–2007, TURBOMOLE GmbH, since 2007; available from http://www.turbomole.com.^43–52^

Fig. 1 shows the structure of the complex formed by a super-aryl-extended calix[4]pyrrole and a 4-arylpyridine *N*-oxide.^42^ H-bonding interactions between the pyrrole NH protons and the *N*-oxide group lock the host in the cone conformation and fix the guest in a well-defined location within the cavity created by the aromatic side-arms of the calix[4]pyrrole. This binding geometry creates a network of aromatic interactions between the host and guest. Here we take advantage of this system to measure substituent and solvent effects on the aromatic interactions between the four aromatic rings of the host (green) and the aromatic ring of the guest (blue) highlighted in Fig. 1.

**Fig. 2:**
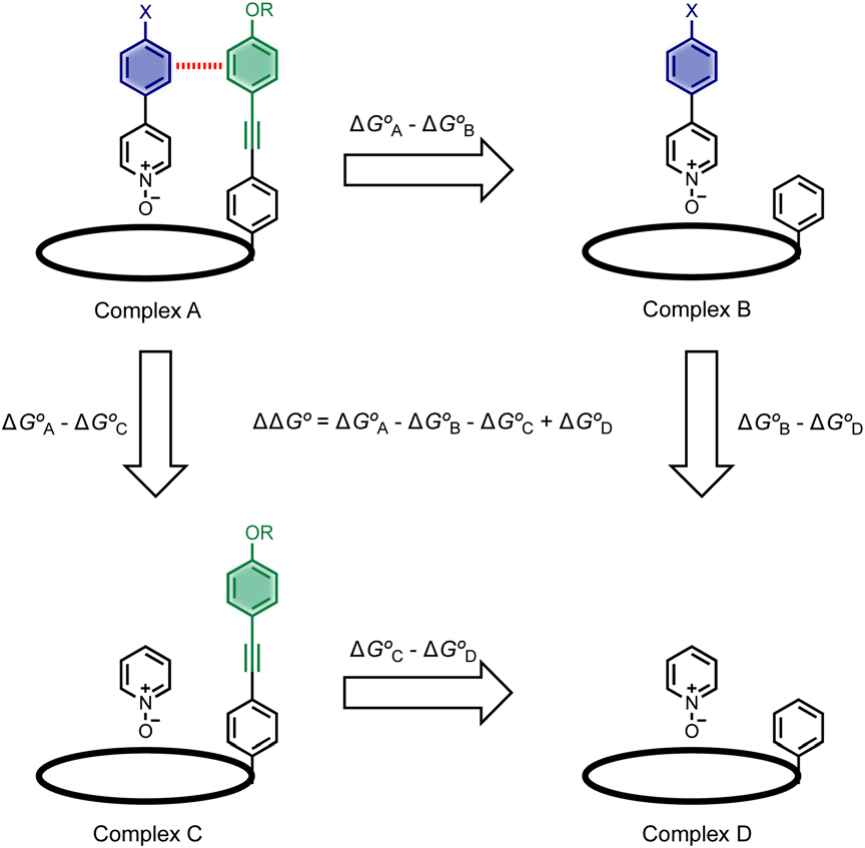
Chemical double mutant cycle for evaluating the free energy contribution (ΔΔ*G*°) due to aromatic interactions with the blue ring of the guest to the overall stability of complex A. Only one of the four calix[4]pyrrole side-arms is shown for clarity, but the DMC measures the sum of interactions with four green aromatic rings on the host.

Chemical double mutant cycles (DMC) have been widely used to dissect out the thermodynamic contributions of individual functional group interactions to the overall stability of a complex.^53^Fig. 2 illustrates how this approach is applied to the calix[4]pyrrole complex shown in Fig. 1. Fig. 1b shows that there are two edge-to-face interactions and two stacking interactions between the blue aromatic ring of the guest and the four green aromatic rings of the host. For the purposes of illustration, complex A in Fig. 2 shows only one green ring, but the DMC measures the sum of all four interactions.

In principle, the contribution of the aromatic interactions to the stability of complex A might be estimated by comparison with complex C, which lacks the blue aromatic ring. However, there are additional contributions to this free energy difference. For example, the X substituent on the blue aromatic ring in complex A changes the H-bond acceptor properties of the pyridine *N*-oxide (see below), so the difference between complexes A and C contains a contribution from the change in H-bond strength as well as the aromatic interactions. However, comparison of complexes B and D of the DMC provides a measurement of the free energy contribution due the change in H-bond strength in the absence of the aromatic interactions of interest.

Thus eqn (1) can be used to obtain a measurement of the free energy contribution of the four aromatic interactions between the blue and green rings in complex A without any complications due to changes in H-bond strength.1
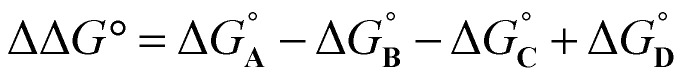


## Results and discussion

### Synthesis

**Fig. 3:**
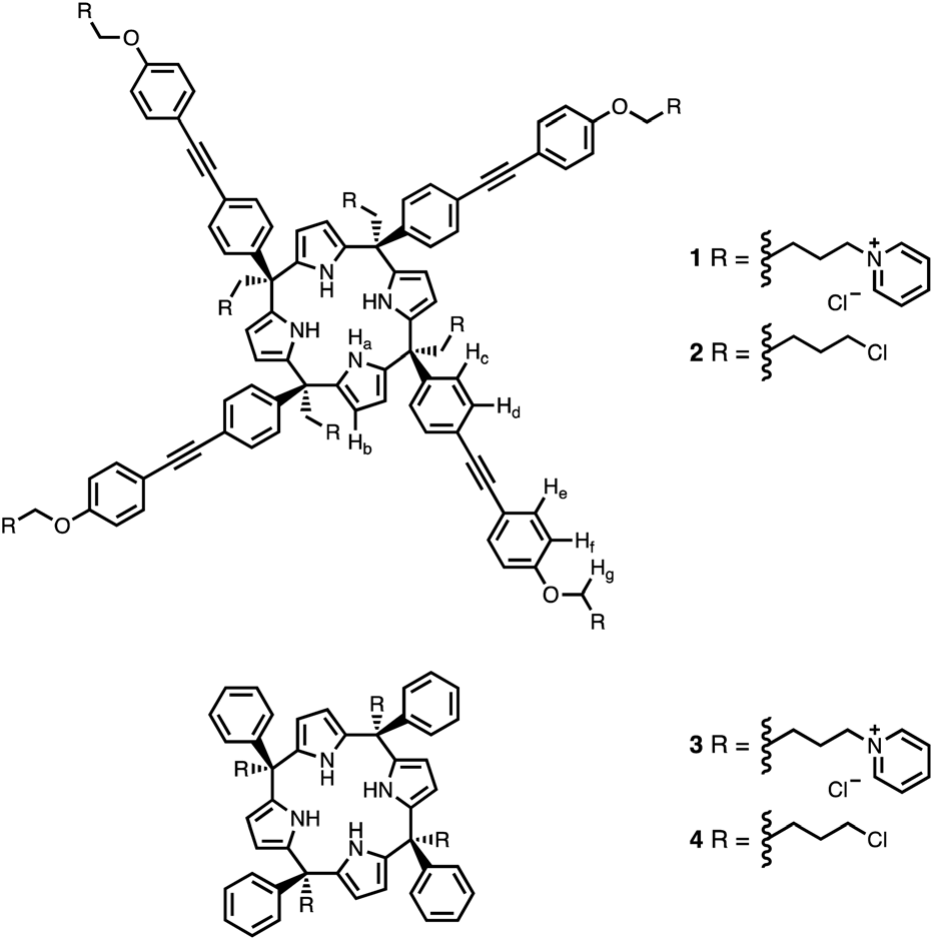
Chemical structures of calix[4]pyrroles 1–4 with the ^1^H NMR proton labelling scheme.

Calix[4]pyrrole receptors 1–4 shown in Fig. 3 were synthesised according to previously reported procedures.^42,54,55^

**Scheme 1:**
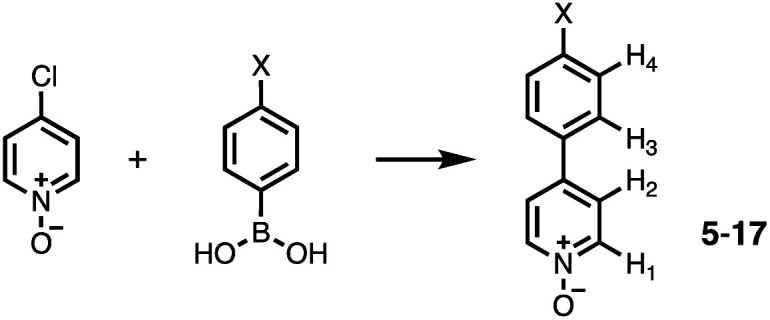
Synthesis of 4-arylpyridine *N*-oxides. Reagents and conditions: Pd(PPh_3_)_4_, Na_2_CO_3_, dioxane/H_2_O, 80 °C, 16 h. X = H (5, 69%), Me (6, 79%), NMe_2_ (7, 73%), CF_3_ (8, 70%), NO_2_ (9, 75%), OMe (10, 66%), CHO (11, 65%), COMe (12, 79%), iPr (13, 69%), Et (14, 73%), F (15, 14%), Cl (16, 41%), Br (17, 28%). The ^1^H NMR proton labelling scheme is shown and when X = H the label is H_5_.

A series of pyridine *N*-oxide guests (5–17) were each prepared in one step from the corresponding boronic acid and 4-chloropyridine *N*-oxide (Scheme 1). The parent guest, pyridine *N*-oxide (PNO), is commercially available.

### Binding studies

**Fig. 4:**
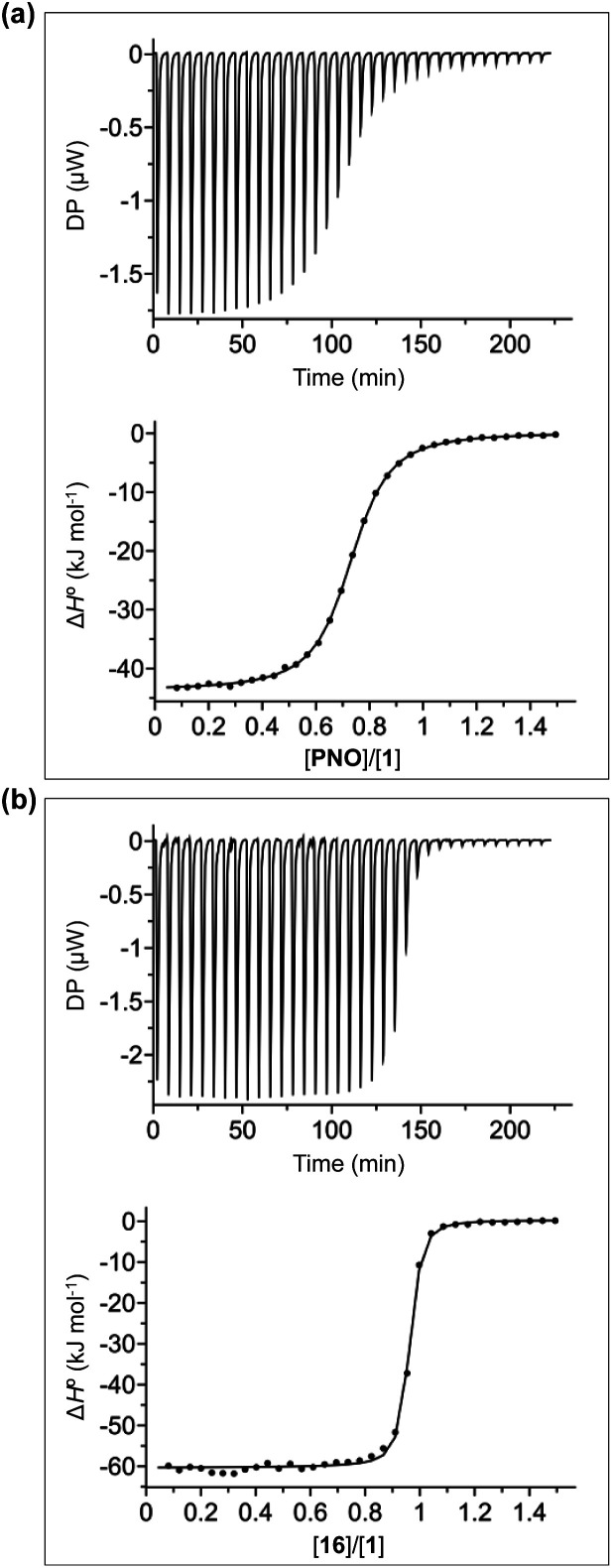
ITC data for titration of (a) PNO (0.28 mM) into 1 (0.04 mM) and (b) 16 (0.28 mM) into 1 (0.04 mM) in water at 298 K. The raw data for each injection is shown (differential power, DP), along with the least-squares-fit of the enthalpy change per mole of guest (Δ*H*°) to a 1 : 1 binding isotherm.

The thermodynamic properties of all pairwise combinations of all four calix[4]pyrrole hosts and all fourteen pyridine *N*-oxide guests were investigated using isothermal titration calorimetry (ITC) experiments. The binding properties of the pyridinium derivatives 1 and 3 were investigated in water, and the corresponding neutral receptors 2 and 4 were studied in chloroform solution. Fig. 4a shows typical titration data for complexes B, C and D of the DMC. The smooth sigmoidal transition from free to bound states in Fig. 4a means that the data can be fitted to a 1 : 1 binding isotherm to obtain both the association constant (*K*) and enthalpy change for formation of the 1 : 1 complex (Δ*H*°). The corresponding free energy change (Δ*G*° = −*RT* ln *K*) and entropy change (*T*Δ*S*° = Δ*H*° + *RT* ln *K*) associated with formation of the 1 : 1 complex can be calculated directly from these measurements (see Tables S1 and S2†).

Fig. 4b shows typical ITC data for complex A of the DMC. In this case, the additional aromatic interactions lead to a significant increase in the stability of the complex, and there is a sharp transition at one equivalent of guest, which means that the association constant is too high to be accurately measured under these conditions (C-values > 10^6^). However, this experiment does provide an accurate measurement of the enthalpy change for formation of the 1 : 1 complex (Δ*H*°). In principle, it should be possible to measure the association constant by working at a lower concentration of host, but the ITC instrument is not sufficiently sensitive to measure the heat released at the required concentrations. Association constants for complex A of the DMC were therefore measured using ^1^H NMR competition experiments. For the complexes formed by 4, where both ITC and NMR competition experiments are possible, the measured association constants are in good agreement, confirming the validity of different methods (see Fig. S108†).^56^

**Fig. 5:**
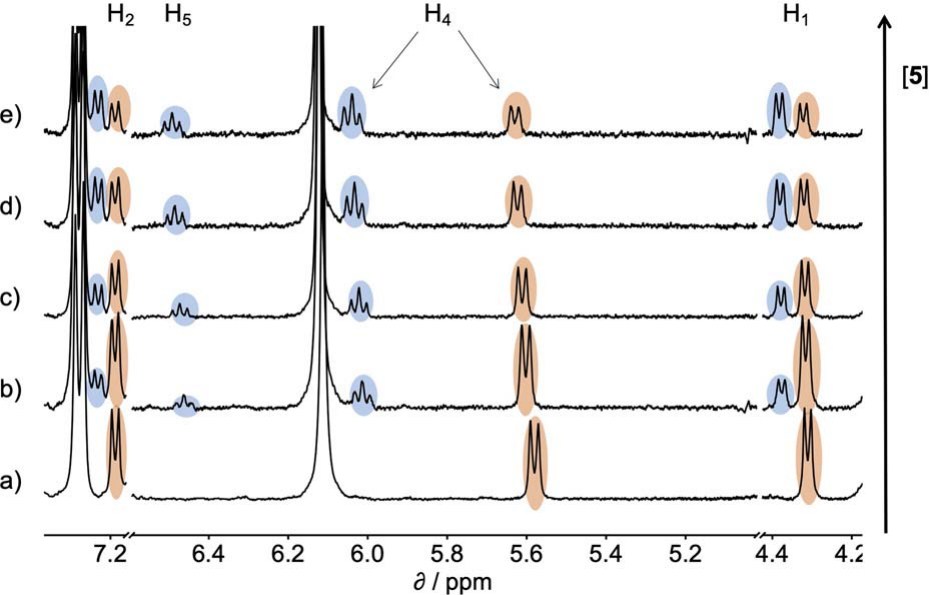
Partial 400 MHz ^1^H NMR for titration of 5 into a mixture of 1 and 6 in D_2_O at 298 K. (a) 1: 0.53 mM; 6: 0.80 mM; 5: 0 mM; (b) 1: 0.37 mM; 6: 0.56 mM; 5: 0.82 mM; (c) 1: 0.30 mM; 6: 0.46 mM; 5: 1.20 mM; (d) 1: 0.21 mM; 6: 0.32 mM; 5: 1.68 mM; (e) 1: 0.16 mM; 6: 0.25 mM; 5: 1.93 mM. Signals due to the 1·5 complex are highlighted in blue, and signals due to the 1·6 complex are highlighted in orange. See Scheme 1 for the proton labelling scheme.

^1^H NMR spectra were recorded for mixtures of each of guests 5–17 and either 1 in D_2_O or 2 in deuterochloroform. In all cases, the signals due to the free and bound species were in slow exchange on the ^1^H NMR timescale. The association constant for the 1·5 complex in D_2_O was previously reported,^42^ so this complex was used as a reference point for the measurement of association constants by guest competition experiments. Fig. 5 shows typical data from a ^1^H NMR titration. Addition of 6 to 1 lead to quantitative formation of the 1 : 1 complex, because the association constant is so high. When small amounts of the second guest 5 were added to this mixture, three sets of signals were observed, corresponding to the two different host·guest complexes, 1·5 and 1·6, and free 6, all in slow exchange. As increasing concentrations of 5 were added, the signals due to the 1·6 complex (highlighted in orange in Fig. 5) decreased in intensity, the signals due to free 6 increased, and the signals due to the 1·5 complex (highlighted in blue) increased. These observations show that 5 displaces 6 from the receptor and that both the 1·5 and 1·6 complexes are present in equilibrium. The integrals of the ^1^H NMR signals (*I*) can be used to directly measure the concentrations of all species present and hence determine the equilibrium constant for guest exchange (eqn (2)).2
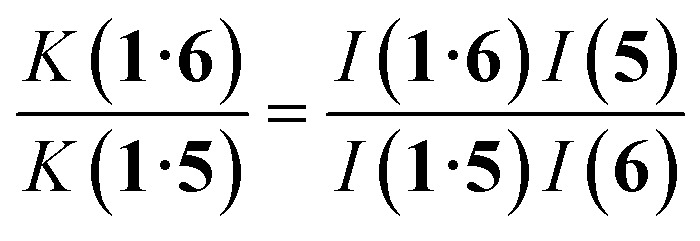


In this case, one of the two association constants *K*(1·5) is known, so the association constant for the 1·6 complex can be determined by averaging the values obtained from eqn (2) for each spectrum of the titration. One limitation of this approach is that both complexes must coexist in equilibrium with the free guests, which places an upper limit on the ratio of association constants that can reliably be measured. However, if a range of different guests with different binding affinities are used, it is possible to carry out a series of pairwise competition experiments to step up an affinity ladder and measure association constants that are significantly higher than the starting reference point. For receptor 1 in water, the reference point was the 1·5 complex, and for receptor 2 in chloroform, the reference point was the 2·PNO complex, for which the association constant had been measured using ITC. The association constants (*K*) measured using pairwise NMR competition experiments are reported in Tables S1 and S2,† along with the corresponding free energy (Δ*G*°) and entropy changes (Δ*S*°), which were calculated from *K* and Δ*H*°.

**Fig. 6:**
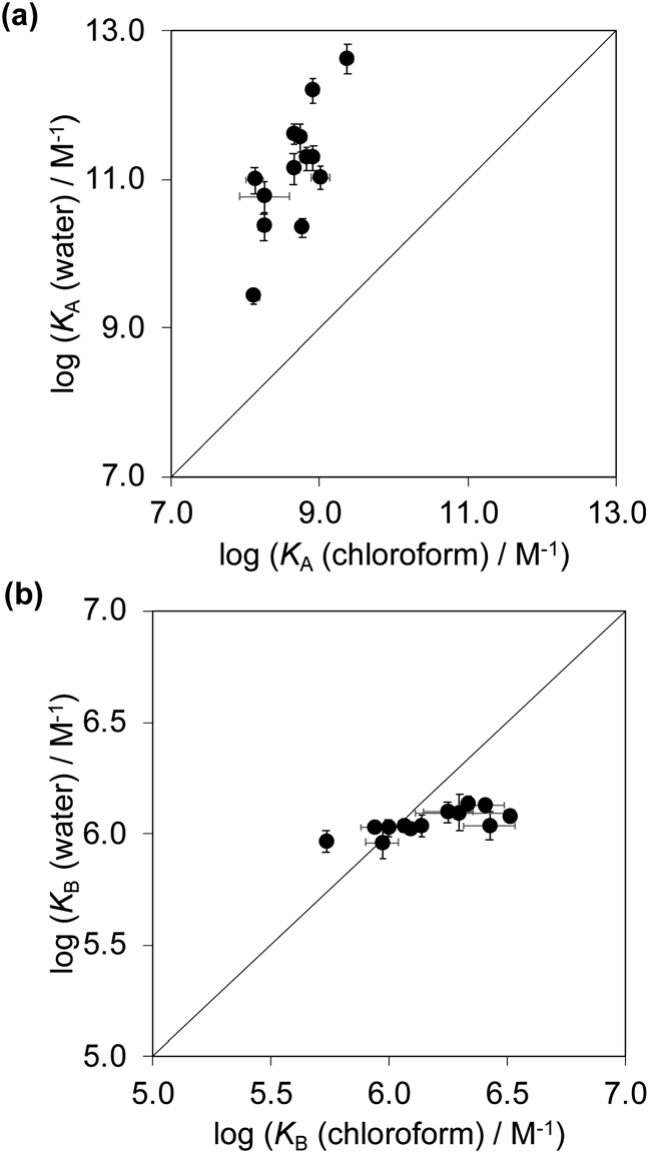
Association constants measured in water compared with the corresponding values measured in chloroform at 298 K for formation of (a) complex A and (b) complex B of the DMC. The line corresponds to *y* = *x* in each case.

Fig. 6a compares the association constants for formation complex A of the DMC in water with the corresponding values measured in chloroform, and Fig. 6b shows the same comparison for complex B of the DMC. All of the association constants for complex A are significantly larger than the values for complex B. In complex A, the green aromatic rings of the receptor highlighted in Fig. 2 interact with the blue aromatic ring of the pyridine *N*-oxide guest, but in complex B, the green aromatic interactions have been removed, and this leads to a large decrease in stability both in water and in chloroform. Fig. 6a also shows that when the aromatic interactions are present in complex A, the association constants in water are about two orders of magnitude higher than the corresponding values measured in chloroform. These results show that aromatic interactions make a substantial contribution to the stability of complex A, and that the solvation/desolvation processes that occur in water further enhance the contribution to binding affinity. The entropy changes measured in water are all significantly less negative than in chloroform, which is indicative of the role of the hydrophobic effect in increasing the association constants observed in water (see Fig. S109†).^57^

**Fig. 7:**
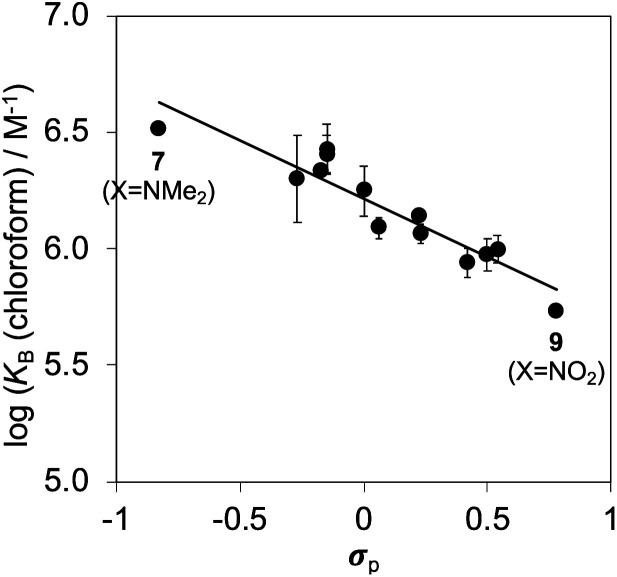
Association constants for formation of complex B of the DMC measured in chloroform at 298 K plotted as a function of the Hammett constant for substituent X (*σ*_p_). The line of best fit is log *K*_B_ = −0.5*σ*_p_ + 6.2 (*R*^2^ = 0.87).

Fig. 6b shows that the association constants measured for complex B in water are practically identical and do not depend on the identity of the X substituent on the guest aromatic ring. However, the association constants measured for complex B in chloroform depend on the nature of X and span almost one order of magnitude. The X substituent is in the *para* position relative to the pyridine *N*-oxide substituent on the guest aromatic ring, so there is some conjugation with the nitrogen atom through the biphenyl linkage. Fig. 7 shows that the values of the association constants measured for complex B in chloroform correlate well with the Hammett constants for the X substituent.^58^ Electron-donating substituents increase the binding affinity, presumably because they increase the H-bond acceptor strength of the pyridine *N*-oxide oxygen atom.^59^ This effect is not apparent in water, because any increase in H-bond strength in the complex is cancelled out by a similar increase in the strength of the H-bonds made between the pyridine *N*-oxide and the aqueous solvent in the free state. The differences in H-bond strength observed for complex B also contribute to the differences in association constant measured for complex A in chloroform. However, the DMC is based on the difference between the stabilities of these two complexes, and so any contributions due to differences in H-bond strength cancel out.

### Three-dimensional structures of the complexes

Aromatic interactions are sensitive to geometry, so before any substituent effects can be interpreted, it is important to establish whether there are conformational differences between the complexes that lead to differences in interaction geometry. ^1^H NMR spectra provide a sensitive experimental probe of the geometries of the aromatic interactions in these systems, because there are very large complexation-induced changes in chemical shift (Δ*δ*) associated with the ring currents of the aromatic rings. The Δ*δ* values measured for complex A are practically identical for all 13 guests in deuterium oxide and deuterochloroform (see Table S3†). The guest proton *ortho* to the *N*-oxide group (H_1_) shows a consistent complexation-induced change in chemical shift of −4.0 ppm in water and −3.7 ppm in chloroform, which indicates that the geometry of the H-bonding interaction at the base of the complex is the same in all of the complexes. The guest proton *ortho* to the X substituent (H_4_) shows a consistent complexation-induced change in chemical shift of −2.0 ppm in water and −1.5 ppm in chloroform, which indicates that the geometry of the aromatic interactions at the other end of the complex is the same in all of the complexes. Similarly, the chemical shifts of the ^1^H NMR signals due to the host vary by less than 0.1 ppm from one complex to another (see Table S4†), which confirms that all of the complexes have essentially identical three-dimensional structures.

**Fig. 8:**
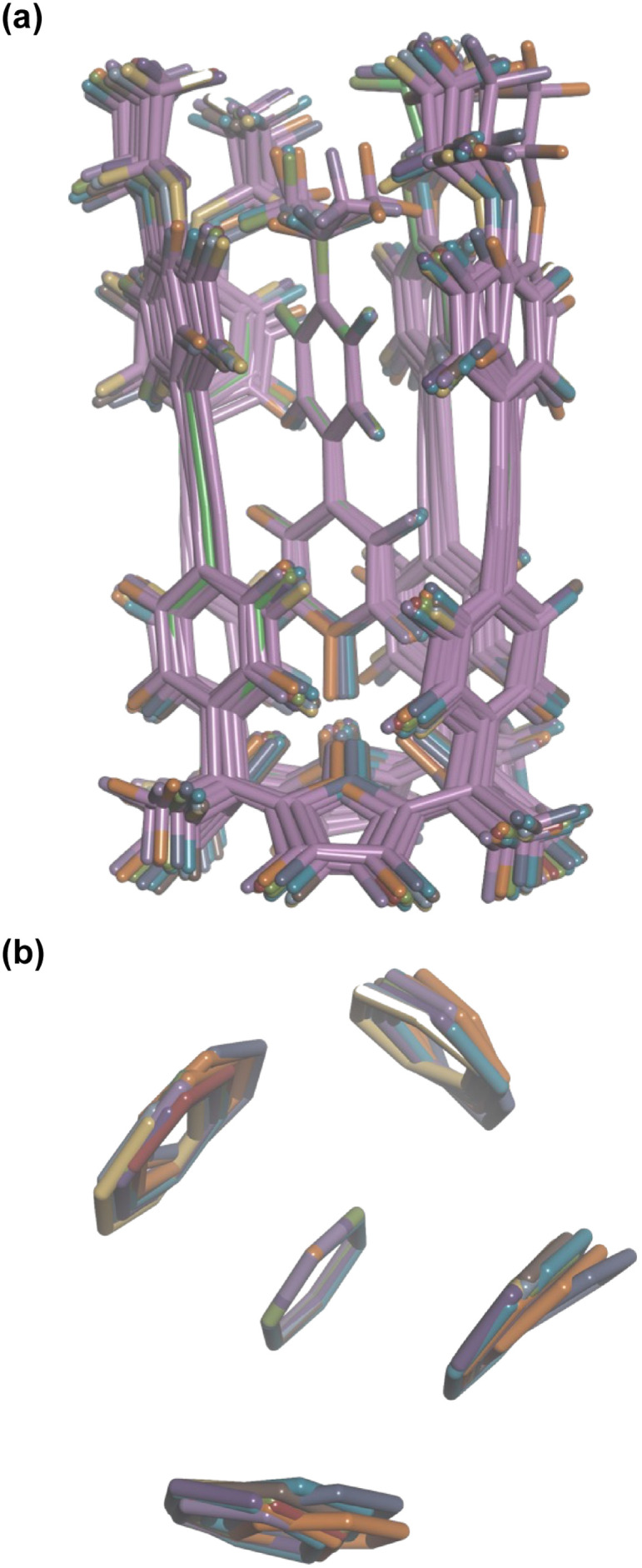
Overlay of the three-dimensional structures of complex A of the DMC with 13 different X substituents using Discovery Studio Visualizer v21.1.0.2098, Dassault Sistèmes Biovia Corp. https://discover.3ds.com/discovery-studio-visualizer-download. (a) Complete structure of the complex. (b) Top view of the geometry of the four aromatic interactions measured by the DMC. The angles between two interacting rings and centre-to-centre distances are 90–127° and 4.6–5.0 Å for the edge-to-face interactions, and 1–5° and 3.5–3.7 Å for the stacking interactions. The centre-to-centre offset distances for stacking interactions are 1.3–1.7 Å. In edge-to-face interactions, the distances of the centroids of the host rings to the perpendicular plane defined by the guest ring are 0.8–2.0 Å. The energy minimized structures of the complexes were obtained at the RI-BP6-D3BJ-def2-TZVP level of theory with COSMO water continuum model as implemented in TURBOMOLE v7.0 2015, a development of University of Karlsruhe and Forschungszentrum Karlsruhe GmbH, 1989–2007, TURBOMOLE GmbH, since 2007; available from http://www.turbomole.com.^43–52^

This conclusion is supported by DFT calculations. Fig. 8a shows an overlay of the minimum energy structures of complex A calculated for each of the 13 different X substituents, and Fig. 8b highlights the geometry of the aromatic interactions that are measured by the DMC. It is clear that the calix[4]pyrrole framework locks the guests into a very well-defined binding pocket, and the interaction geometry does not change to any significant extent as the nature of the substituent X is varied. In other words, substituent effects measured by the DMC can be ascribed to intermolecular interactions rather than conformational changes.

### Chemical double mutant cycle analysis

**Fig. 9:**
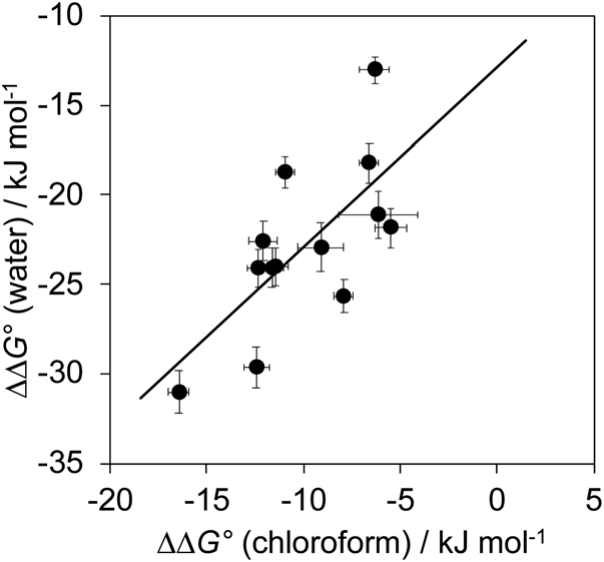
The free energy contributions due to aromatic interactions in complex A (ΔΔ*G*°) measured using DMCs in water compared with the corresponding measurements in chloroform. The line of best fit is shown (*R*^2^ = 0.49).

The total free energy contribution (ΔΔ*G*°) due to the four aromatic interactions in complex A in water and in chloroform was determined using eqn (1). The results are reported in Table 1. All interactions are attractive, but there are very large substituent and solvent effects on interaction strength. The values of ΔΔ*G*° in water range from −13 kJ mol^−1^ to −29 kJ mol^−1^ and are more attractive than the corresponding values measured in chloroform, which range from −6 kJ mol^−1^ to −17 kJ mol^−1^. Fig. 9 shows that there is little correlation between the results obtained in the two solvents and indicates that solvent effects in this system are more complicated than simply an additional stabilisation due to the hydrophobic effect in water.

**Table 1:** Free energy contributions (ΔΔ*G*°/kJ mol^−1^) to the stability of complex A of the DMC due to the four aromatic interactions highlighted in Fig. 1

Guest	X	Solvent	
		Water	Chloroform
5	H	−13 ± 1	−6 ± 1
6	Me	−18 ± 1	−7 ± 0
7	NMe_2_	−26 ± 1	−8 ± 0
8	CF_3_	−24 ± 1	−12 ± 1
9	NO_2_	−31 ± 1	−16 ± 1
10	OMe	−23 ± 1	−9 ± 1
11	CHO	−24 ± 1	−12 ± 1
12	COMe	−30 ± 1	−12 ± 1
13	iPr	−22 ± 1	−5 ± 1
14	Et	−21 ± 1	−6 ± 2
15	F	−19 ± 1	−11 ± 0
16	Cl	−23 ± 1	−12 ± 1
17	Br	−24 ± 1	−11 ± 1

A similar DMC analysis can be carried out to determine the enthalpy (ΔΔ*H*°) and entropy changes (*T*ΔΔ*S*°) associated with the aromatic interactions in complex A. There is no correlation between these measurements and the values of ΔΔ*G*°. However, entropy–enthalpy compensation is observed when the values of ΔΔ*H*° and *T*ΔΔ*S*° are compared, and the correlation is even stronger for the raw Δ*H*° and *T*Δ*S*° measurements (*R*^2^ values are 0.8–0.9, see Fig. S110 and S111†).

**Fig. 10:**
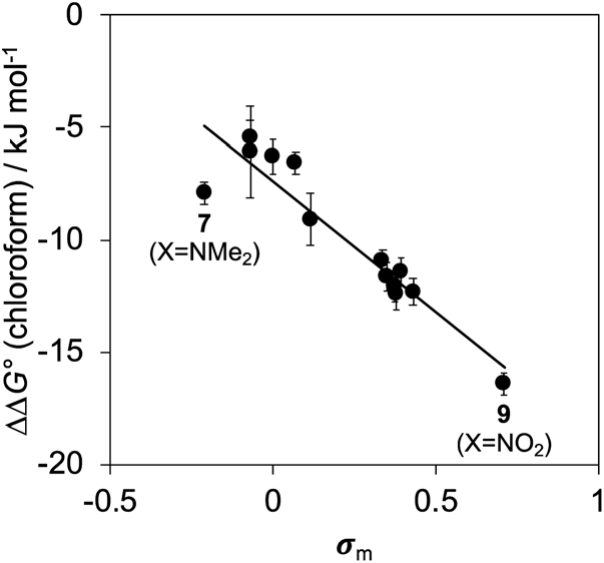
The free energy contributions due to aromatic interactions in complex A of the DMC in chloroform (ΔΔ*G*°) plotted as a function of the Hammett constant for substituent X (*σ*_m_). The line of best fit is ΔΔ*G*° = −11.7*σ*_m_ − 7.4 kJ mol^−1^ (*R*^2^ = 0.88).

Fig. 10 shows a Hammett plot of the results in Table 1 for the measurements made in chloroform. The correlation with the *meta* Hammett parameter was significantly better than with the *para* parameter, which is consistent with literature reports that the *meta* parameter provides a superior description of the electrostatic potential on the surfaces of aromatic rings^31^ (note that the *para* parameter was used above to analyse substituent effects on H-bonding, because there is direct conjugation with the *para* H-bond acceptor group). The results in Fig. 10 suggest that substituent effects in chloroform are dominated by differences in electrostatic interactions between the aromatic rings. For the two edge-to-face interactions in complex A, an electron-withdrawing substituent (X) on the blue aromatic ring in Fig. 1 makes the protons on the edge of the ring more positive, which would enhance attractive electrostatic interactions with the negative π-faces of the green aromatic rings of the receptor.^7^ For the two stacking interactions, an electron-withdrawing substituent (X) on the blue aromatic ring makes the π-face of the ring less negative and reduces the repulsive electrostatic interactions with the negative π-faces of the green aromatic rings of the receptor. As a result, electron-withdrawing substituents at X stabilise complex A, and electron-donating substituents at X destabilise complex A. These electrostatic effects provide a straightforward rationalisation of the variation in the stabilities of the complexes observed in chloroform, and the magnitude of the substituent effects is consistent with previous studies in chloroform solution (see Fig. S112†).^13,23^

There is no relationship between Hammett substituent constants and the values of ΔΔ*G*° measured in water for the aromatic interactions in complex A (see Fig. S113†). This result suggests that there are additional substituent effects on desolvation of the guests when they bind to the receptor in water. Substituent effects on solvation energies can be quantified using experimental measurements of the free energy of transfer of the corresponding aromatic compounds (PhX) from water into *n*-hexadecane 
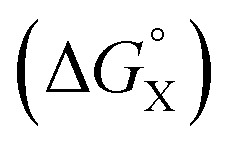
.^60^ The values of 
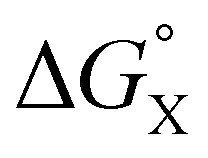
 provide a measure of the hydrophobicity of the blue aromatic ring of the pyridine *N*-oxide guest, but there is no clear relationship with the values of ΔΔ*G*° measured in water (see Fig. S114†). Indeed, the substituents that lead to the most favourable aromatic interactions in water are the least hydrophobic functional groups.

**Fig. 11:**
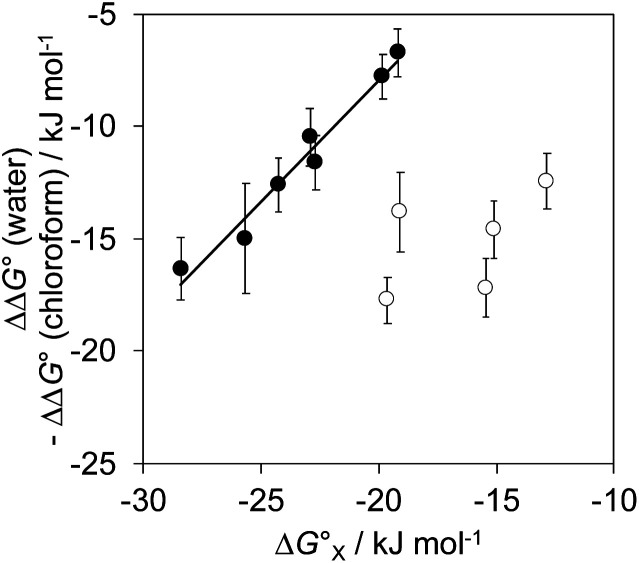
The difference between the water and chloroform DMC measurements of the free energy contributions due to aromatic interactions in complex A (ΔΔ*G*°) compared with the corresponding values for the free energy of transfer of PhX from water into *n*-hexadecane 
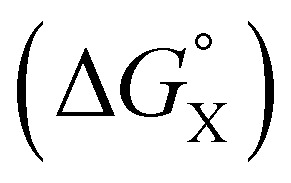
. Data for non-polar X substituents are shown as filled circles (H, Me, Et, iPr, F, Cl, Br) and open circles for polar X substituents (CHO, COMe, NO_2_, NMe_2_, OMe). The line of best fit shown for non-polar substituents is *y* = 1.1*x* + 13.7 kJ mol^−1^ (*R*^2^ = 0.97).

**Fig. 12:**
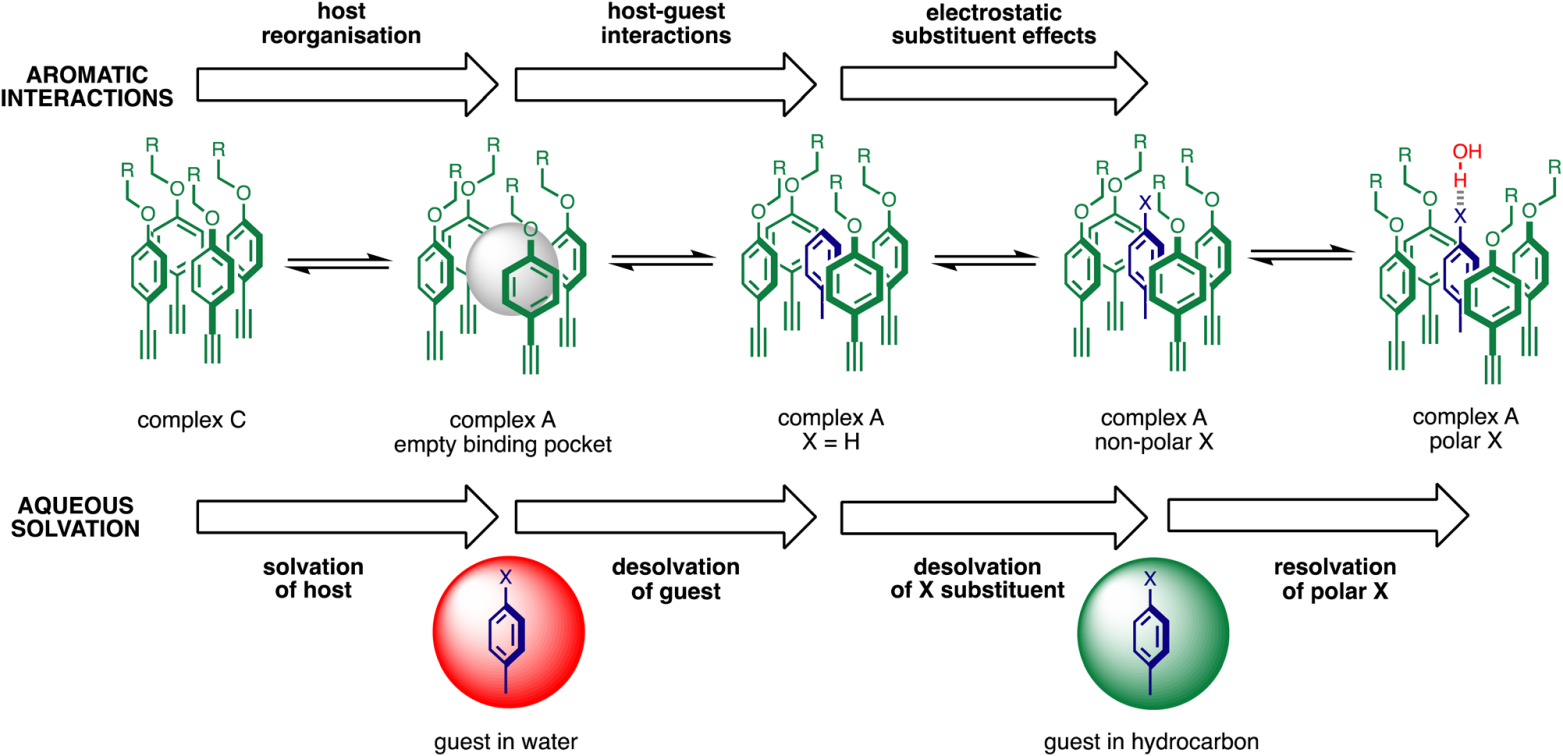
Factors that determine the net free energy contribution due to aromatic interactions in complex A of the DMC in water (ΔΔ*G*°). The free energy contributions highlighted as aromatic interactions were measured in chloroform, and additional free energy contributions highlighted as aqueous solvation were measured in water. The difference between complexes C and A involves reorganisation of the green aromatic rings around the binding pocket, and the difference between complex A formed with non-polar and polar X substituents on the guest (blue) involves reorganisation of the alkoxy groups on the rim of the binding pocket to allow interaction of X with the solvent.

The contribution of aqueous solvation to the aromatic interactions in complex A can be more directly assessed by using the difference between the values of ΔΔ*G*° measured in water and in chloroform. Fig. 11 shows a plot of this free energy difference *versus*
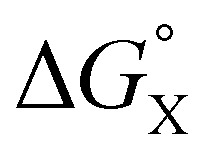
 for the transfer of PhX from water into *n*-hexadecane. Two distinct types of behaviour can be identified: a linear relationship is observed for non-polar substituents (X = H, Me, Et, iPr, F, Cl, Br) but not for polar substituents (X = CHO, COMe, NO_2_, NMe_2_, OMe). For non-polar substituents, the slope of the best fit straight line is one. This result indicates that the substituent effects on the stability of complex A in water are a simple combination of the electrostatics of the aromatic interactions, which are identical to the substituent effects measured in chloroform, and the hydrophobicity of the X group, which is identical to the substituent effects on transfer from water into *n*-hexadecane 
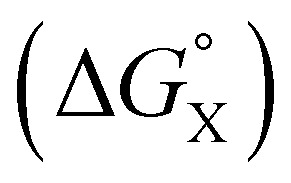
. The slope of one in Fig. 11 also means that complexation involves complete desolvation of non-polar guests, which can be fully encapsulated inside a hydrophobic cavity, if the alkoxy substituents on the rim of the calix[4]pyrrole binding pocket fold over the substituent to exclude water (see complex A non-polar X in Fig. 12).

For polar substituents (X = CHO, COMe, NO_2_, NMe_2_, OMe), the values of ΔΔ*G*° are significantly more negative than the correlation obtained for non-polar substituents would predict. This result indicates that there are additional stabilising interactions in water, and the most likely explanation is that the polar groups are not fully desolvated when they bind to the receptor. Transfer from water into *n*-hexadecane involves complete desolvation of the substituent, but if the alkoxy substituents on the rim of the calix[4]pyrrole rotate away from the binding pocket, the X substituent can be exposed to the solvent. Thus it is possible for polar substituents to maintain some H-bonding interactions with the solvent when complex A is formed in water (see complex A polar X in Fig. 12).

**Fig. 13:**
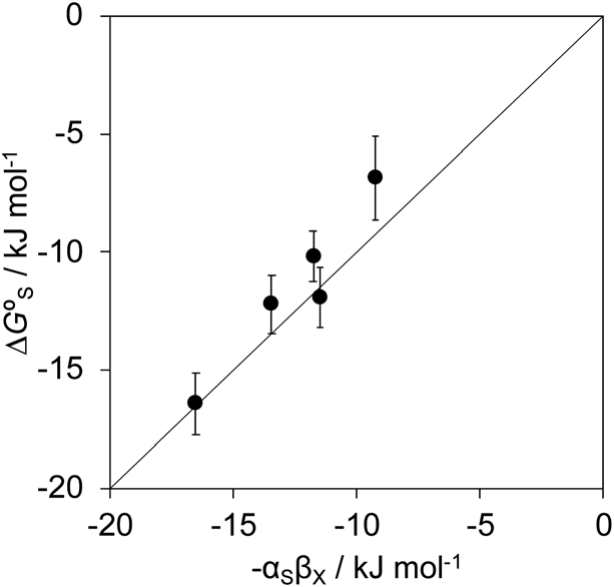
Experimentally measured stabilisation of complex A in water due to resolvation of the guest substituent X 
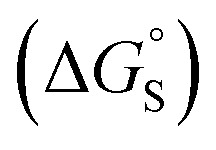
 compared with the calculated free energy change due formation of a H-bond between X and water (−*α*_S_*β*_X_). The line corresponds to *y* = *x*.

Polar interactions with the solvent lead to a significant stabilisation compared with the expectations based on complete desolvation of the guest. The deviation of polar substituents from the trendline obtained for non-polar substituents in Fig. 11 can be used to calculate the stabilisation energy due to the resolvation process illustrated in Fig. 12 (
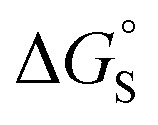
 for resolvation of polar X). The polar substituents involved are all H-bond acceptors, and the free energy for formation of a H-bond with water can be estimated using the product of the H-bond acceptor parameter for the functional group (*β*_X_) and the H-bond donor parameter for water (*α*_S_ = 2.8).^2,59^Fig. 13 shows that the stabilisation energy associated with resolvation of polar guests 
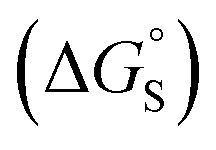
 is remarkably well-predicted by the free energy for formation of a H-bond with water (−*α*_S_*β*_X_). We conclude that the flexibility of the alkoxy groups around the rim of the binding pocket allows the non-polar side chains to rotate out of the way, so that water can H-bond to polar X substituents in complex A (Fig. 12).

Fig. 12 illustrates the free energy contributions due aromatic interactions measured by the DMC in chloroform and the additional free energy contributions due to aqueous solvation in water. X-ray crystal structures of acetonitrile complexes of super-aryl-extended calix[4]pyrroles show that there is no binding pocket at the top of the cavity.^61–63^ Instead, the green aromatic rings highlighted in Fig. 1 collapse to make close contacts with each other, and molecular models indicate that same arrangement is found in complex C of the DMC (see complex C in Fig. 12). In order to create a binding pocket that will accommodate the blue aromatic ring of the guest in complex A, the green aromatic rings on the calix[4]pyrrole have to open out. The energy penalty for the host to adopt this high energy conformation (*i.e.* complex A empty binding pocket in Fig. 12) is difficult to estimate, but it involves breaking multiple interactions between the green aromatic rings in the collapsed conformation shown for complex C in Fig. 12. The loss of the host–host aromatic interactions is more than compensated for by the host–guest interactions that are made between the blue aromatic ring of the guest and the green aromatic side-walls of the cavity in complex A, so the values of ΔΔ*G*° measured for all of the guests are negative in chloroform.

Reorganisation of the binding pocket not only creates a cavity inside the receptor, it also increases the surface area of the green aromatic rings that are exposed to water on the outside of the receptor (see complex A empty binding pocket in Fig. 12). The DFT models of the structures indicate that the difference between the total surface areas of complexes A and C is about 20 Å^2^ when X = H, and solvation of this increased hydrophobic surface is unfavourable in water. However, the difference between the surface areas of PNO and guest 5 is about 70 Å^2^,^42^ so desolvation of the hydrophobic phenyl group of the guest more than compensates solvation of the host. The value of ΔΔ*G*° measured for X = H in water is −7 kJ mol^−1^ more favourable than in chloroform, which is consistent with the burial of 50 Å^2^ of hydrophobic surface (estimates from complexation and phase transfer free energies give a value of about 0.15 kJ mol^−1^ Å^−2^).

**Fig. 14:**
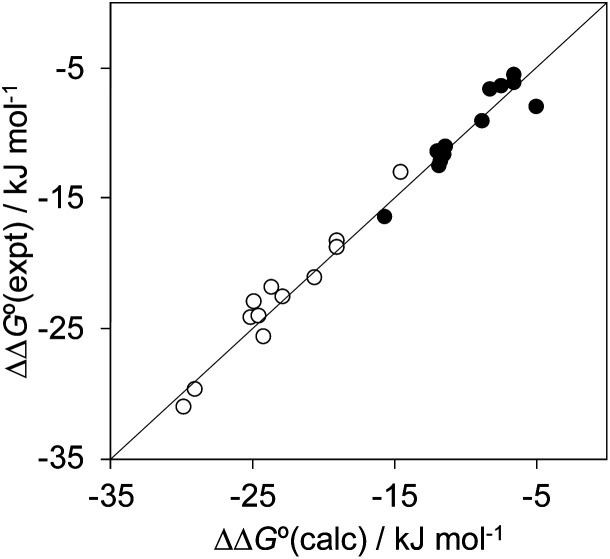
Comparison of the experimentally measured free energy contributions due to aromatic interactions in complex A of the DMC with the values calculated using eqn (3) for the experiments carried out in chloroform (black) and eqn (4) for the experiments carried out in water (white). The line corresponds to *y* = *x* (RMSE = 1.1 kJ mol^−1^).

Fig. 12 highlights the complexity of molecular recognition in water. In addition to the host–guest interactions that appear to dictate binding, the observed association constants reflect changes in the conformation of the receptor, changes in solvation of the receptor, and changes in solvation of the guest. The Hammett correlation in Fig. 10 accounts for the substituent effects on the aromatic interactions measured in chloroform (eqn (3)). For the aromatic interactions measured in water, the contributions due to aqueous solvation illustrated in Fig. 12 must be added to give eqn (4). Fig. 14 shows that eqn (3) and (4) provide an excellent description of substituent effects on aromatic interactions in chloroform and in water respectively (RMSE = 1.1 kJ mol^−1^).

3 ΔΔ*G*° (chloroform)/kJ mol^−1^ = −7.4 − 11.7*σ*_X_

4
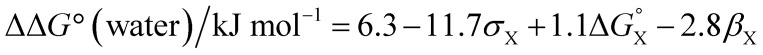
where *σ*_X_ is the Hammett constant for substituent X on the pyridine *N*-oxide guest, *β*_X_ is the H-bond acceptor parameter for the X functional group, and 
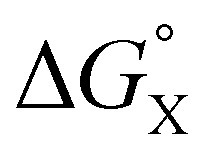
 is the free energy of transfer for PhX from water into *n*-hexadecane.

This analysis provides a rationalisation for the very large substituent effects observed in water. For example, addition of a single nitro substituent to the guest results in stabilisation of the complex formed with 1 in water by a factor of 2000. There are two different effects that contribute equally to this remarkable enhancement in binding affinity. The nitro group withdraws electron density from the aromatic ring of the guest, which leads to more favourable electrostatic interactions with π-electron density on the faces of the aromatic rings that line the walls of the binding pocket, stabilising the complex by a factor of 50. Partial desolvation of the nitro group on binding into the hydrophobic pocket in the receptor stabilises the complex by an additional factor of 40. The shape of the binding pocket allows the polar H-bond acceptor sites on the nitro group to maintain interactions with the solvent, while hydrophobic π-faces of the nitro group are desolvated on binding. As a result, the net contribution to binding associated with desolvation of the nitro substituent in water (−15 kJ mol^−1^) is similar to that observed for an isopropyl group (−16 kJ mol^−1^).

## Conclusions

Aromatic interactions play a major role in determining the three-dimensional structures and recognition properties of biomolecules, but the details of the relationship between chemical structure and the thermodynamic properties of these interactions has proved difficult to elucidate in aqueous solution. Aromatic interaction energies are strongly affected by the geometry of interaction, and the optimum interaction geometry is often not well-defined and can be changed by the presence of different substituents on the aromatic rings. Systematic investigation of the factors that govern the nature of aromatic interactions in water therefore requires a supramolecular system where the interaction geometry is controlled and the substituents on the interacting aromatic rings can be varied.

Here we show that calix[4]pyrrole complexes represent an excellent platform for quantitative experimental investigation of the properties of non-covalent interactions in water. The three-dimensional structure of the complexes formed with pyridine *N*-oxide guests are fixed by four H-bonding interactions between the pyrrole donors at the bottom of the receptor and the *N*-oxide acceptor on the guest. The host and guest are both relatively rigid molecules, so these H-bonding interactions also lock the geometrical arrangement of interacting functional groups at the top of the receptor binding pocket. In the system described here, an aromatic ring on the guest makes two edge-to-face interactions and two stacking interactions with the four aromatic side-walls of the cavity at the top of the calix[4]pyrrole receptor. Substituent effects on the free energy contribution due to these interactions were measured using chemical double mutant cycles (DMC) and a combination of ITC and NMR spectroscopy. In water, aromatic interactions between a phenyl group on the guest and the side-walls of the receptor increase the stability of the complex by three orders of magnitude compared with binding of a guest that lacks the phenyl group. Addition of a nitro substituent onto the guest phenyl group increases the stability of the complex by an additional three orders of magnitude, *i.e.* a factor 1 000 000 in total compared with binding of a guest that lacks the phenyl group. The resulting complex formed between the super-aryl-extended calix[4]pyrrole and 4-(4′-nitrophenyl)-pyridine *N*-oxide has a sub-picomolar dissociation constant in water (*K*_d_ = 370 fM).

Comparison of the DMC measurements in water with the same measurements made in chloroform solution allow dissection of the contributions due to aqueous solvation and the intrinsic properties of the aromatic interactions. Substituent effects on the free energy contributions associated with the aromatic interactions in chloroform correlate well with Hammett parameters: electron withdrawing groups on the guest aromatic ring stabilise the complex by enhancing the electrostatic interactions associated with both the edge-to-face and stacking interactions. For non-polar substituents, there is a substantial increase in the strength of the aromatic interactions in water, and the difference between water and chloroform correlates well with the hydrophobicity of the substituents, which can be measured using the free energy of transfer between water and *n*-hexadecane. For polar substituents, the complexes show unusually high stability, which suggests that these groups are not completely desolvated when the guest binds. Rather, the alkyl chains at the top of the receptor cavity rearrange to desolvate non-polar areas on the surface of these substituents but allow solvation of the polar H-bond acceptor sites by water. As a result, polar electron withdrawing substituents form remarkably stable complexes in water, with substituent effects that are orders of magnitude larger than the substituent effects observed in chloroform.

These experiments highlight the complexity of disentangling molecular recognition processes in water. The observed binding affinity results from a combination of the intrinsic properties of the functional group interactions and the extent to which the two binding partners are desolvated in the complex. Although functional groups interactions are straightforward to identify using spectroscopy or X-ray crystal structures, the effects of desolvation can be much more important in determining the free energy changes associated with complexation. Supramolecular complexes composed of relatively rigid components with well-defined three-dimensional structures that can be studied in both aqueous and organic solvents provide an opportunity to investigate the interplay of solvation and functional group interactions, which can be difficult to disentangle in more complicated and flexible biomolecular systems.

## Data availability

All supporting data is provided in the ESI.†

## Author contributions

The manuscript was written through contributions of all authors.

## Conflicts of interest

There are no conflicts to declare.

## Supplementary Material

SC-014-D3SC01027A-s001
