# Supplementary material for: Substituent effects on aromatic interactions in water
Source: Chem Sci. 2023 May 24;14(23):6226–36. doi: 10.1039/d3sc01027a (PMC10266462; doi:10.1039/d3sc01027a)
Supplement: SC-014-D3SC01027A-s001 [file SC-014-D3SC01027A-s001.pdf]

Electronic Supplementary Material (ESI) for Chemical Science.
This journal is © The Royal Society of Chemistry 2023

Substituent Effects on Aromatic Interactions in Water

Gloria Tobajas-Curiel,[1] Qingqing Sun,[2,3] Jeremy K. M. Sanders,[1] Pablo Ballester,[2,4,*] Christopher A. Hunter[1,*]

[1]*Yusuf Hamied Department of Chemistry, University of Cambridge, Cambridge CB2 1EW, U.K. Email: herchelsmith.orgchem@ch.cam.ac.uk*

[2] *Institute of Chemical Research of Catalonia (ICIQ), Barcelona Institute of Science and Technology (BIST), Av. Països Catalans, 16, 43007, Tarragona, Spain. Email: pballester@iciq.es*

[3]*Yangzhou University, School of Chemistry and Chemical Engineering, Yangzhou, 225002 Jiangsu (China). Email: sunqingqing@snnu.edu.cn.*

[4]*ICREA, Passeig Lluís Companys 23, 08010, Barcelona, Spain.*

Supporting Information

1. General methods 3

2. Chemistry procedures 4

2.1. General experimental procedure for the synthesis of 4-arylpyridine *N*-oxides 5-14 4

2.2. General experimental procedure for the synthesis of 4-arylpyridine *N*-oxides 15-17 4

2.3. Physical data of the 4-arylpyridine *N*-oxides 5-17 5

3. Isothermal titration calorimetry (ITC) experiments 31

3.1. Octapyridinium-super-aryl-extended calix[4]pyrrole 1 31

3.1.1. Complex C of the DMC 31

3.1.2. Complex A of the DMC 32

3.2. Tetrapyridinium-aryl-extended calix[4]pyrrole 3 39

3.2.1. Complex D of the DMC 39

3.2.2. Complex B of the DMC 40

3.3. Octachloro-super-aryl-extended calix[4]pyrrole 2 47

3.3.1. Complex C of the DMC 47

3.3.2. Complex A of the DMC 48

3.4. Tetrachloro-aryl-extened calix[4]pyrrole 4 55

3.4.1. Complex D of the DMC 55
3.4.2. Complex B of the DMC 56
4. Pairwise ^{1}H NMR competitive titrations 63
4.1. Octapyridinium-super-aryl-extended calix[4]pyrrole 1 63
4.2. Octachloro-super-aryl-extended calix[4]pyrrole 2 70
4.3. Tetrachloro-aryl-extended calix[4]pyrrole 4 77
5. Results of titration experiments 78
6. ^{1}H NMR analysis of the structures of complex A of the DMC 82
7. Analysis of the DMC results 84
8. References 88

1. General methods

All the reagents and materials used in the synthesis of the compounds described below were obtained from commercial sources and used without prior purification. Compounds **1-4** were prepared as reported in literature.[1–3] Thin layer chromatography was carried out using Silica gel 60F on glass plates. Flash chromatography was carried out on an automated system (Combiflash Companion, Combiflash Rf+ or Combiflash Rf Lumen) using prepacked cartridges of silica (25 µm or 50µm PuriFlash® Columns). ^1H and ^{13}C NMR spectra were recorded on Bruker 400 MHz DPX400, 400 MHz AVIII400, 500 MHz DCH cryoprobe or 500 MHz TCI Cryoprobe spectrometer at 298.0 ± 0.1 K unless specifically stated otherwise. Residual solvent was used as an internal standard for referencing. In chloroform-*d*, ^1H spectra were referenced to δ 7.26 ppm and ^{13}C spectra to δ 77.06 ppm for the solvent signal. In dimethyl sulfoxide-d_6, ^1H spectra were referenced to δ 2.50 ppm and ^{13}C spectra to δ 39.52 ppm. In deuterium oxide, ^1H spectra were referenced to δ 4.79 ppm. All chemical shifts are quoted in ppm on the δ scale and the coupling constants expressed in Hz. Signal splitting patterns are described as follows: s (singlet), br s (broad singlet), d (doublet), t (triplet), q (quartet), m (multiplet). FT-IR spectra were recorded on a PerkinElmer Spectrum One FT-IR spectrometer equipped with an ATR cell. The LCMS analysis of samples was performed using a Waters Acquity H-Class UPLC coupled with a single quadrupole Waters SQD2 or a Waters Xevo G2-S bench top QTOF machine. Melting points were measured on a Mettler Toledo MP90 melting point apparatus. ITC titrations were carried out on a Malvern MicroCal VP-ITC MicroCalorimeter.

2. Chemistry procedures

2.1. General experimental procedure for the synthesis of 4-arylpyridine *N*-oxides 5-14

To a solution of 4-chloro pyridine *N*-oxide (0.50 mmol, 1 equiv.) in dioxane (4 mL) under nitrogen, the corresponding aryl boronic acid (1.00 mmol, 2 equiv.), palladium tetrakis triphenylphospine (0.03 mmol, 0.05 equiv.) and sodium carbonate (2 M solution in water, 1 mL) were added. The mixture was stirred at 80 °C for 16 h. Ethyl acetate (10 mL) and water (5 mL) were then added. The black precipitate was filtered off through Celite® and the filtrate was extracted with ethyl acetate (3 x 10 mL). The combined organic layers were washed with brine, dried over anhydrous $MgSO_4$ and the solvents evaporated under reduced pressure. The crude product was purified by flash column chromatography on silica gel (methanol in dichloromethane 0 to 5 % in 20 minutes) to yield the corresponding 4-arylpyridine *N*-oxides **5-14**.

2.2. General experimental procedure for the synthesis of 4-arylpyridine *N*-oxides 15-17

To a solution of 4-chloro pyridine *N*-oxide (1.25 mmol, 5 equiv.) in dioxane (4 mL) under nitrogen, the corresponding aryl boronic acid (0.25 mmol, 1 equiv.), palladium tetrakis triphenylphospine (0.01 mmol, 0.05 equiv.) and sodium carbonate (2 M solution in water, 0.5 mL) were added. The mixture was stirred at 80 °C for 16 h. Ethyl acetate (10 mL) and water (5 mL) were then added. The black precipitate was filtered off through Celite® and the filtrate was extracted with ethyl acetate (3 x 10 mL). The combined organic layers were washed with brine, dried over anhydrous $MgSO_4$ and the solvents evaporated under reduced pressure. The crude product was purified by reverse phase flash column chromatography on C-18 (acetonitrile in water 10 to 50 % in 20 minutes) to yield the corresponding 4-halophenyl pyridine *N*-oxides **15-17**.

2.3. Physical data of the 4-arylpyridine *N*-oxides 5-17

4-Phenylpyridine 1-oxide (5)

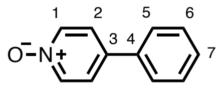

Off white solid (59 mg, 0.34 mmol, 69 % yield)

M.p.: 151 – 153 °C.

^{1}H NMR (400 MHz, DMSO-*d*$_6$): δ_H = 8.26 (d, *J* = 7.1 Hz, 2H, H-1), 7.79 – 7.76 (m, 4H, H-2, H-5), 7.50 (t, *J* = 7.5 Hz, 2H, H-6), 7.45 – 7.41 (m, 1H, H-7).

^{13}C NMR (101 MHz, DMSO-*d*$_6$): δ_C = 138.9 (C-1), 136.0 (C-3), 135.6 (C-4), 129.2 (C-6), 128.8 (C-7), 126.2 (C-5), 123.6 (C-2).

HRMS (ES+): calculated for $C_{11}H_{10}NO$ 172.0757 [M+H$^+$], found 172.0755 [M+H$^+$].

FT-IR (ATR): ν_{max} 3335, 3109, 1687, 1472, 1431, 1230, 1183, 843, 779, 576 cm^{-1}.

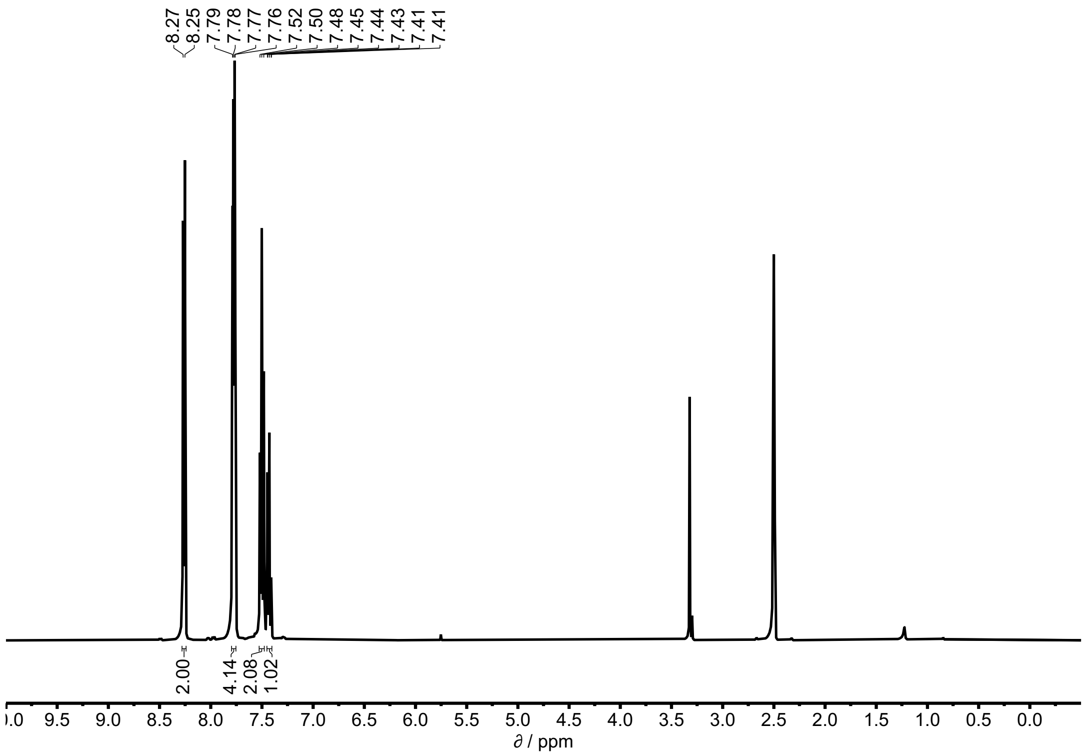

Figure S1. *^{1}H NMR spectrum (400 MHz, DMSO-d$_6$) of compound* ***5****.*

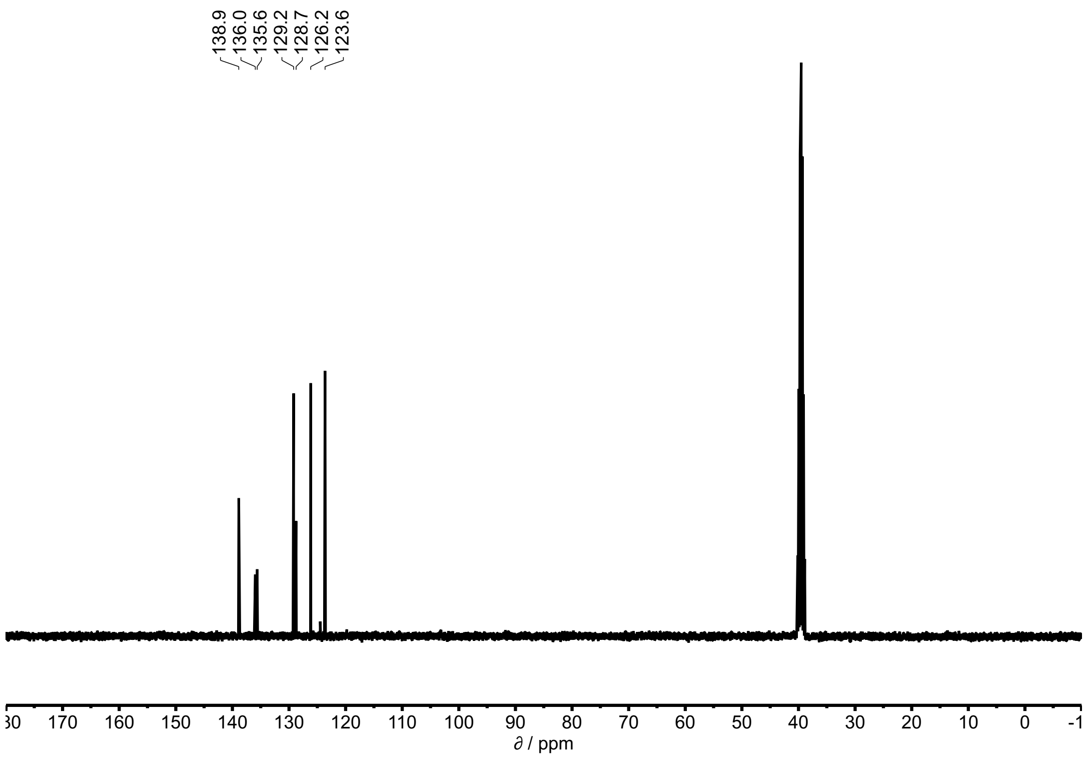

Figure S2. *^{13}C NMR spectrum (101 MHz, DMSO-d$_6$) of compound* ***5****.*

4-(*p*-Tolyl)pyridine 1-oxide (6)

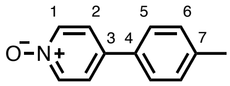

Pink solid (73 mg, 0.39 mmol, 79 % yield)

M.p.: 136 – 138 °C.

^{1}H NMR (400 MHz, DMSO-*d*$_6$): δ_H = 8.23 (d, J = 7.1 Hz, 2H, H-1), 7.74 (d, J = 7.1 Hz, 2H, H-2), 7.67 (d, J = 8.0 Hz, 2H, H-5), 7.30 (d, J = 8.0 Hz, 2H, H-6), 2.34 (s, 3H, CH_3).

^{13}C NMR (101 MHz, DMSO-*d*$_6$): δ_C = 138.8 (C-1), 138.4 (C-7), 136.0 (C-3), 132.7 (C-4), 129.8 (C-6), 126.0 (C-5), 123.3 (C-2), 20.7 (CH_3).

HRMS (ES+): calculated for $C_{12}H_{12}NO$ 186.0913 [M+H$^+$], found 186.0909 [M+H$^+$].

FT-IR (ATR): ν_{max} 3506, 3034, 1480, 1243, 1181, 1027, 815, 679, 532 cm^{-1}.

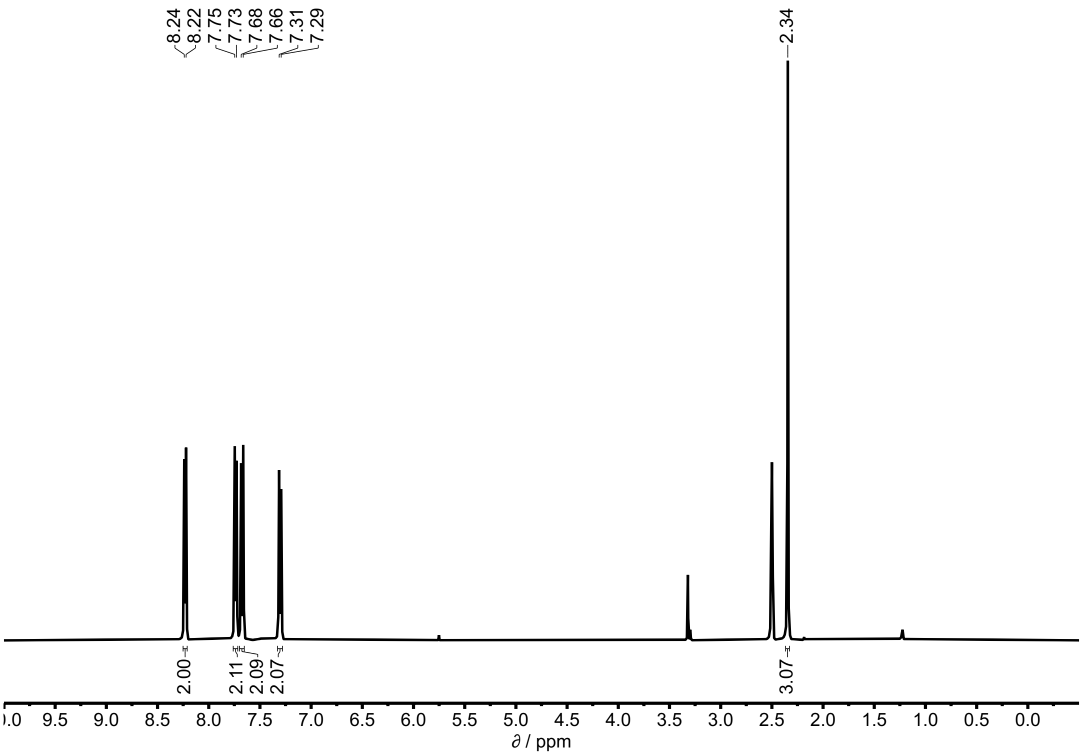

Figure S3. *^{1}H NMR spectrum (400 MHz, DMSO-d6) of compound **6**.*

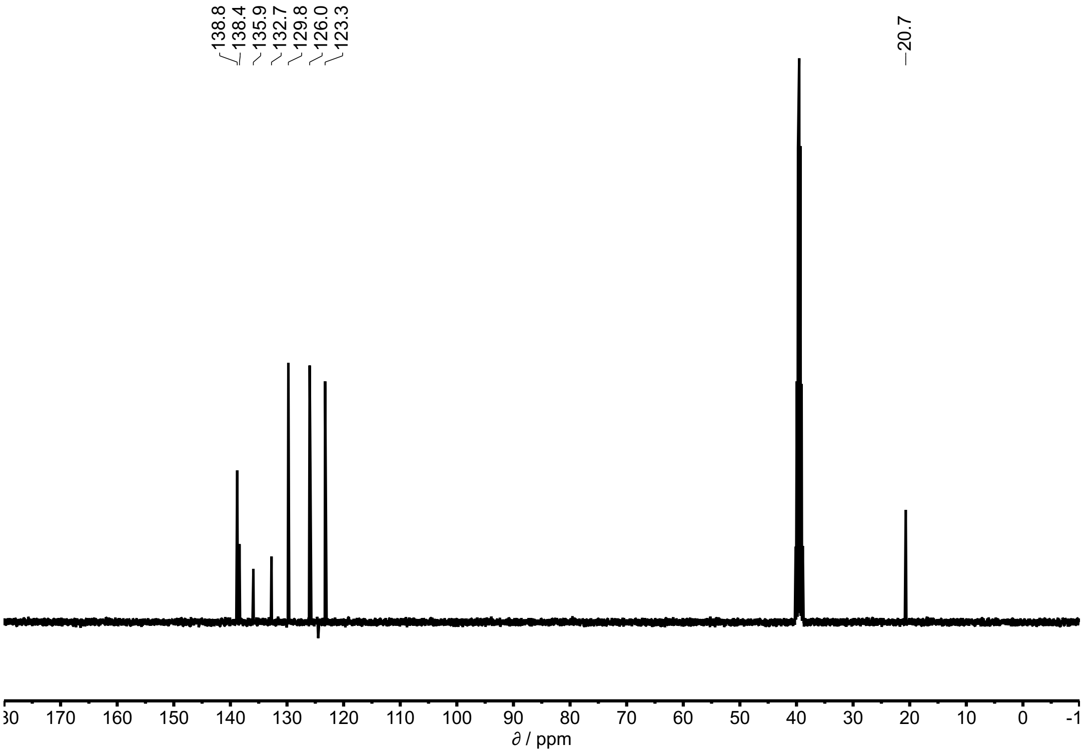

Figure S4. *^{13}C NMR spectrum (101 MHz, DMSO-d6) of compound **6**.*

4-(4-(Dimethylamino)phenyl)pyridine 1-oxide (7)

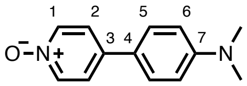

Brown solid (78 mg, 0.36 mmol, 73 % yield)

M.p.: 242 – 244 °C.

^{1}H NMR (400 MHz, DMSO-*d*$_{6}$): δ_H = 8.14 (d, *J* = 7.1 Hz, 2H, H-1), 7.67 – 7.62 (m, 4H, H-2, H-5), 6.79 (d, *J* = 8.9 Hz, 2H, H-6), 2.96 (s, 6H, $N(CH_3)_2$).

^{13}C NMR (101 MHz, DMSO-*d*$_{6}$): δ_C = 150.6 (C-7), 138.6 (C-1), 136.6 (C-3), 126.8 (C-5), 122.4 (C-4), 121.8 (C-2), 112.4 (C-6), 39.8 ($N(CH_3)_2$).

HRMS (ES+): calculated for $C_{13}H_{15}N_2O$ 215.1179 [M+H$^+$], found 215.1173 [M+H$^+$].

FT-IR (ATR): ν_{max} 2899, 1604, 1471, 1365, 1252, 1183, 1028, 813, 660 cm^{-1}.

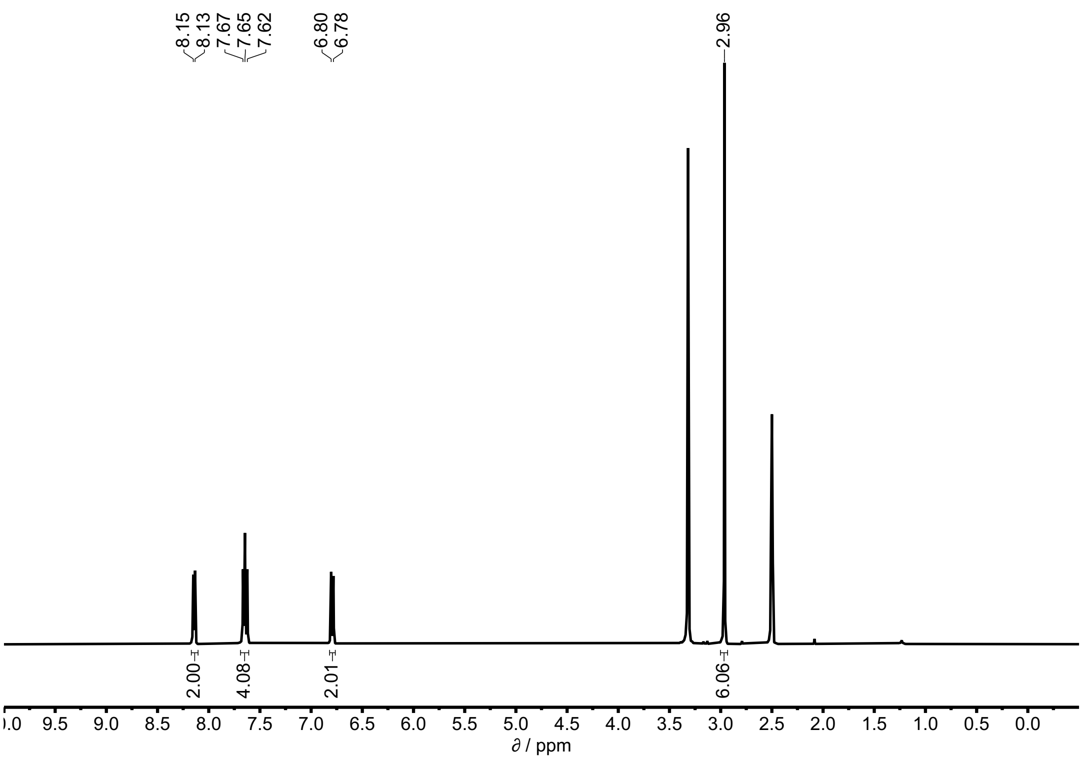

Figure S5. *^{1}H NMR spectrum (400 MHz, DMSO-d_6) of compound* ***7****.*

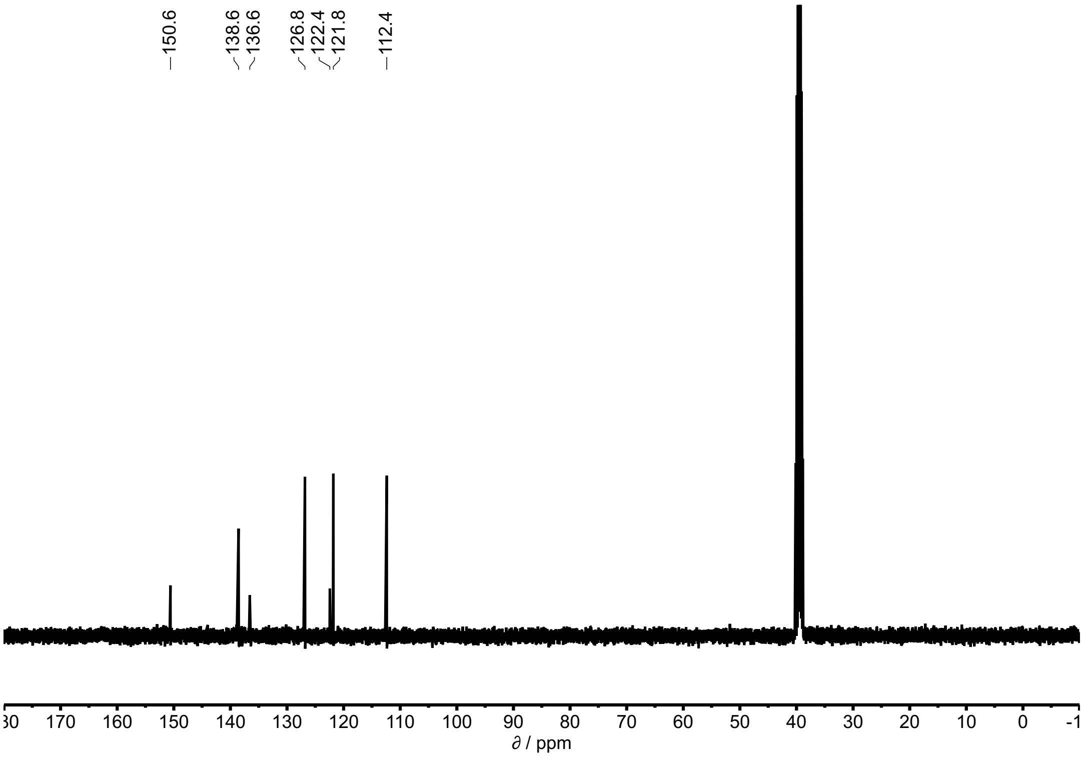

Figure S6. *^{13}C NMR spectrum (101 MHz, DMSO-d_6) of compound* ***7****.*

4-(4-(Trifluoromethyl)phenyl)pyridine 1-oxide (8)

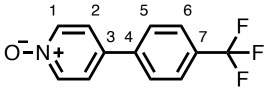

Off-white solid (84 mg, 0.35 mmol, 70 % yield)

M.p.: 177 – 179 °C.

^{1}H NMR (400 MHz, DMSO-*d*$_6$): δ_H = 8.32 (d, *J* = 7.1 Hz, 2H, H-1), 8.01 (d, *J* = 8.2 Hz, 2H, H-5), 7.87 – 7.84 (m, 4H, H-2, H-6).

^{13}C NMR (101 MHz, DMSO-*d*$_6$): δ_C = 139.7 (CF_3), 139.1 (C-1), 134.2 (C-3), 127.1 (C-5), 126.0 (C-6), 124.5 (C-4), 124.2 (C-2).

HRMS (ES+): calculated for $C_{12}H_9F_3NO$ 240.0631 [M+H$^+$], found 240.0625 [M+H$^+$].

FT-IR (ATR): ν_{max} 3436, 3115, 1615, 1483, 1328, 1115, 1074, 850, 717 cm^{-1}.

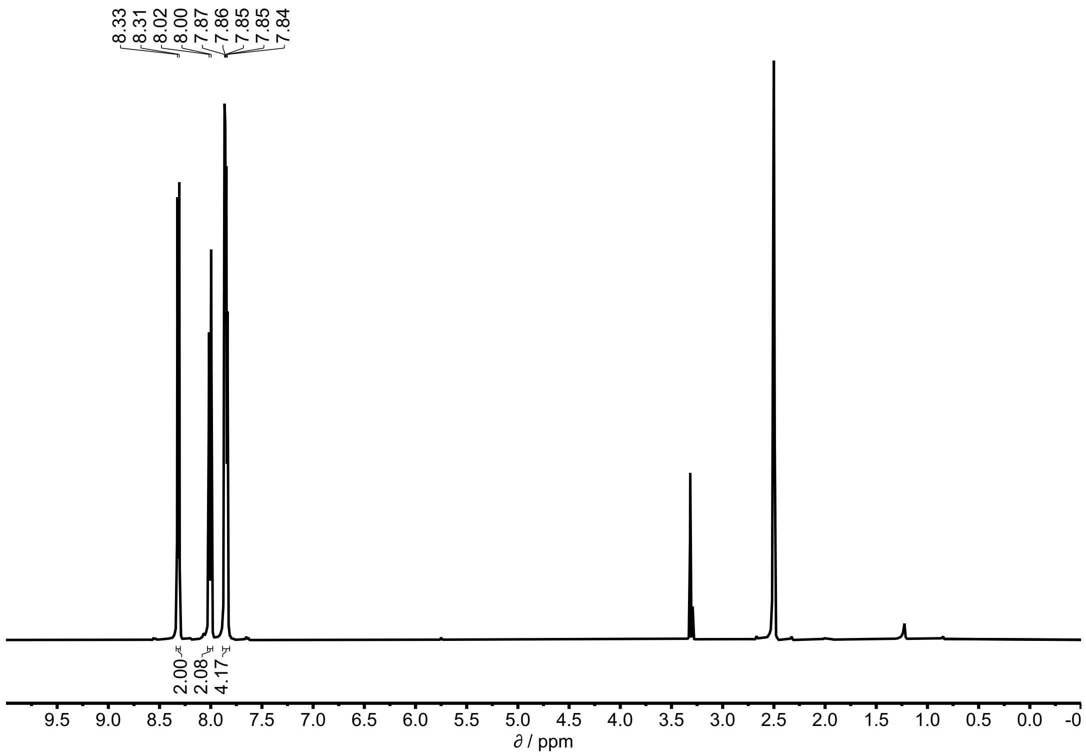

Figure S7. *^{1}H NMR spectrum (400 MHz, DMSO-d$_6$) of compound **8**.*

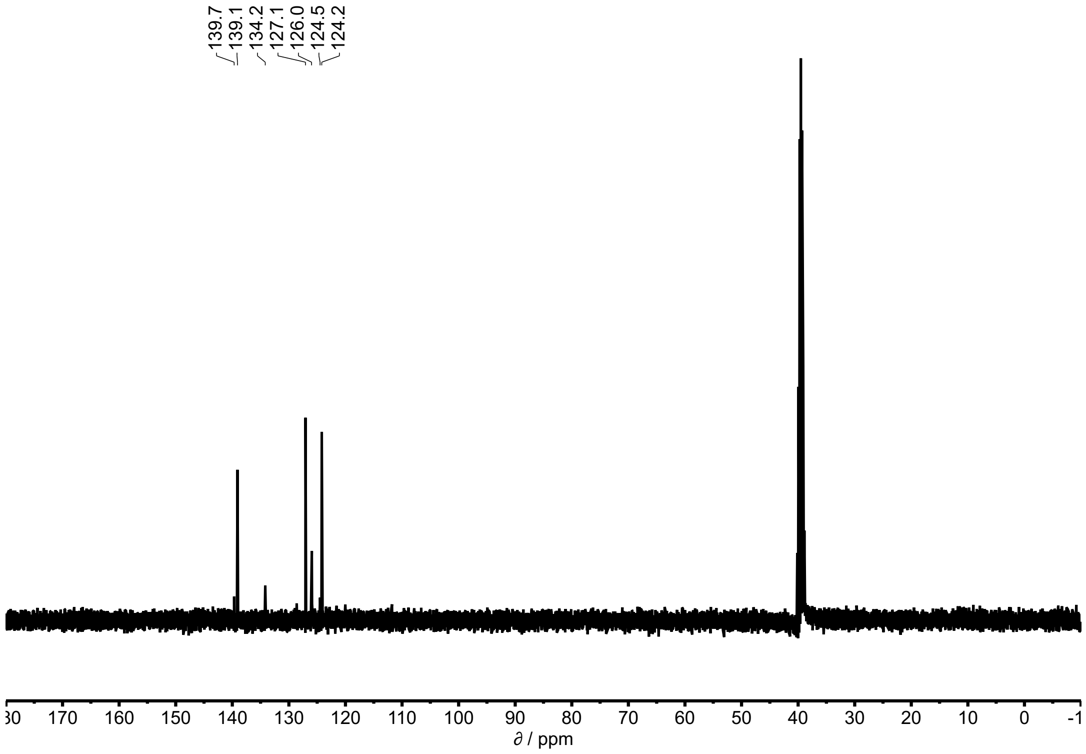

Figure S8. *^{13}C NMR spectrum (101 MHz, DMSO-d$_6$) of compound **8**.*

4-(4-Nitrophenyl)pyridine 1-oxide (9)

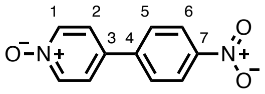

Yellow solid (82 mg, 0.38 mmol, 75 % yield)

M.p.: 218 – 200 °C.

^{1}H NMR (400 MHz, DMSO-*d*$_6$): δ_H = 8.34 – 8.30 (m, 4H, H-1, H-6), 8.07 (d, *J* = 8.9 Hz, 2H, H-5), 7.90 (d, *J* = 7.1 Hz, 2H, H-2).

^{13}C NMR (101 MHz, DMSO-*d*$_6$): δ_C = 147.2 (C-7), 141.9 (C-3), 139.2 (C-1), 133.4 (C-4), 127.5 (C-5), 124.4 (C-2), 124.2 (C-6).

HRMS (ES+): calculated for $C_{11}H_9N_2O_3$ 217.0608 [M+H$^+$], found 217.0603 [M+H$^+$].

FT-IR (ATR): ν_{max} 1600, 1511, 1475, 1345, 1254, 1201, 835, 755 cm-1.

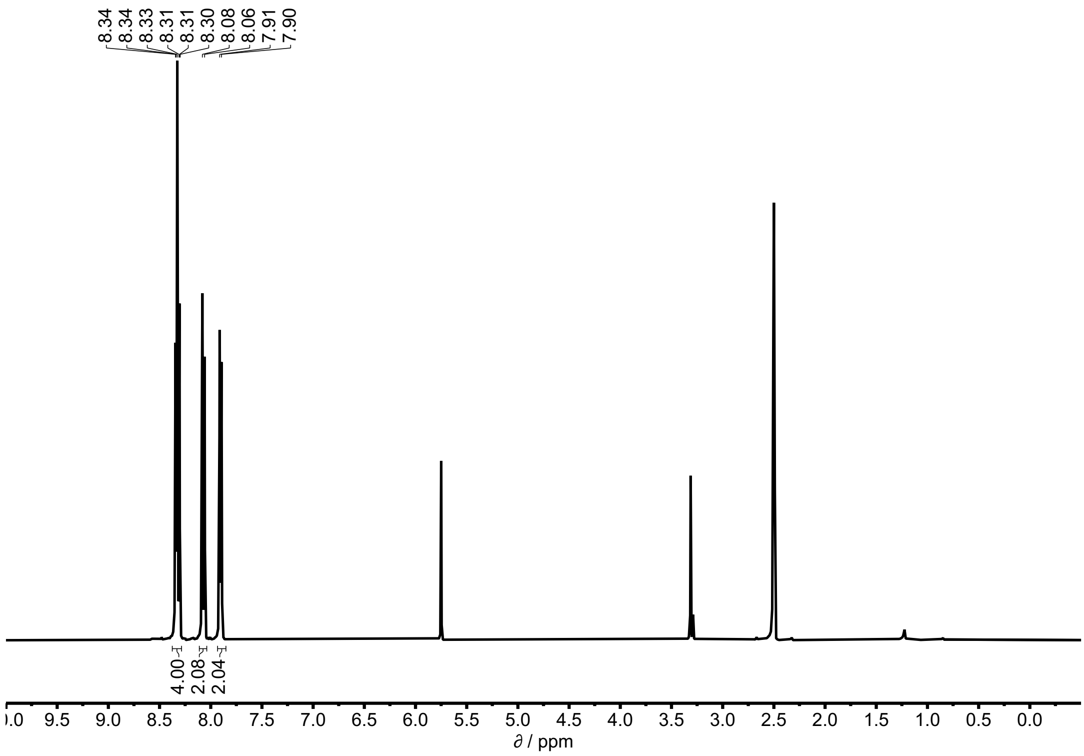

Figure S9. *^{1}H NMR spectrum (400 MHz, DMSO-d$_6$) of compound **9**.*

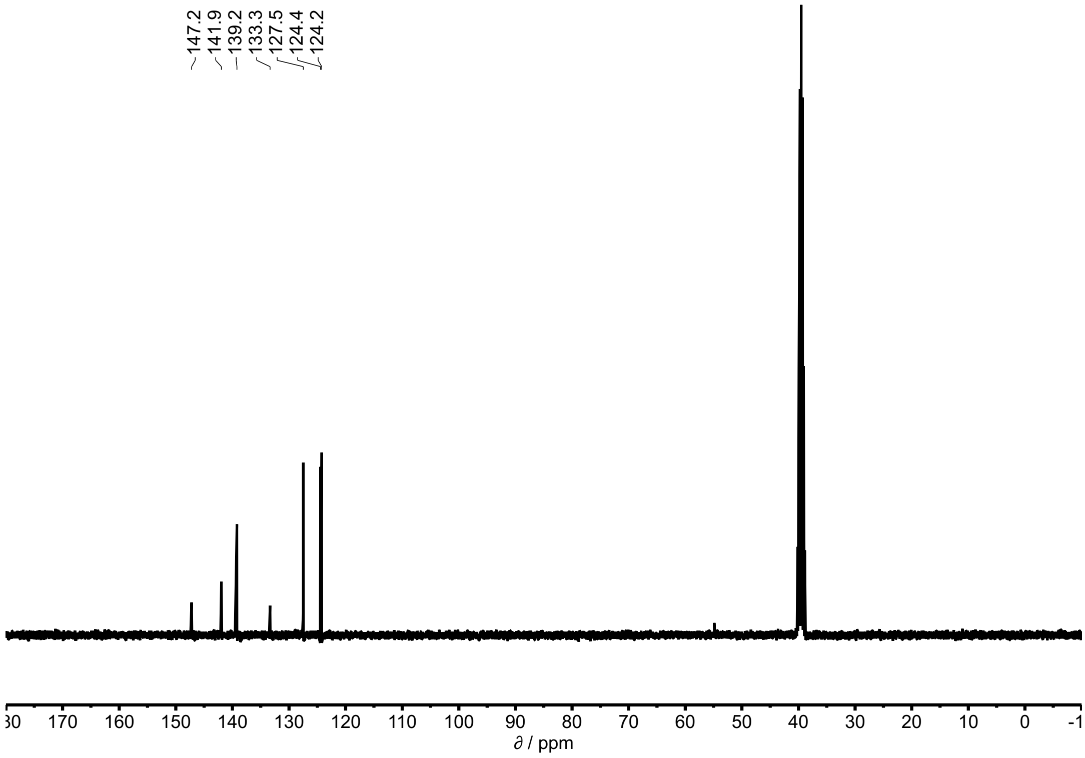

Figure S10. *^{13}C NMR spectrum (101 MHz, DMSO-d$_6$) of compound **9**.*

4-(4-Methoxyphenyl)pyridine 1-oxide (10)

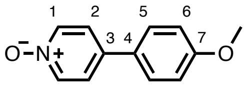

Off-white solid (67 mg, 0.33 mmol, 66 % yield)

M.p.: 113 – 116 °C.

^{1}H NMR (400 MHz, DMSO-*d*$_6$): δ_H = 8.21 (d, *J* = 7.2 Hz, 2H, H-1), 7.75 – 7.70 (m, 4H, H-2, H-5), 7.05 (d, *J* = 8.8 Hz, 2H, H-6), 3.81 (s, 3H, OCH_3).

^{13}C NMR (101 MHz, DMSO-*d*$_6$): δ_C = 159.9 (C-7), 138.8 (C-1), 135.8 (C-3), 127.9 (C-4), 127.5 (C-5), 122.9 (C-2), 114.6 (C-6), 55.3 (OCH_3).

HRMS (ES+): calculated for $C_{12}H_{12}NO_2$ 202.0868 [M+H$^+$], found 202.0874 [M+H$^+$].

FT-IR (ATR): ν_{max} 3380, 1607, 1478, 1289, 1231, 1177, 1036, 826, 800, 552 cm^{-1}.

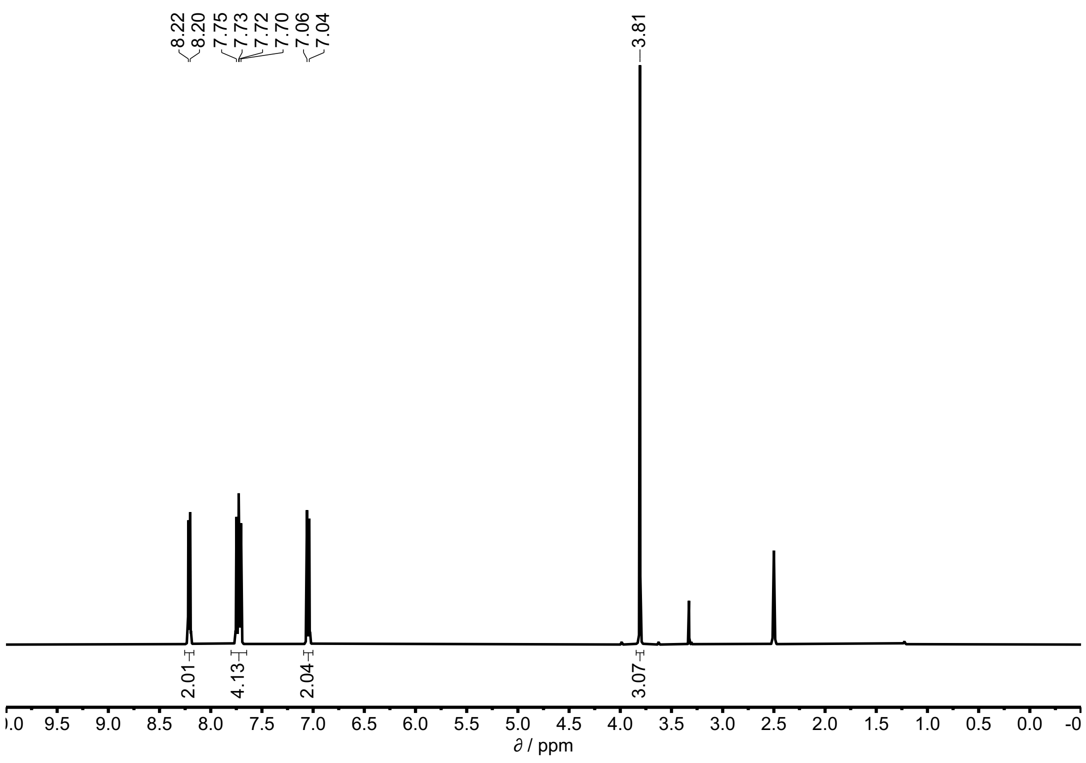

Figure S11. *^{1}H NMR spectrum (400 MHz, DMSO-d6) of compound* ***10****.*

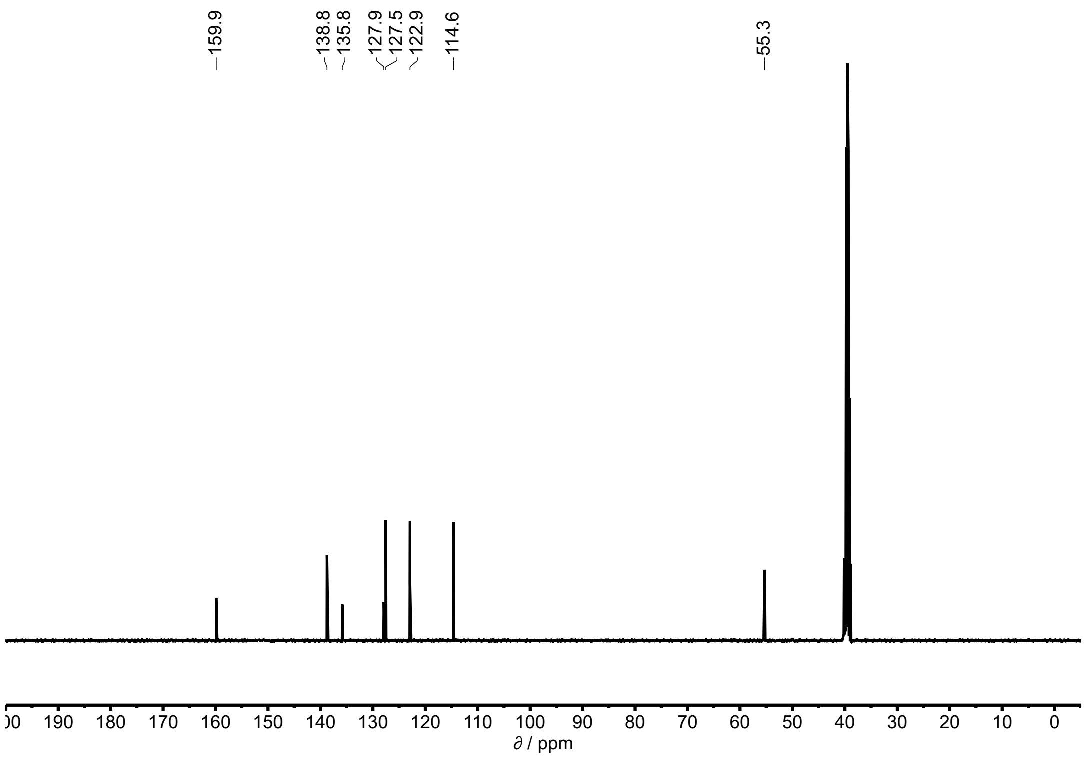

Figure S12. *^{13}C NMR spectrum (101 MHz, DMSO-d6) of compound* ***10****.*

4-(4-Formylphenyl)pyridine 1-oxide (11)

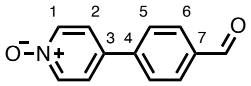

White solid (65 mg, 0.33 mmol, 65 % yield)

M.p.: 185 – 187 °C.

^{1}H NMR (400 MHz, DMSO-*d*$_6$): δ_H = 10.06 (s, 1H, CHO), 8.32 (d, *J* = 5.8 Hz, 2H, H-1), 8.02 (s, 4H, H-6, H-5), 7.88 (d, *J* = 5.8 Hz, 2H, H-2).

^{13}C NMR (101 MHz, DMSO-*d*$_6$): δ_C = 192.7 (C=O), 141.1 (C-4), 139.1 (C-1), 135.8 (C-7), 134.4 (C-3), 130.2 (C-6), 126.9 (C-5), 124.2 (C-2).

HRMS (ES+): calculated for $C_{12}H_{10}NO_2$ 200.0712 [M+H$^+$], found 200.0717 [M+H$^+$].

FT-IR (ATR): ν_{max} 3326, 1697, 1605, 1479, 1252, 1184, 856, 831, 818, 714 cm^{-1}.

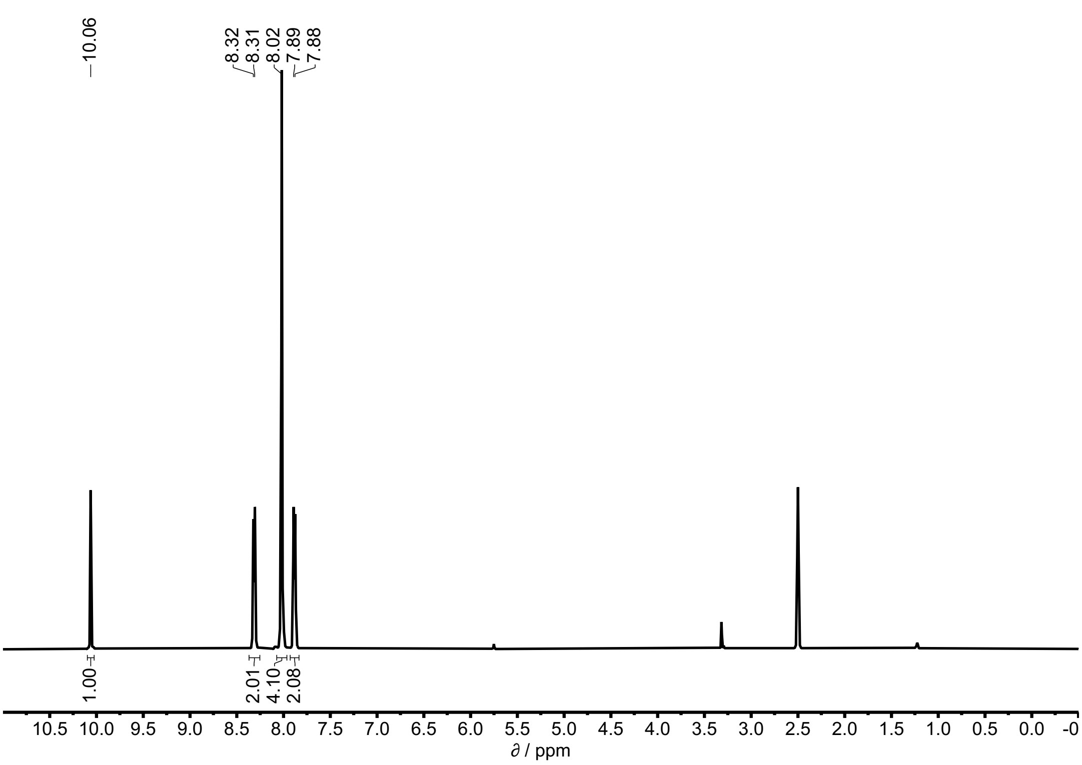

Figure S13. *¹H NMR spectrum (400 MHz, DMSO-d_6) of compound **11**.*

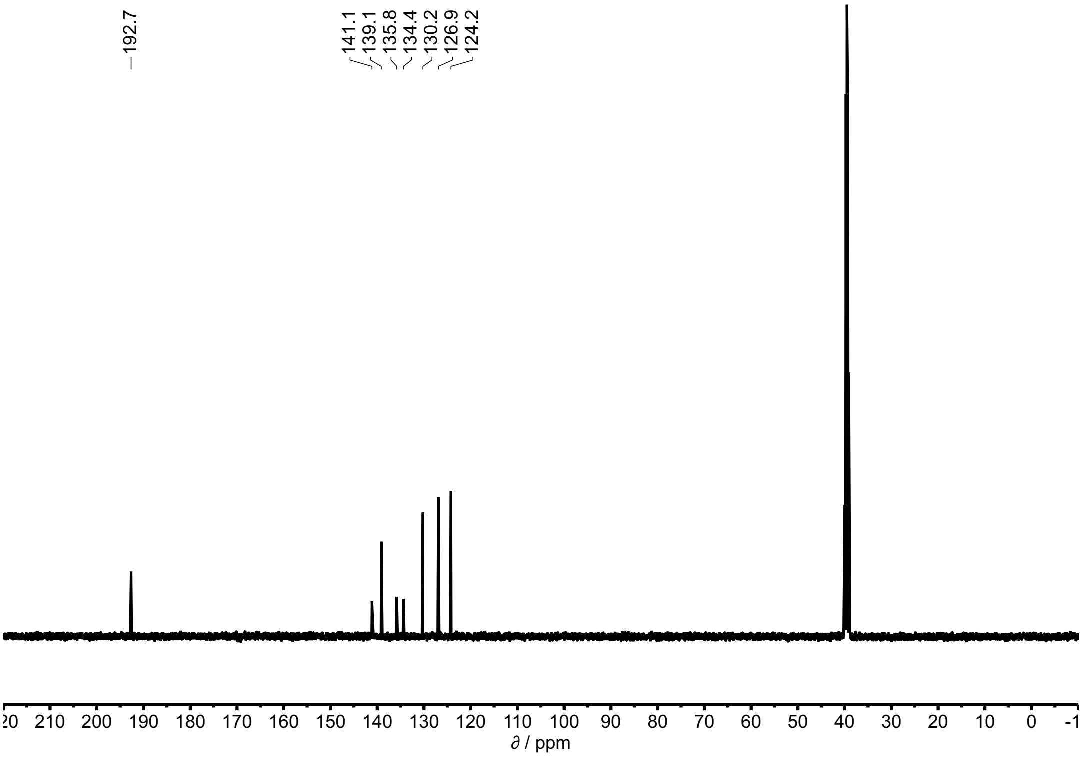

Figure S14. *¹³C NMR spectrum (101 MHz, DMSO-d_6) of compound **11**.*

4-(4-Acetylphenyl)pyridine 1-oxide (12)

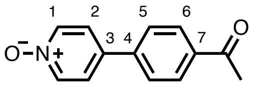

Off-white solid (85 mg, 0.40 mmol, 79 % yield)

M.p.: 161 – 163 °C.

^{1}H NMR (400 MHz, DMSO-*d*$_6$): δ_H = 8.30 (d, *J* = 7.4 Hz, 2H, H-1), 8.05 (d, *J* = 8.7 Hz, 2H, H-6), 7.94 (d, *J* = 8.7 Hz, 2H, H-5), 7.87 (d, *J* = 7.4 Hz, 2H, H-2), 2.62 (s, 3H, $COCH_3$).

^{13}C NMR (101 MHz, DMSO-*d*$_6$): δ_C = 197.4 (C=O), 139.8 (C-4), 139.1 (C-1), 136.5 (C-7), 134.5 (C-3), 129.0 (C-6), 126.4 (C-5), 124.1 (C-2), 26.8 ($COCH_3$).

HRMS (ES+): calculated for $C_{13}H_{12}NO_2$ 214.0868 [M+H$^+$], found 214.0872 [M+H$^+$].

FT-IR (ATR): ν_{max} 3335, 1688, 1605, 1480, 1268, 1245, 1196, 850, 820, 657 cm^{-1}.

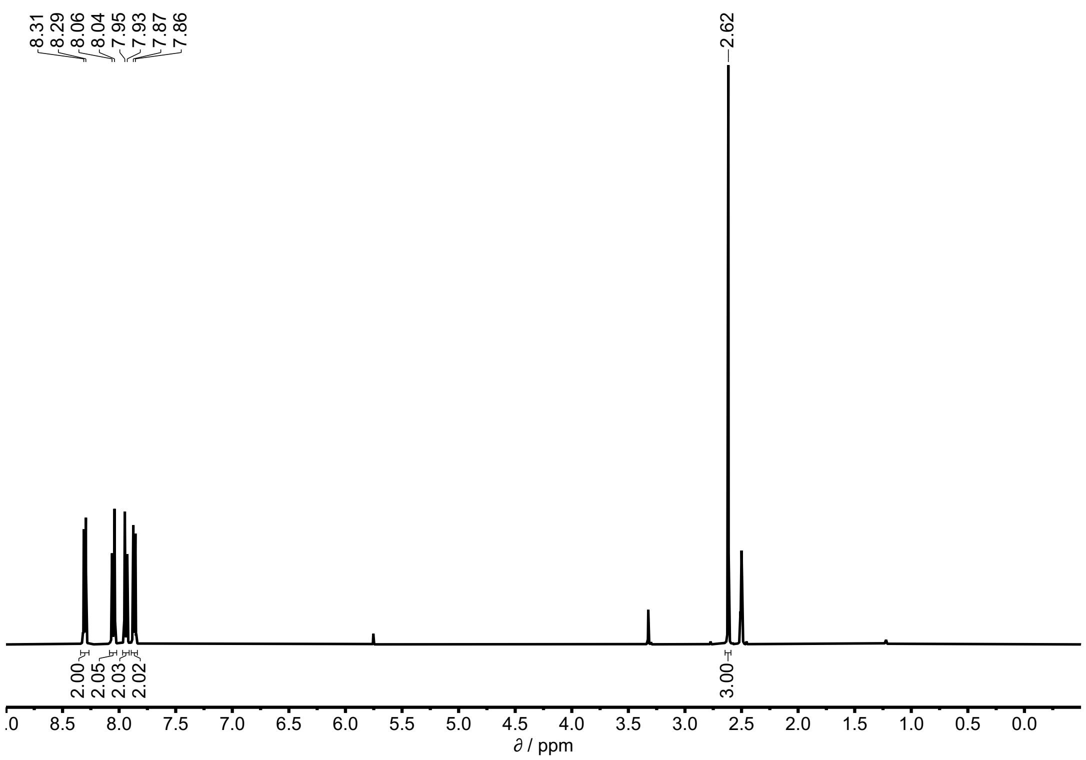

Figure S15. *¹H NMR spectrum (400 MHz, DMSO-d₆) of compound* ***12****.*

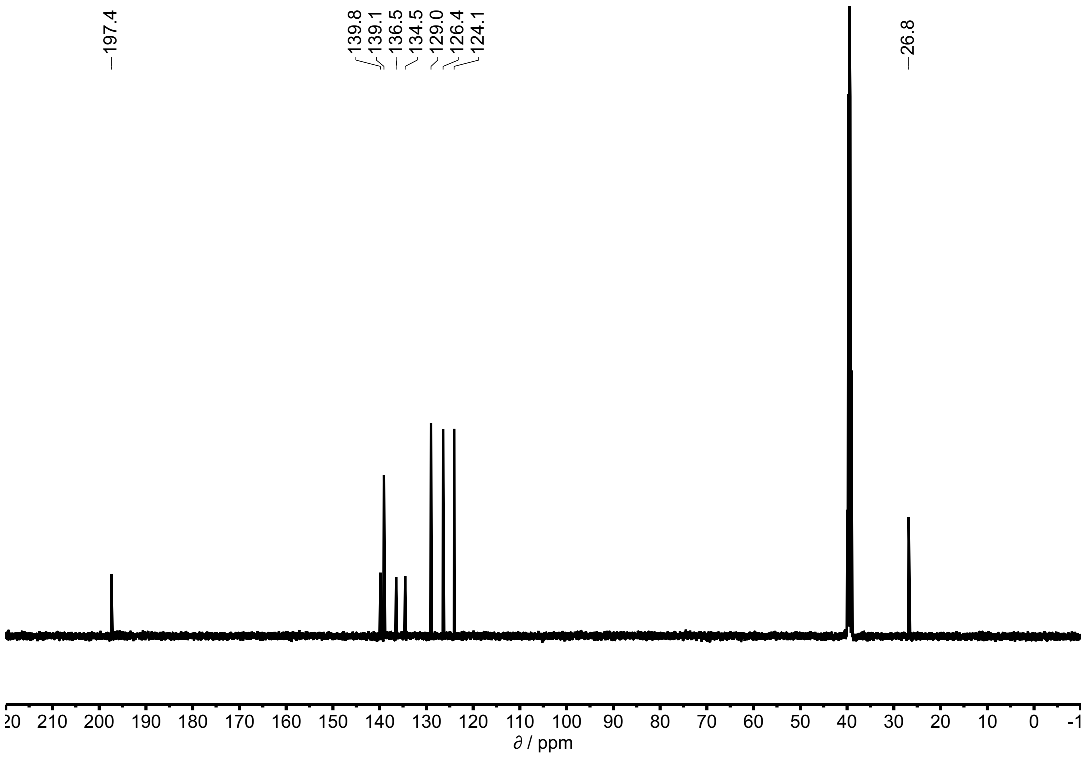

Figure S16. *¹³C NMR spectrum (101 MHz, DMSO-d₆) of compound* ***12****.*

4-(4-Isopropylphenyl)pyridine 1-oxide (13)

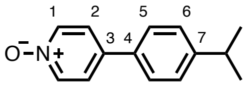

Pink solid (74 mg, 0.35 mmol, 69 % yield)

M.p.: 143 – 145 °C.

^{1}H NMR (400 MHz, DMSO-*d*$_6$): δ_H = 8.24 (d, *J* = 7.3 Hz, 2H, H-1), 7.74 (d, *J* = 7.3 Hz, 2H, H-2), 7.69 (d, *J* = 8.5 Hz, 2H, H-5), 7.36 (d, *J* = 8.2 Hz, 2H, H-6), 2.93 (p, *J* = 6.9 Hz, 1H, CH), 1.22 (d, J = 6.9 Hz, 6H, CH_3).

^{13}C NMR (101 MHz, DMSO-*d*$_6$): δ_C = 149.2 (C-7), 138.8 (C-1), 136.1 (C-3), 133.2 (C-4), 127.2 (C-6), 126.2 (C-5), 123.4 (C-2), 33.1 (CH), 23.7 (CH_3).

HRMS (ES+): calculated for $C_{14}H_{16}NO$ 214.1232 [M+H$^+$], found 214.1240 [M+H$^+$].

FT-IR (ATR): ν_{max} 3360, 2959,1476, 1409, 1237, 1179, 1025, 847, 823, 770, 713, 665 cm^{-1}.

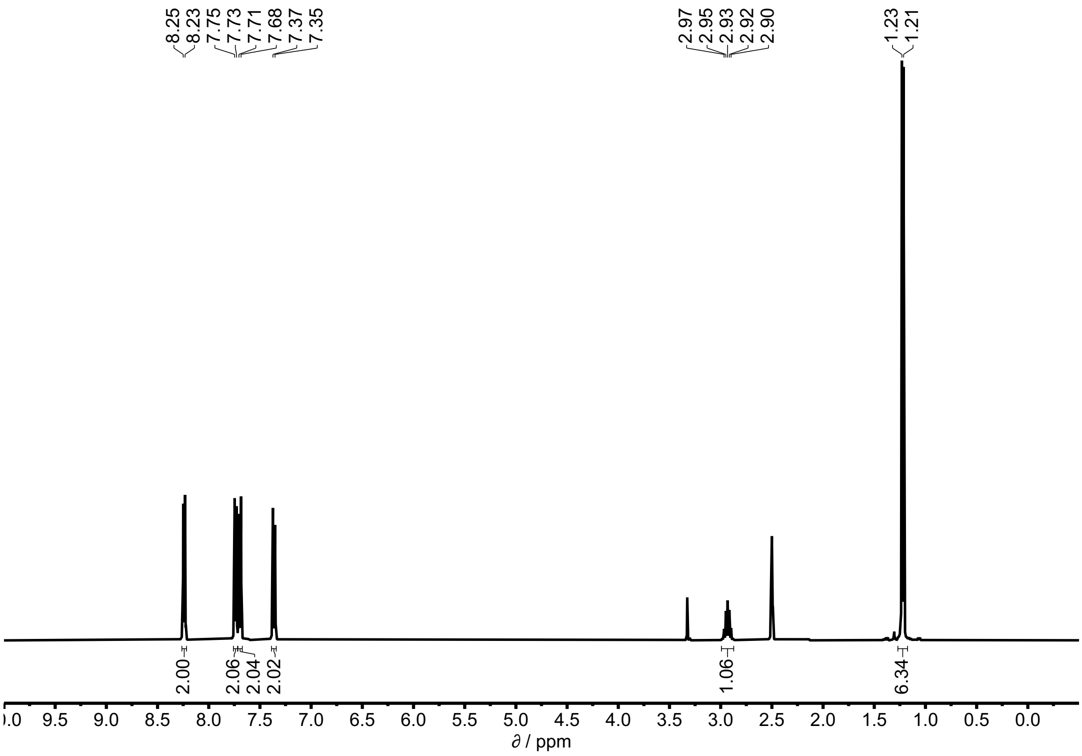

Figure S17. *¹H NMR spectrum (400 MHz, DMSO-d₆) of compound* ***13****.*

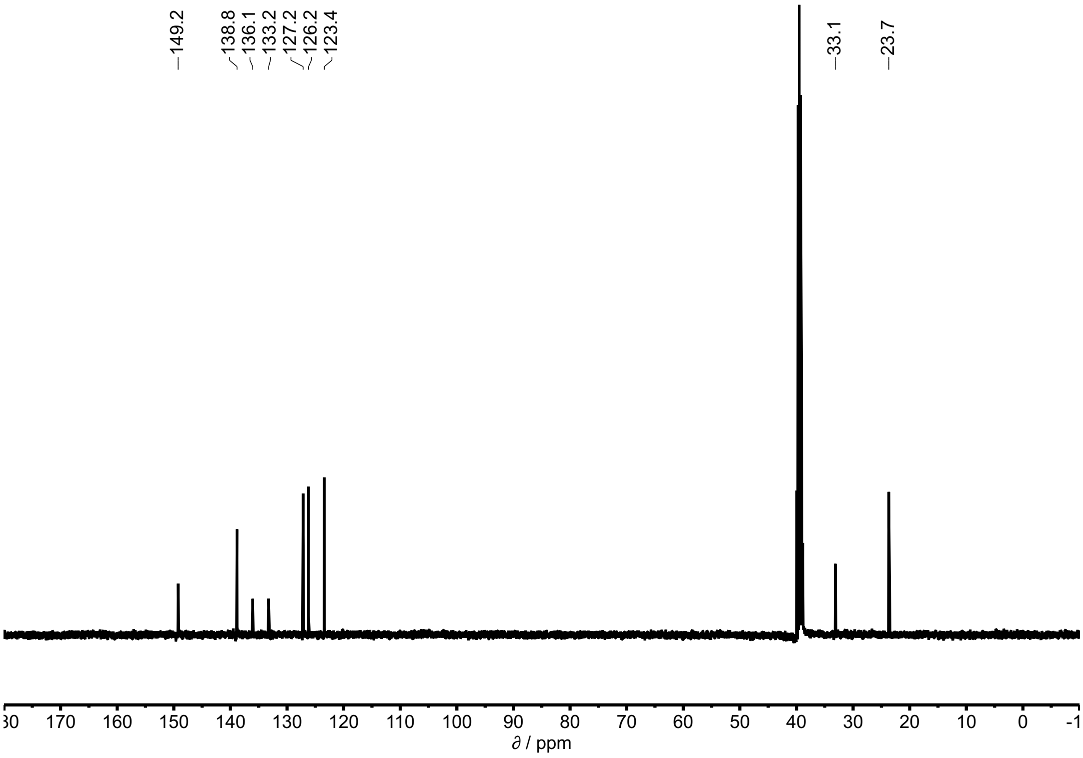

Figure S18. *¹³C NMR spectrum (101 MHz, DMSO-d₆) of compound* ***13****.*

4-(4-Ethylphenyl)pyridine 1-oxide (14)

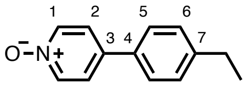

Brown solid (73 mg, 0.37 mmol, 73 % yield)

M.p.: 116 – 118 °C.

^{1}H NMR (400 MHz, DMSO-*d*$_6$): δ_H = 8.24 (d, *J* = 5.6 Hz, 2H, H-1), 7.74 (d, *J* = 5.6 Hz, 2H, H-2), 7.69 (d, *J* = 6.7 Hz, 2H, H-5), 7.33 (d, *J* = 7.0 Hz, 2H, H-6), 2.65 (q, *J* = 7.6 Hz, 2H, CH_2), 1.20 (t, *J* = 7.6 Hz, 3H, CH_3).

^{13}C NMR (101 MHz, DMSO-*d*$_6$): δ_C = 144.7 (C-7), 138.8 (C-1), 136.0 (C-3), 133.1 (C-4), 128.6 (C-6), 126.1 (C-5), 123.4 (C-2), 27.8 (CH_2), 15.4 (CH_3).

HRMS (ES+): calculated for $C_{13}H_{14}NO$ 200.1075 [M+H$^+$], found 200.1081 [M+H$^+$].

FT-IR (ATR): ν_{max} 3379, 2964, 1474, 1450, 1408, 1234, 1176, 1029, 848, 822, 784, 711, 672, 637 cm^{-1}.

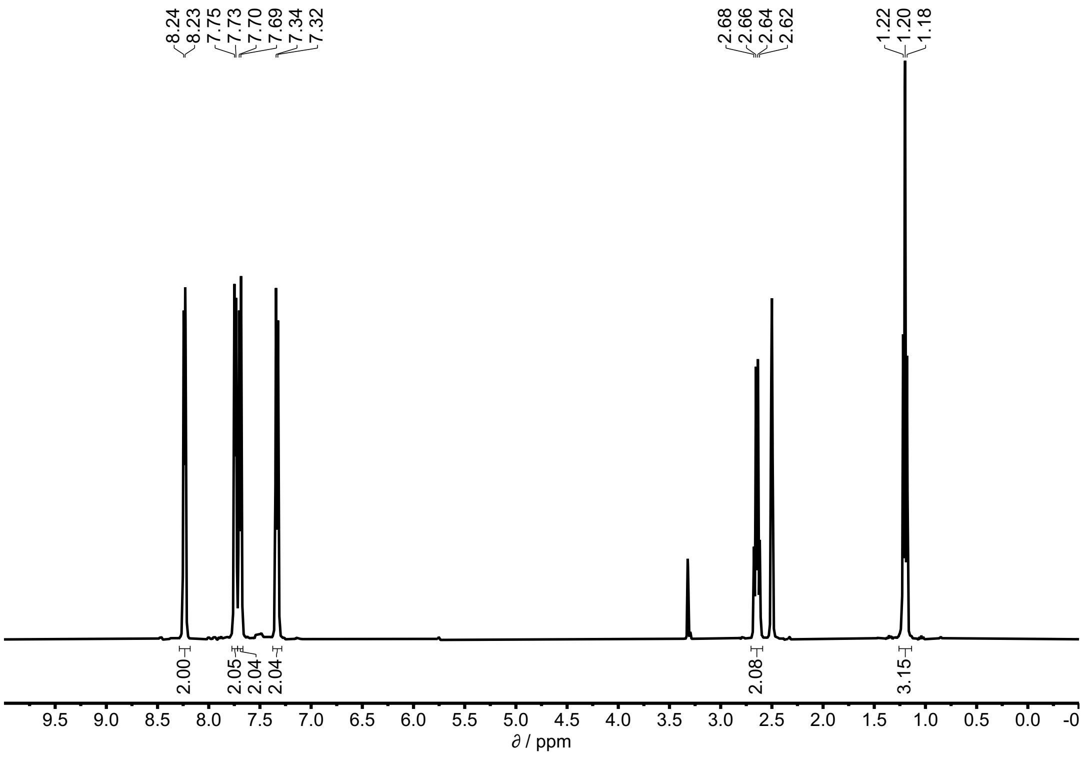

Figure S19. *^{1}H NMR spectrum (400 MHz, DMSO-d_6) of compound* ***14.***

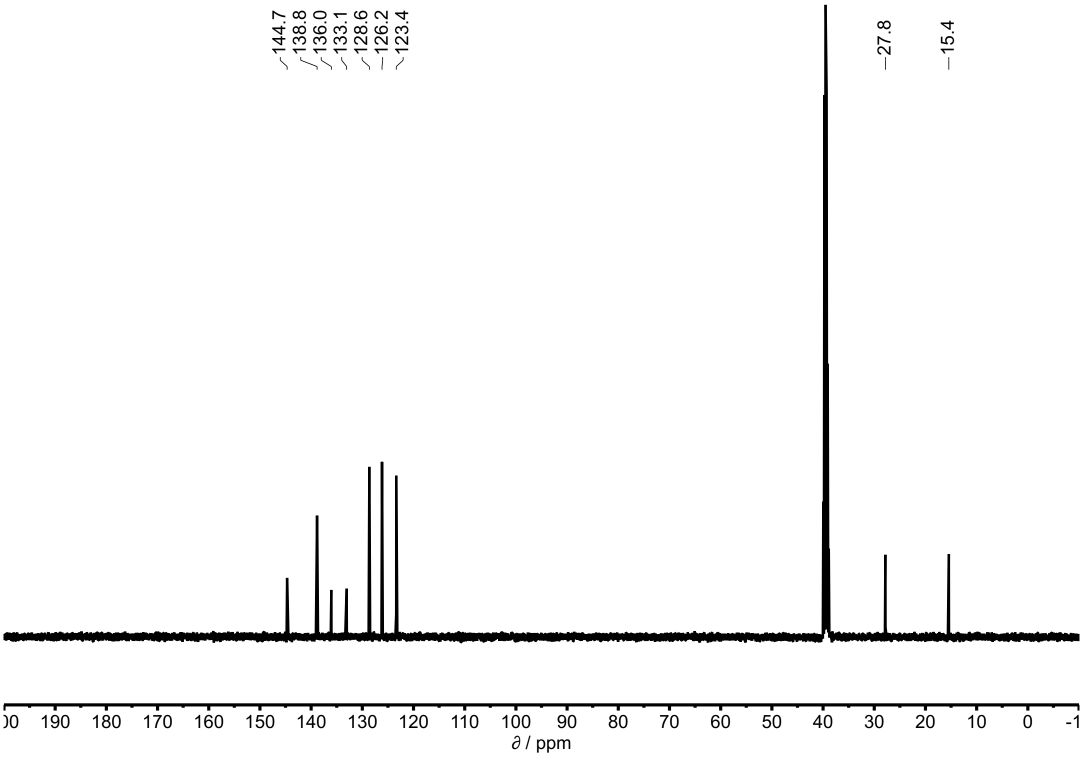

Figure S20. *^{13}C NMR spectrum (101 MHz, DMSO-d_6) of compound* ***14.***

4-(4-Fluorophenyl)pyridine 1-oxide (15)

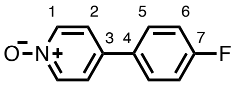

White solid (13 mg, 0.07 mmol, 14 % yield)

M.p.: 157 – 159 °C.

^{1}H NMR (400 MHz, DMSO-*d*$_6$): δ_H = 8.26 (d, *J* = 5.3 Hz, 2H, H-1), 7.84 (t, *J* = 6.3 Hz, 2H, H-5), 7.76 (d, *J* = 5.5 Hz, 2H, H-2), 7.33 (t, *J* = 8.8 Hz, 2H, H-6).

^{13}C NMR (101 MHz, DMSO-*d*$_6$): δ_C = 163.7 (C-7), 138.8 (C-1), 134.9 (C-3), 132.1 (C-4), 128.4 (C-5), 123.6 (C-2), 116.1 (C-6).

HRMS (ES+): calculated for $C_{11}H_9FNO$ 190.0668 [M+H$^+$], found 190.0673 [M+H$^+$].

FT-IR (ATR): ν_{max} 3112, 3039, 1600, 1518, 1475, 1450, 1405, 1247, 1224, 1179, 1162, 1024, 814 cm^{-1}.

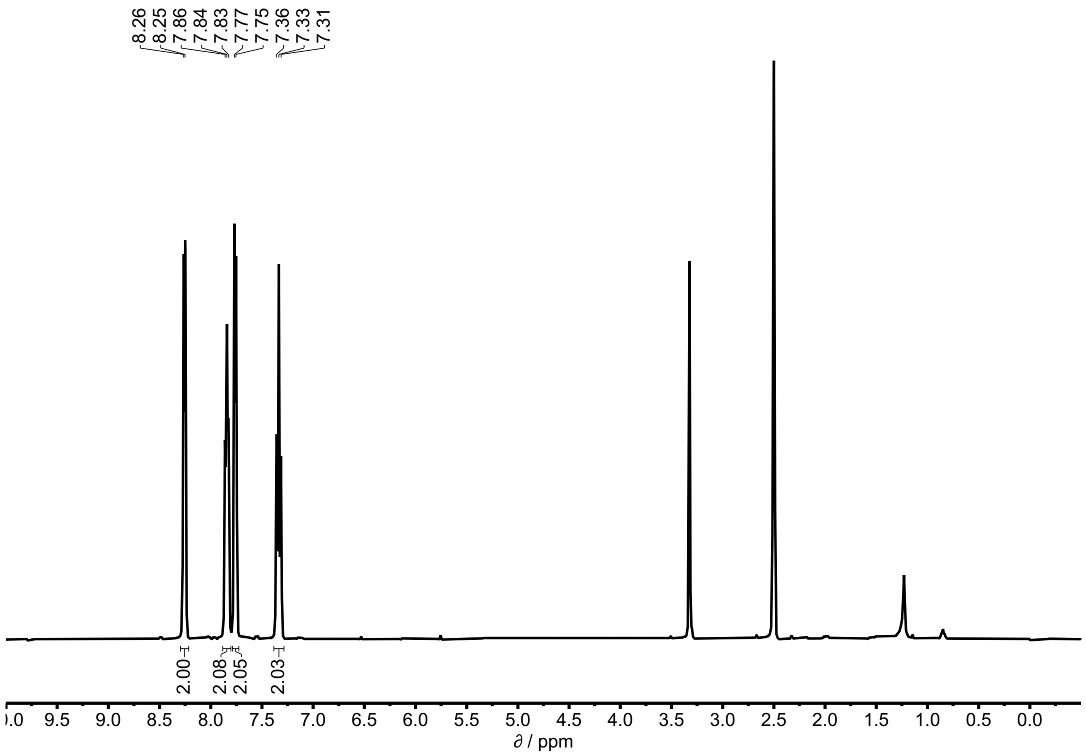

Figure S21. *^{1}H NMR spectrum (400 MHz, DMSO-d6) of compound **15**.*

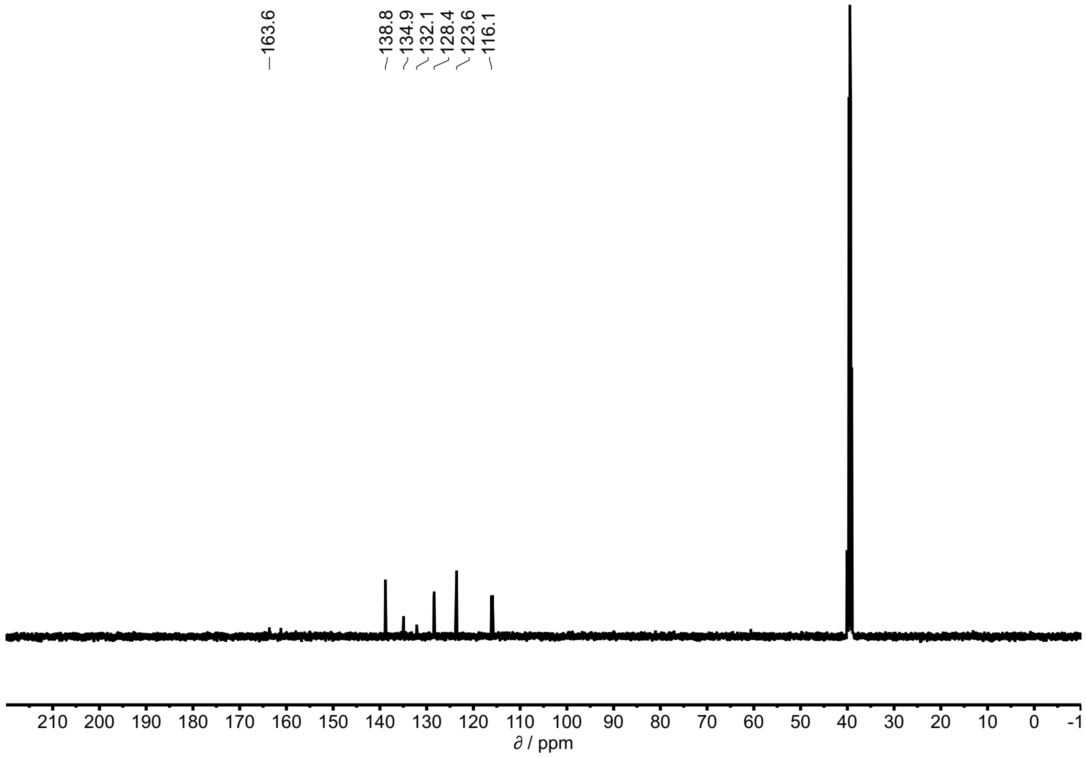

Figure S22. *^{13}C NMR spectrum (101 MHz, DMSO-d6) of compound **15**.*

4-(4-Chlorophenyl)pyridine 1-oxide (16)

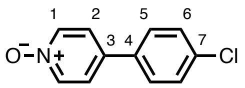

White solid (42 mg, 0.20 mmol, 41 % yield)

M.p.: 166 – 168 °C

^{1}H NMR (400 MHz, DMSO-*d*$_6$): δ_H = 8.27 (d, J = 7.3 Hz, 2H, H-1), 7.83 – 7.77 (m, 4H, H-2, H-5), 7.55 (d, J = 8.7 Hz, 2H, H-6).

^{13}C NMR (101 MHz, DMSO-*d*$_6$): δ_C = 139.0 (C-1), 134.6 (C-4), 134.5 (C-3), 133.6 (C-7), 129.2 (C-6), 128.0 (C-5), 123.7 (C-2).

HRMS (ES+): calculated for $C_{11}H_9ClNO$ 206.0373 [M+H$^+$], found 206.0382 [M+H$^+$].

FT-IR (ATR): ν_{max} 3387, 3110, 1472, 1397, 1244, 1180, 1101, 1024, 816, 753 cm^{-1}.

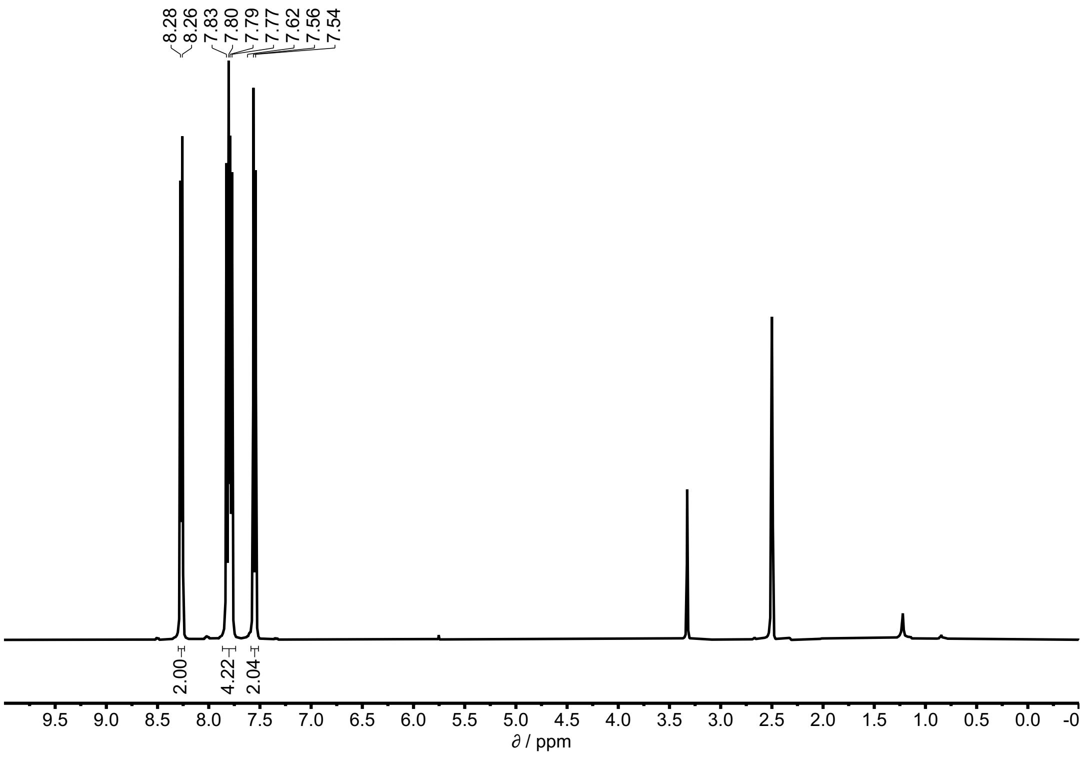

Figure S23. *¹H NMR spectrum (400 MHz, DMSO-d₆) of compound* ***16****.*

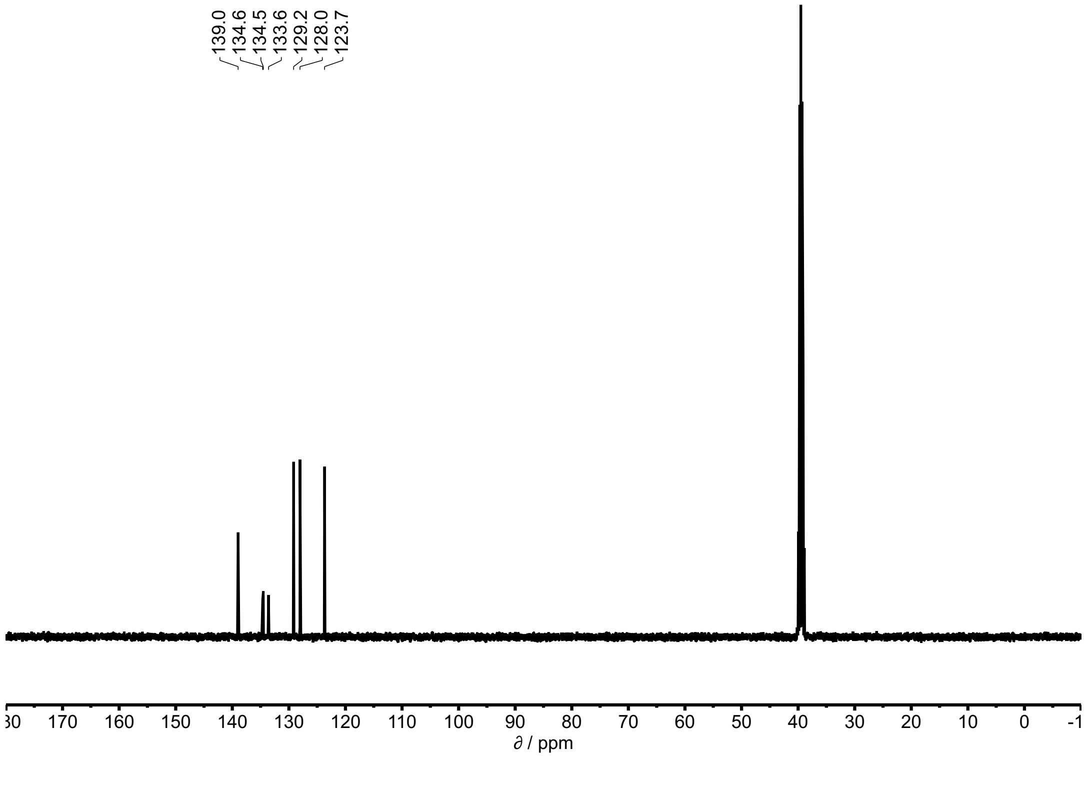

Figure S24. *¹³C NMR spectrum (101 MHz, DMSO-d₆) of compound* ***16****.*

4-(4-Vromophenyl)pyridine 1-oxide (17)

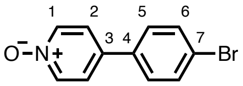

Off-white solid (35 mg, 0.14 mmol, 28 % yield)

M.p.: 162 – 164 °C.

^{1}H NMR (400 MHz, DMSO-*d*$_6$): δ_H = 8.27 (d, *J* = 7.3 Hz, 2H, H-1), 7.80 – 7.75 (m, 4H, H-2, H-5), 7.70 (d, *J* = 8.7 Hz, 2H, H-6).

^{13}C NMR (101 MHz, DMSO-*d*$_6$): δ_C = 139.0 (C-1), 134.9 (C-4), 134.7 (C-3), 132.1 (C-6), 128.3 (C-5), 123.7 (C-2), 122.3 (C-7).

HRMS (ES+): calculated for $C_{11}H_9BrNO$ 249.9868 [M+H$^+$], found 249.9871 [M+H$^+$].

FT-IR (ATR): ν_{max} 3387, 3111, 1472,1448, 1393, 1245, 1195, 1178, 1076, 1022, 1006, 849, 816, 741, 613 cm^{-1}.

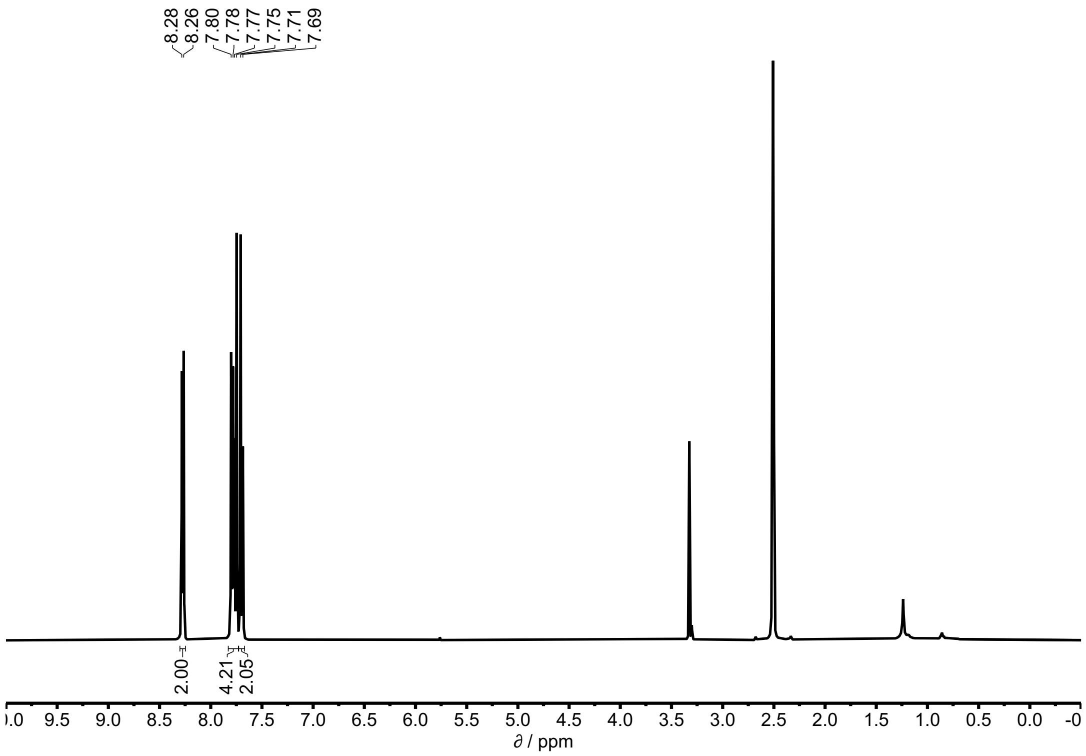

Figure S25. *^{1}H NMR spectrum (400 MHz, DMSO-d_6) of compound **17**.*

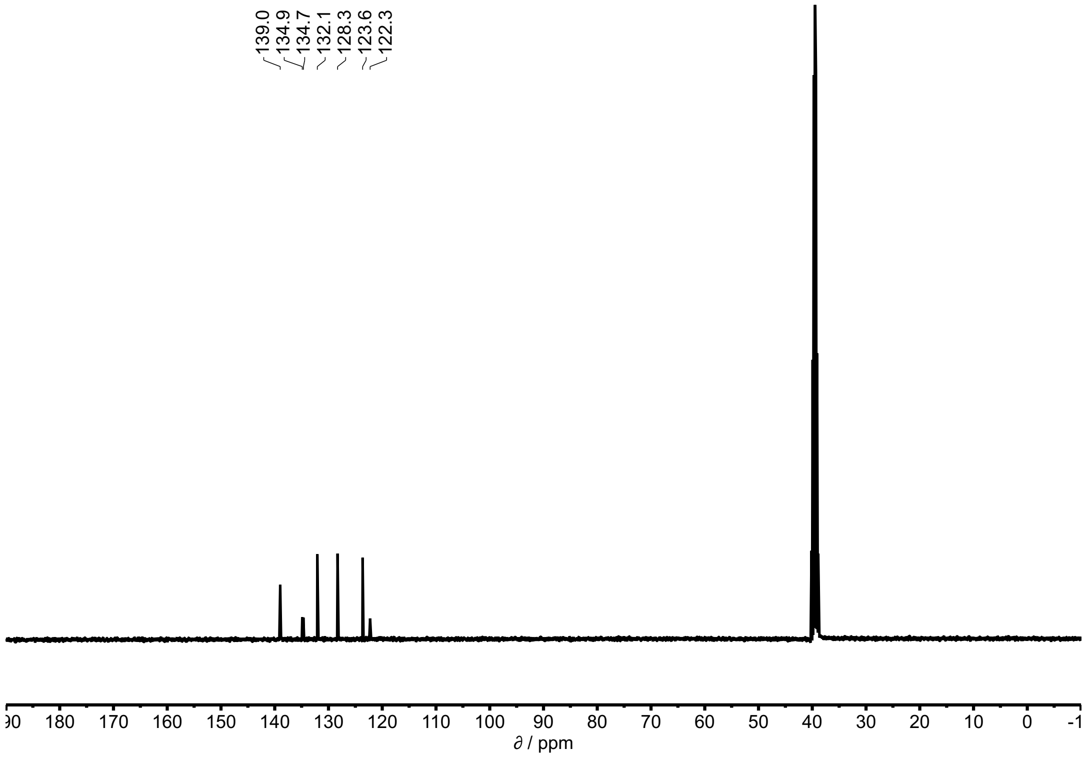

Figure S26. *^{13}C NMR spectrum (101 MHz, DMSO-d_6) of compound **17**.*

3. Isothermal titration calorimetry (ITC) experiments

In a typical ITC experiment, the host (**1-4**) was dissolved in HPLC grade water or chloroform with a concentration 30-40 times the expected dissociation constant, and the solution was loaded into the sample cell of the microcalorimeter. A 7-10 times more concentrated solution of guest (**5-17** and **PNO**) was loaded into the injection syringe. The number of injections was 35, and the volume of the injections was 8 µL. The thermogram peaks were integrated and thermodynamic parameters were calculated using the MicroCal PEAQ-ITC Analysis Software which uses the least-squares minimisation to obtain globally minimised parameters. In all cases the data fitted well to a simple 1:1 binding model.

3.1. Octapyridinium-super-aryl-extended calix[4]pyrrole 1

3.1.1. Complex C of the DMC

The C-value for this experiment is 141, so the data could be used to determine both K and ΔH°.

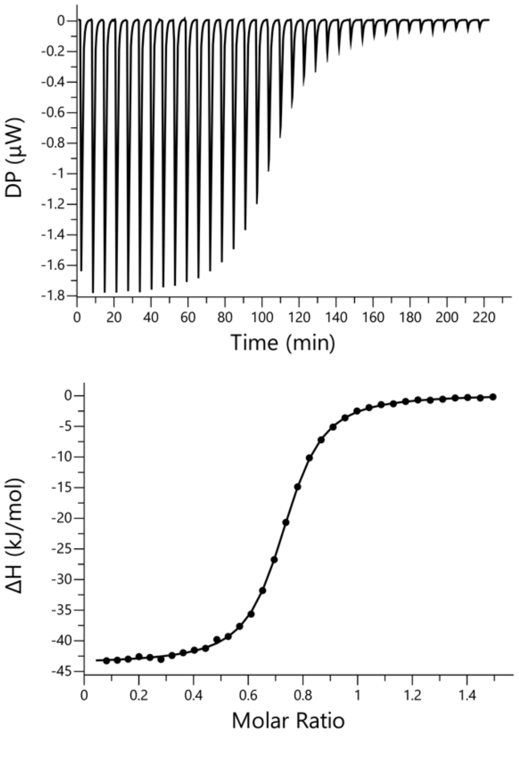

Figure S27. *ITC data for titration of* ***PNO*** *(0.28 mM) into* ***1*** *(0.04 mM) in water at 298 K. The raw data for each injection is shown (differential power, DP), along with the least-squares-fit of the enthalpy change per mole of guest (ΔH) to a 1:1 binding isotherm.*

3.1.2. Complex A of the DMC

The C-values for these experiments are in the range $10^5 – 10^8$, so the data could only be used to determine ΔH°. The values of K were determined separately by NMR competition experiments.

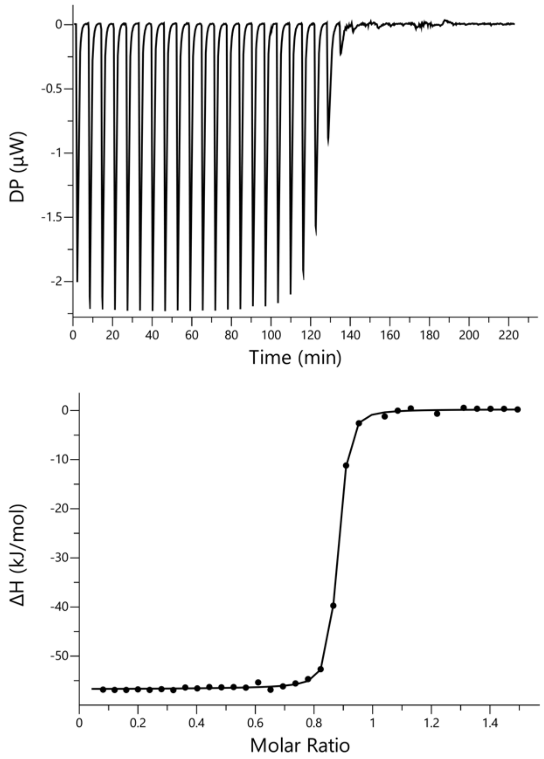

Figure S28. *ITC data for titration of **5** (0.28 mM) into **1** (0.04 mM) in water at 298 K. The raw data for each injection is shown (differential power, DP), along with the least-squares-fit of the enthalpy change per mole of guest (ΔH) to a 1:1 binding isotherm.*

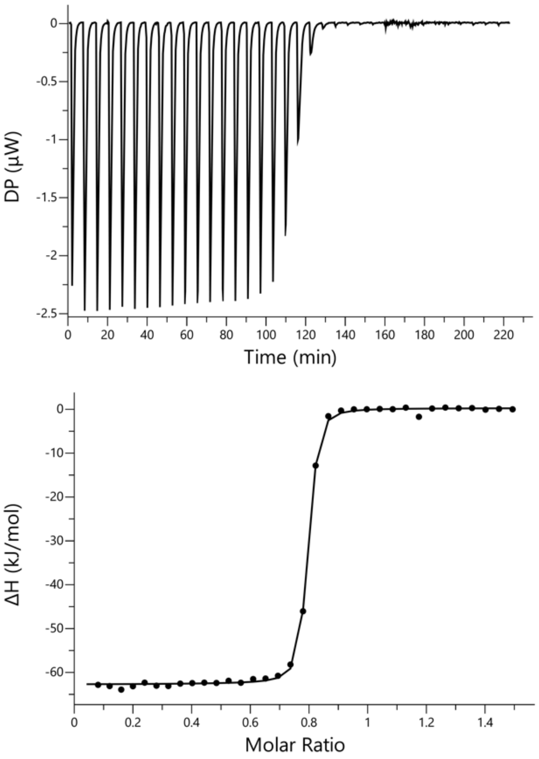

Figure S29. *ITC data for titration of* ***6*** *(0.28 mM) into* ***1*** *(0.04 mM) in water at 298 K. The raw data for each injection is shown (differential power, DP), along with the least-squares-fit of the enthalpy change per mole of guest (ΔH) to a 1:1 binding isotherm.*

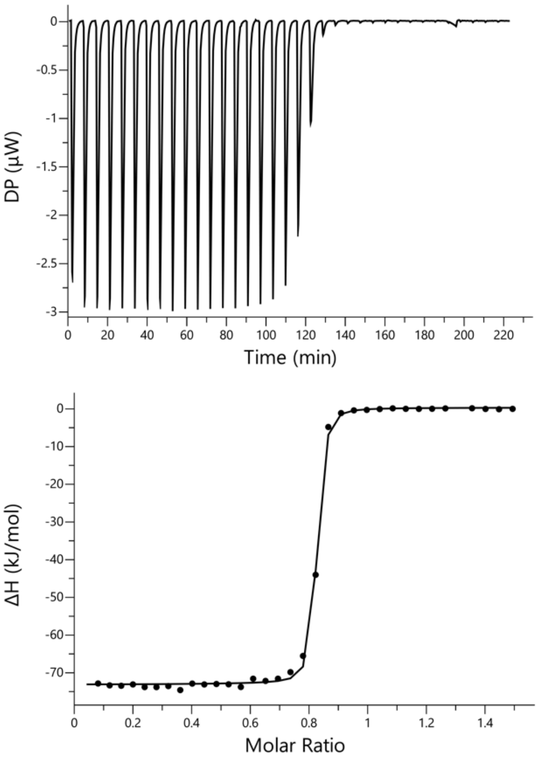

Figure S30. *ITC data for titration of* ***7*** *(0.28 mM) into* ***1*** *(0.04 mM) in water at 298 K. The raw data for each injection is shown (differential power, DP), along with the least-squares-fit of the enthalpy change per mole of guest (ΔH) to a 1:1 binding isotherm.*

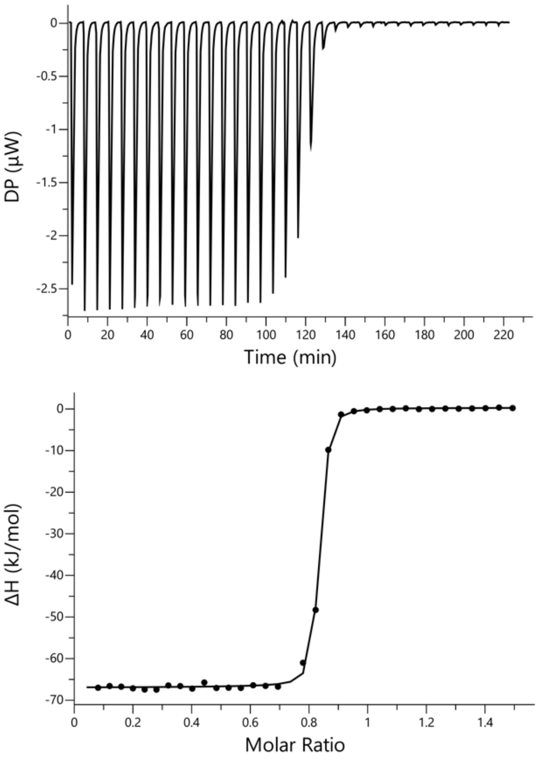

Figure S31. *ITC data for titration of **8** (0.28 mM) into **1** (0.04 mM) in water at 298 K. The raw data for each injection is shown (differential power, DP), along with the least-squares-fit of the enthalpy change per mole of guest (ΔH) to a 1:1 binding isotherm.*

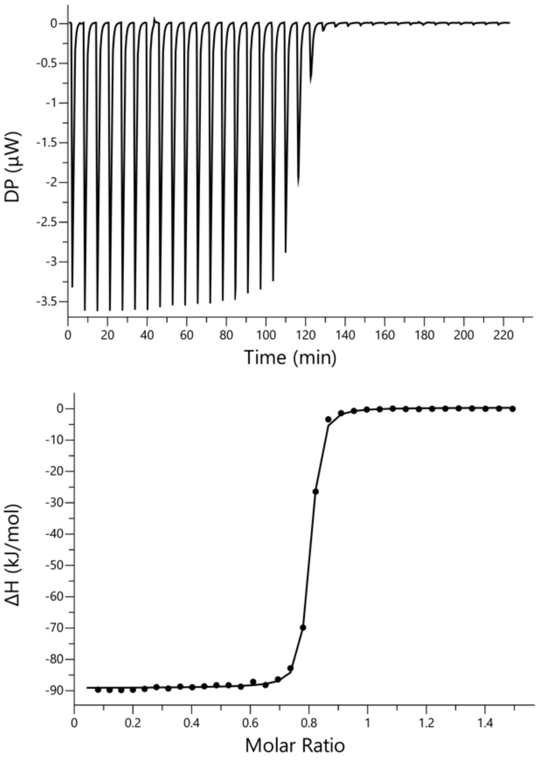

Figure S32. *ITC data for titration of **9** (0.28 mM) into **1** (0.04 mM) in water at 298 K. The raw data for each injection is shown (differential power, DP), along with the least-squares-fit of the enthalpy change per mole of guest (ΔH) to a 1:1 binding isotherm.*

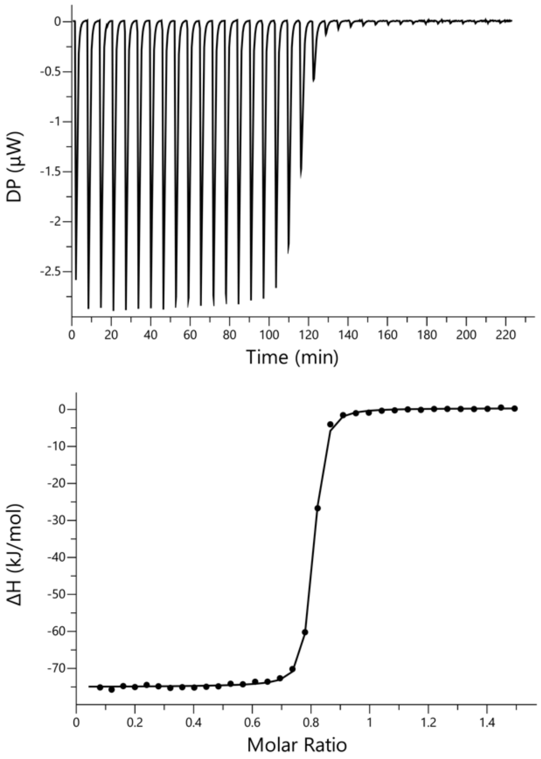

Figure S33. *ITC data for titration of* ***10*** *(0.28 mM) into* ***1*** *(0.04 mM) in water at 298 K. The raw data for each injection is shown (differential power, DP), along with the least-squares-fit of the enthalpy change per mole of guest (ΔH) to a 1:1 binding isotherm.*

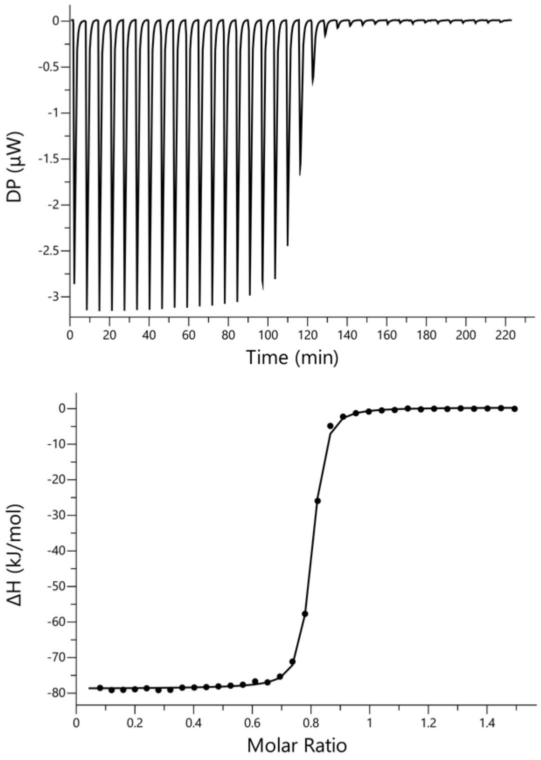

Figure S34. *ITC data for titration of* ***11*** *(0.28 mM) into* ***1*** *(0.04 mM) in water at 298 K. The raw data for each injection is shown (differential power, DP), along with the least-squares-fit of the enthalpy change per mole of guest (ΔH) to a 1:1 binding isotherm.*

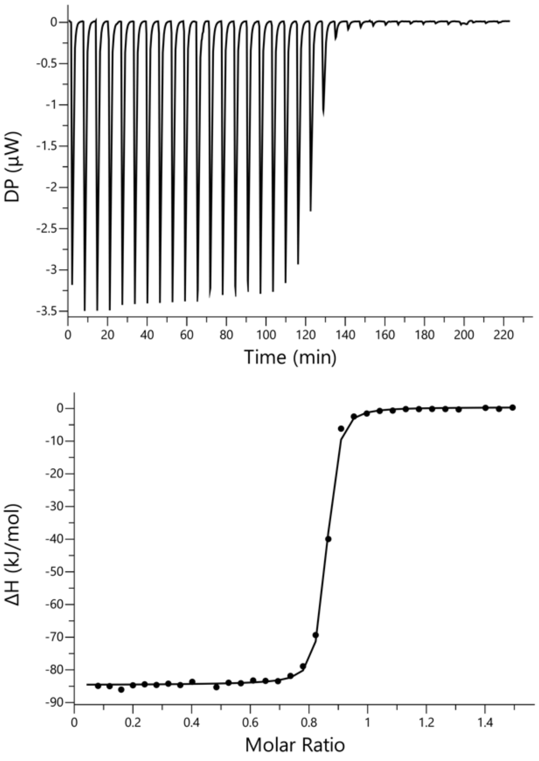

Figure S35. *ITC data for titration of **12** (0.28 mM) into **1** (0.04 mM) in water at 298 K. The raw data for each injection is shown (differential power, DP), along with the least-squares-fit of the enthalpy change per mole of guest (ΔH) to a 1:1 binding isotherm.*

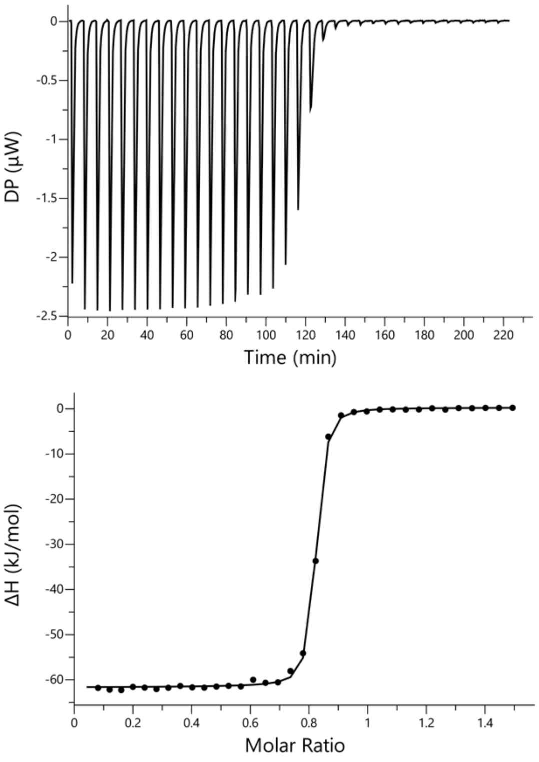

Figure S36. *ITC data for titration of **13** (0.28 mM) into **1** (0.04 mM) in water at 298 K. The raw data for each injection is shown (differential power, DP), along with the least-squares-fit of the enthalpy change per mole of guest (ΔH) to a 1:1 binding isotherm.*

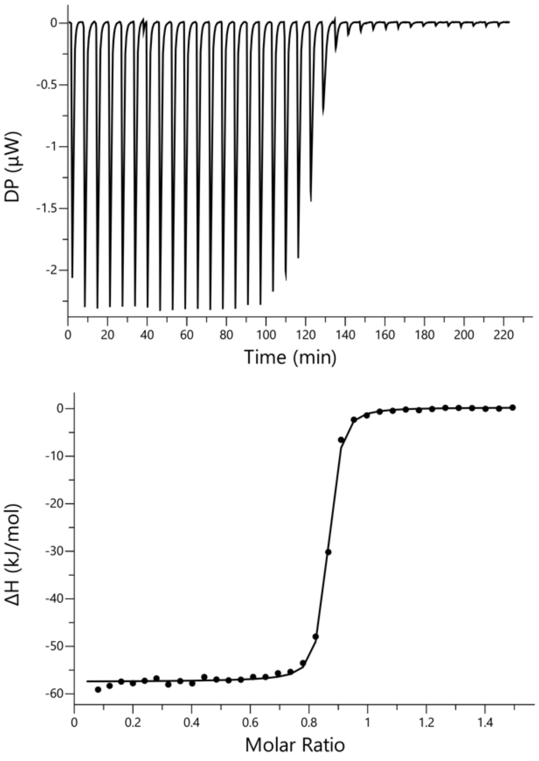

Figure S37. *ITC data for titration of **14** (0.28 mM) into **1** (0.04 mM) in water at 298 K. The raw data for each injection is shown (differential power, DP), along with the least-squares-fit of the enthalpy change per mole of guest (ΔH) to a 1:1 binding isotherm.*

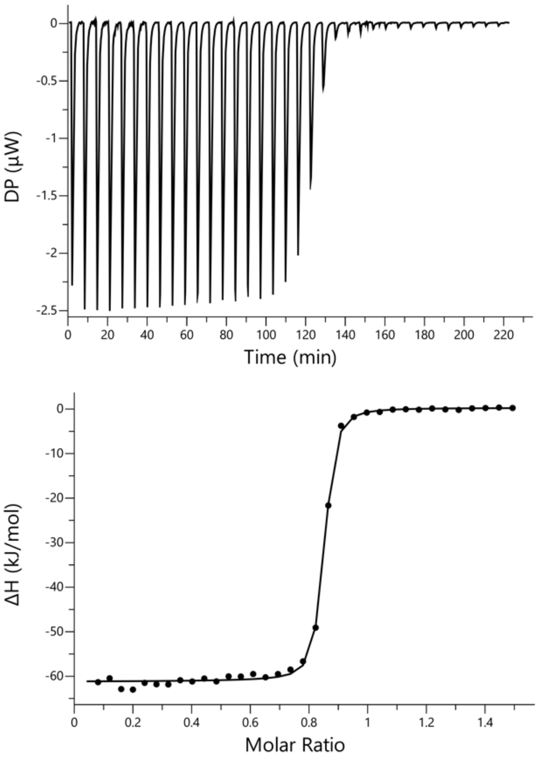

Figure S38. *ITC data for titration of **15** (0.28 mM) into **1** (0.04 mM) in water at 298 K. The raw data for each injection is shown (differential power, DP), along with the least-squares-fit of the enthalpy change per mole of guest (ΔH) to a 1:1 binding isotherm.*

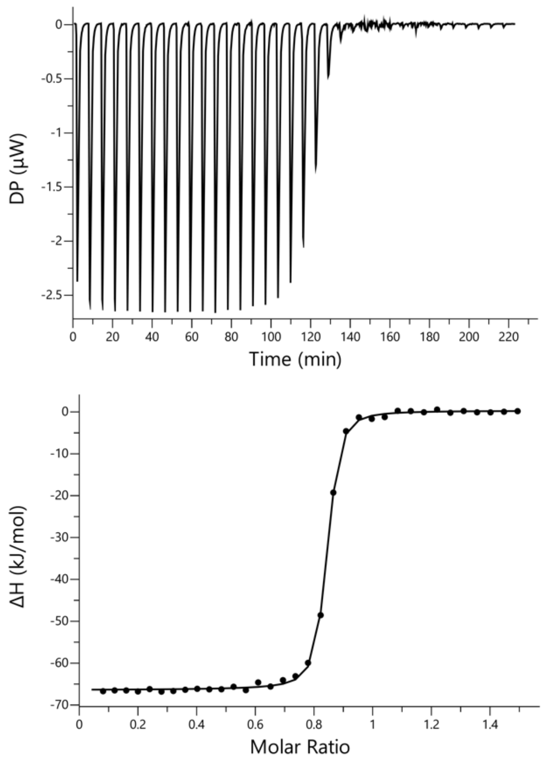

Figure S39. *ITC data for titration of **16** (0.28 mM) into **1** (0.04 mM) in water at 298 K. The raw data for each injection is shown (differential power, DP), along with the least-squares-fit of the enthalpy change per mole of guest (ΔH) to a 1:1 binding isotherm.*

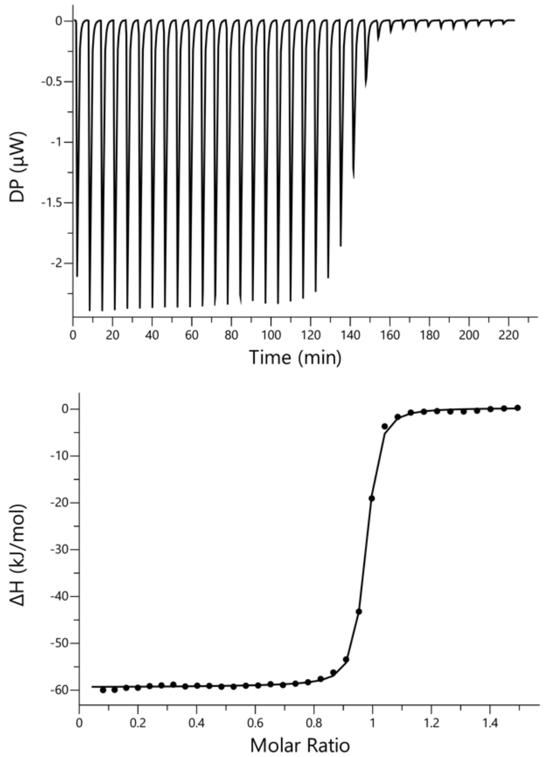

Figure S40. *ITC data for titration of **17** (0.28 mM) into **1** (0.04 mM) in water at 298 K. The raw data for each injection is shown (differential power, DP), along with the least-squares-fit of the enthalpy change per mole of guest (ΔH) to a 1:1 binding isotherm.*

3.2. Tetrapyridinium-aryl-extended calix[4]pyrrole 3

3.2.1. Complex D of the DMC

The C-value for this experiment is 13, so the data could be used to determine both K and ΔH°.

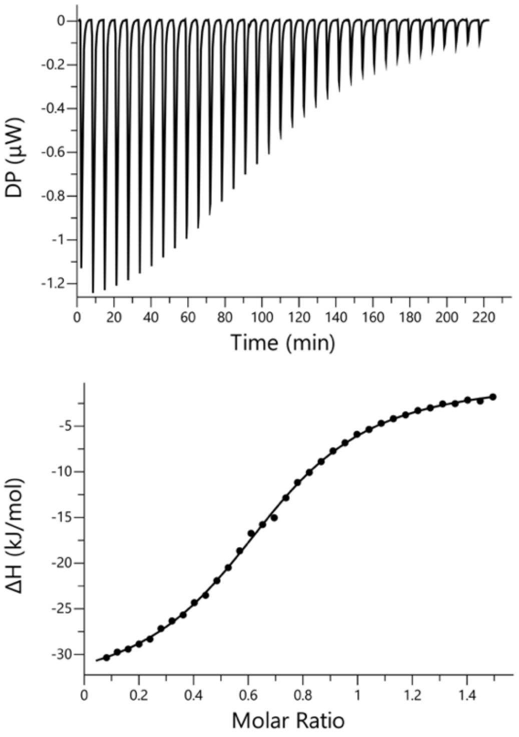

Figure S41. *ITC data for titration of* ***PNO*** *(0.28 mM) into* ***3*** *(0.04 mM) in water at 298 K. The raw data for each injection is shown (differential power, DP), along with the least-squares-fit of the enthalpy change per mole of guest (ΔH) to a 1:1 binding isotherm.*

3.2.2. Complex B of the DMC

The C-values for these experiments are in the range 37 – 54, so the data could be used to determine both K and ΔH°.

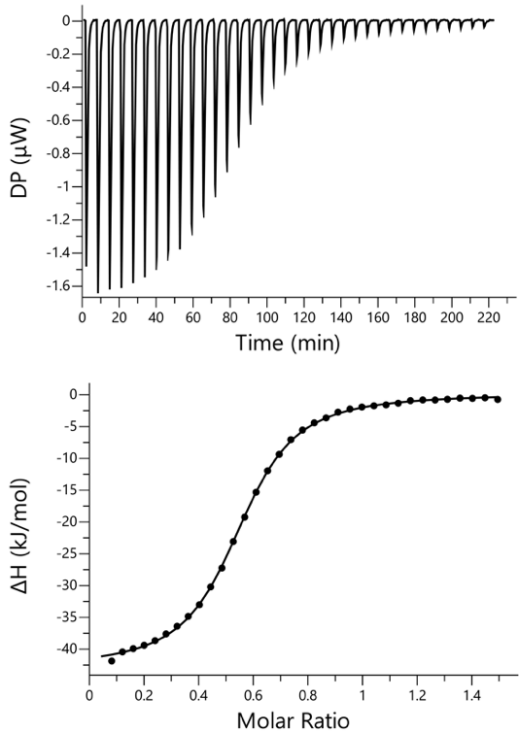

Figure S42. *ITC data for titration of **5** (0.28 mM) into **3** (0.04 mM) in water at 298 K. The raw data for each injection is shown (differential power, DP), along with the least-squares-fit of the enthalpy change per mole of guest (ΔH) to a 1:1 binding isotherm.*

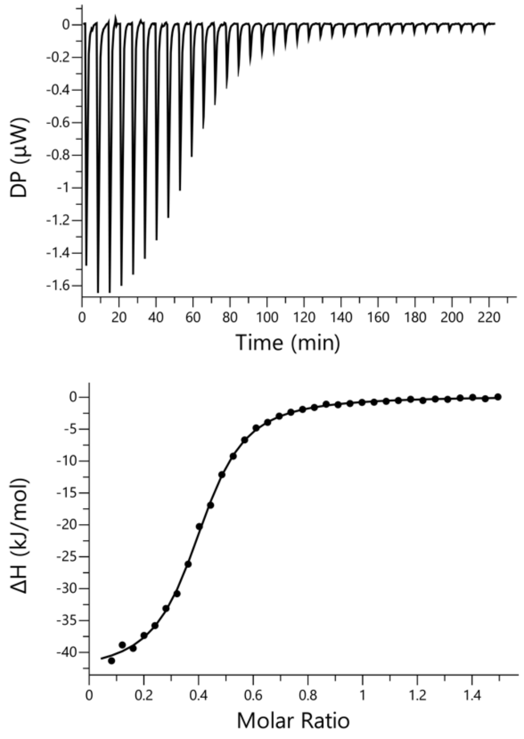

Figure S43. *ITC data for titration of* ***6*** *(0.28 mM) into* ***3*** *(0.04 mM) in water at 298 K. The raw data for each injection is shown (differential power, DP), along with the least-squares-fit of the enthalpy change per mole of guest (ΔH) to a 1:1 binding isotherm.*

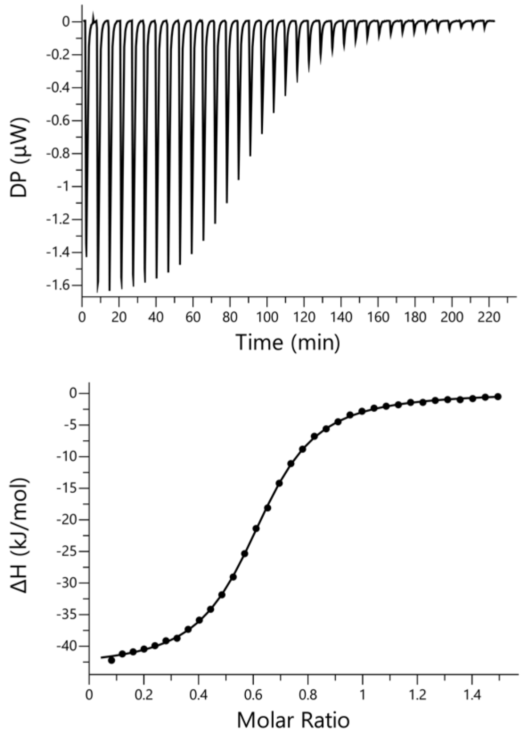

Figure S44. *ITC data for titration of* ***7*** *(0.28 mM) into* ***3*** *(0.04 mM) in water at 298 K. The raw data for each injection is shown (differential power, DP), along with the least-squares-fit of the enthalpy change per mole of guest (ΔH) to a 1:1 binding isotherm.*

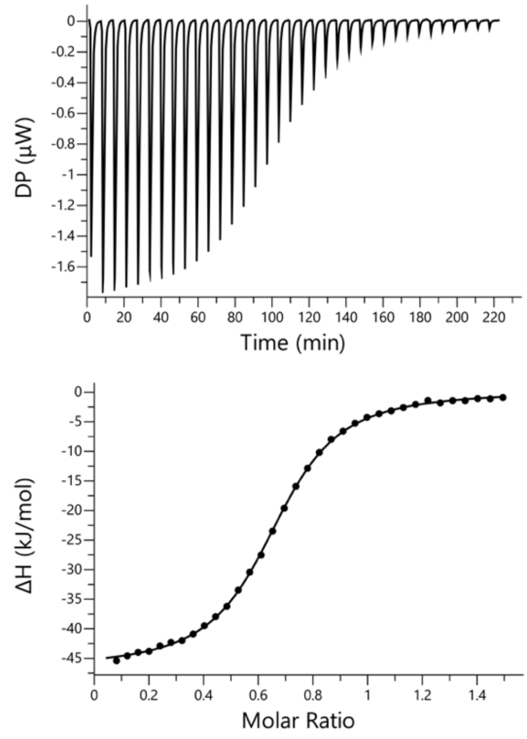

Figure S45. *ITC data for titration of **8** (0.28 mM) into **3** (0.04 mM) in water at 298 K. The raw data for each injection is shown (differential power, DP), along with the least-squares-fit of the enthalpy change per mole of guest (ΔH) to a 1:1 binding isotherm.*

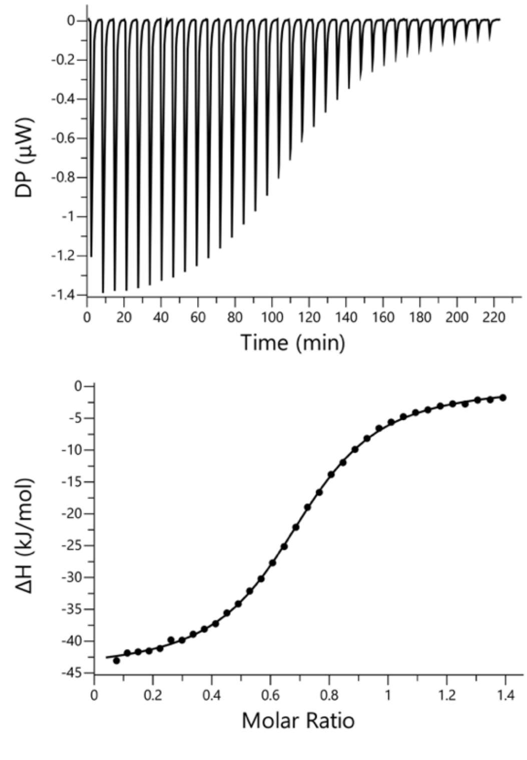

Figure S46. *ITC data for titration of **9** (0.28 mM) into **3** (0.04 mM) in water at 298 K. The raw data for each injection is shown (differential power, DP), along with the least-squares-fit of the enthalpy change per mole of guest (ΔH) to a 1:1 binding isotherm.*

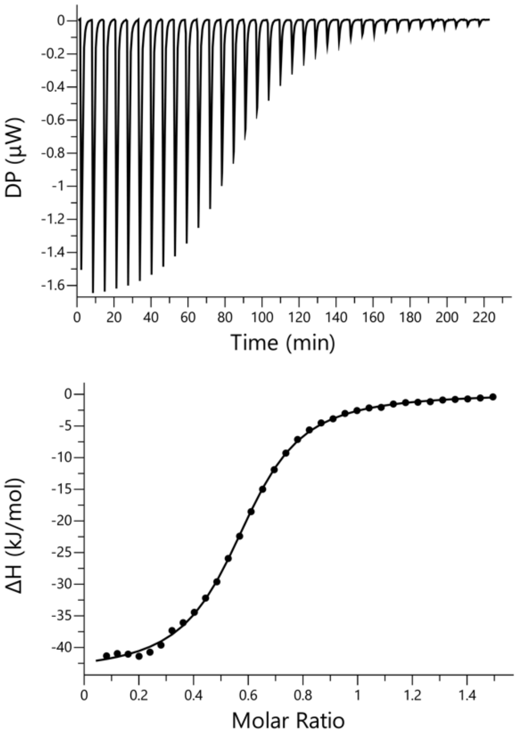

Figure S47. *ITC data for titration of **10** (0.28 mM) into **3** (0.04 mM) in water at 298 K. The raw data for each injection is shown (differential power, DP), along with the least-squares-fit of the enthalpy change per mole of guest (ΔH) to a 1:1 binding isotherm.*

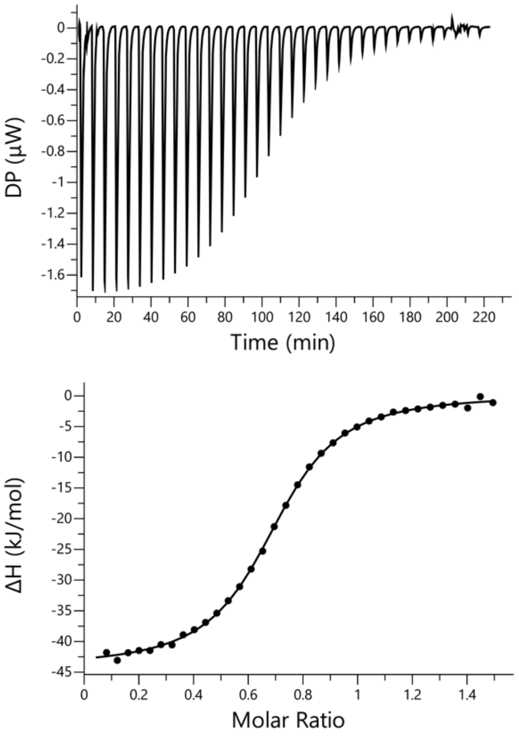

Figure S48. *ITC data for titration of **11** (0.28 mM) into **3** (0.04 mM) in water at 298 K. The raw data for each injection is shown (differential power, DP), along with the least-squares-fit of the enthalpy change per mole of guest (ΔH) to a 1:1 binding isotherm.*

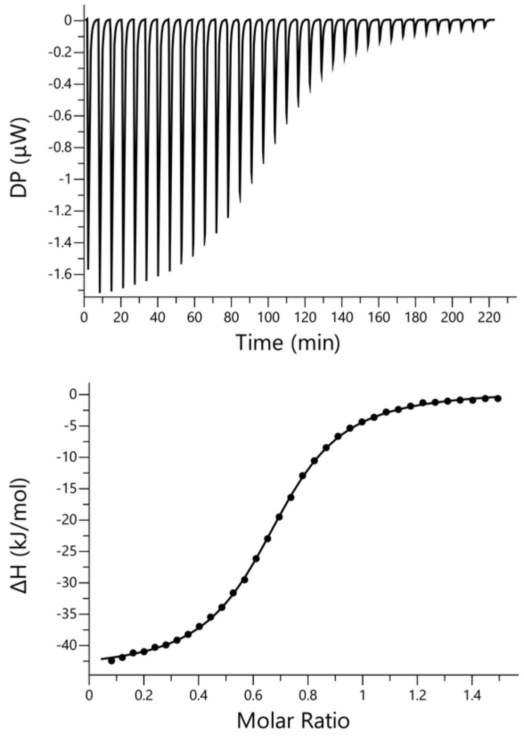

Figure S49. *ITC data for titration of* ***12*** *(0.28 mM) into* ***3*** *(0.04 mM) in water at 298 K. The raw data for each injection is shown (differential power, DP), along with the least-squares-fit of the enthalpy change per mole of guest (ΔH) to a 1:1 binding isotherm.*

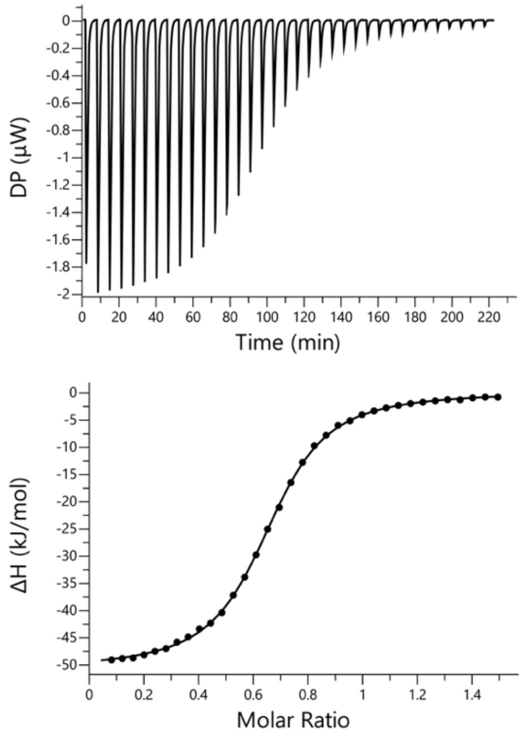

Figure S50. *ITC data for titration of* ***13*** *(0.28 mM) into* ***3*** *(0.04 mM) in water at 298 K. The raw data for each injection is shown (differential power, DP), along with the least-squares-fit of the enthalpy change per mole of guest (ΔH) to a 1:1 binding isotherm.*

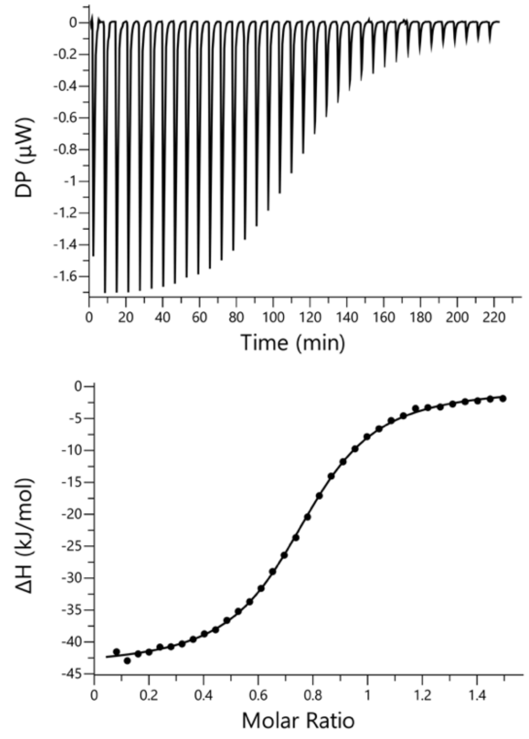

Figure S51. *ITC data for titration of* ***14*** *(0.28 mM) into* ***3*** *(0.04 mM) in water at 298 K. The raw data for each injection is shown (differential power, DP), along with the least-squares-fit of the enthalpy change per mole of guest (ΔH) to a 1:1 binding isotherm.*

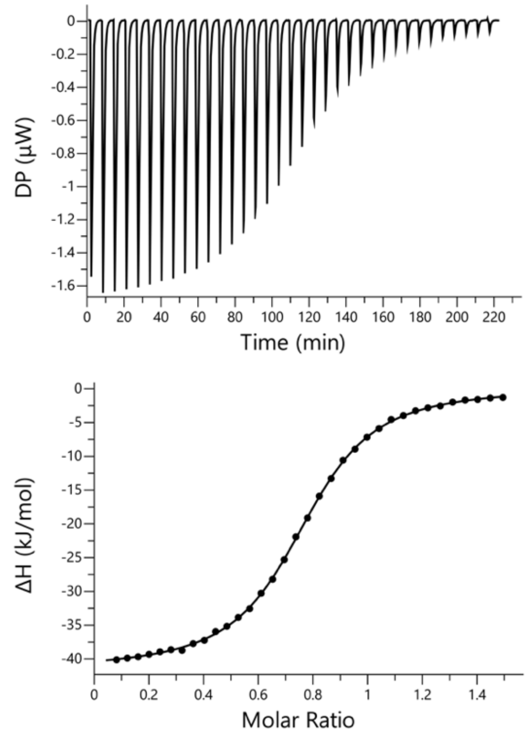

Figure S52. *ITC data for titration of* ***15*** *(0.28 mM) into* ***3*** *(0.04 mM) in water at 298 K. The raw data for each injection is shown (differential power, DP), along with the least-squares-fit of the enthalpy change per mole of guest (ΔH) to a 1:1 binding isotherm.*

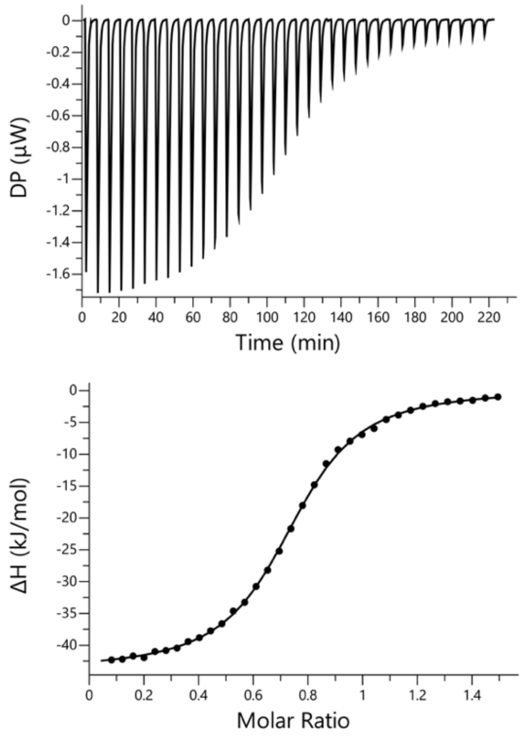

Figure S53. *ITC data for titration of* ***16*** *(0.28 mM) into* ***3*** *(0.04 mM) in water at 298 K. The raw data for each injection is shown (differential power, DP), along with the least-squares-fit of the enthalpy change per mole of guest (ΔH) to a 1:1 binding isotherm.*

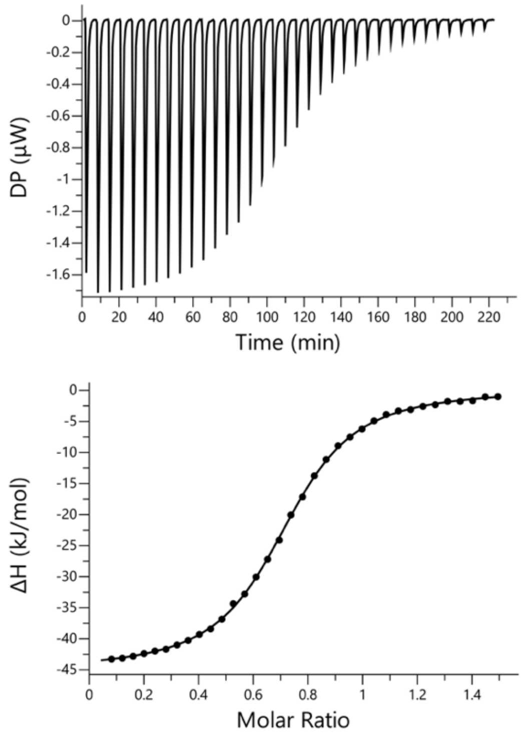

Figure S54. *ITC data for titration of* ***17*** *(0.28 mM) into* ***3*** *(0.04 mM) in water at 298 K. The raw data for each injection is shown (differential power, DP), along with the least-squares-fit of the enthalpy change per mole of guest (ΔH) to a 1:1 binding isotherm.*

3.3. Octachloro-super-aryl-extended calix[4]pyrrole 2

3.3.1. Complex C of the DMC

The C-value for this experiment is 223, so the data could be used to determine both K and ΔH°.

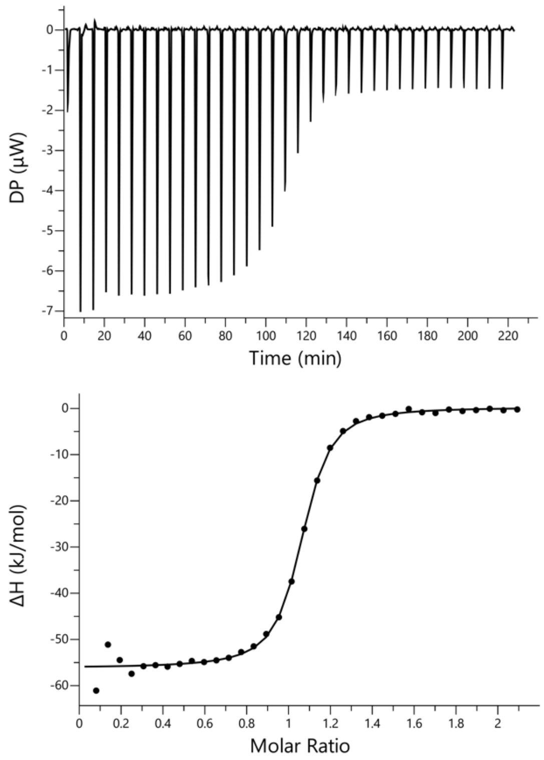

Figure S55. *ITC data for titration of **PNO** (0.30 mM) into **2** (0.03 mM) in chloroform at 298 K. The raw data for each injection is shown (differential power, DP), along with the least-squares-fit of the enthalpy change per mole of guest (ΔH) to a 1:1 binding isotherm.*

3.3.2. Complex A of the DMC

The C-values for these experiments are in the range 10^3 – 10^5 so the data could only be used to determine ΔH°. The values of K were determined separately by NMR competition experiments

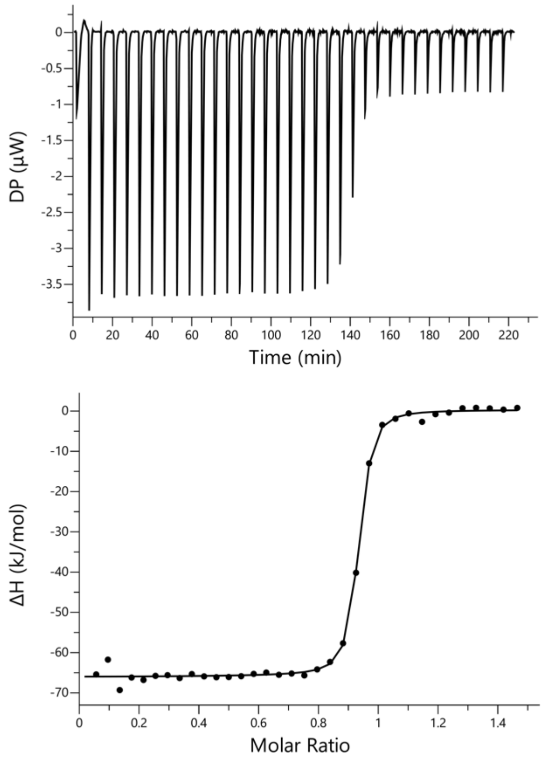

Figure S56. *ITC data for titration of* ***5*** *(0.30 mM) into* ***2*** *(0.03 mM) in chloroform at 298 K. The raw data for each injection is shown (differential power, DP), along with the least-squares-fit of the enthalpy change per mole of guest (ΔH) to a 1:1 binding isotherm.*

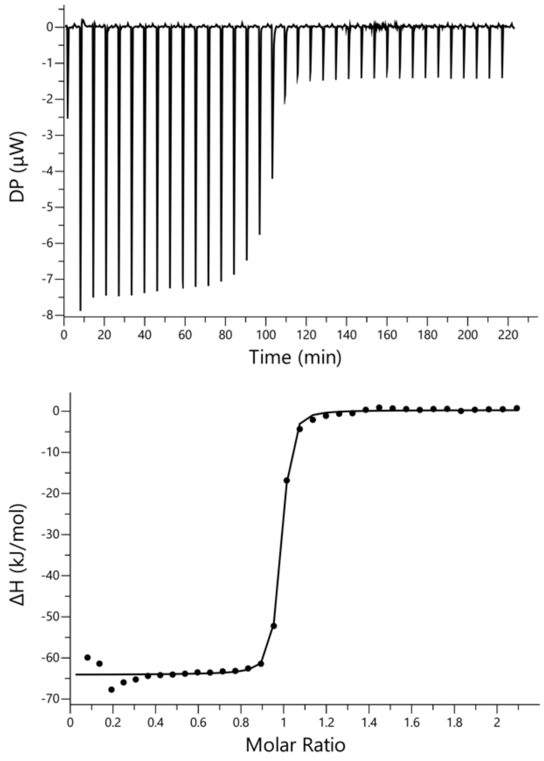

Figure S57. *ITC data for titration of* ***6*** *(0.30 mM) into* ***2*** *(0.03 mM) in chloroform at 298 K. The raw data for each injection is shown (differential power, DP), along with the least-squares-fit of the enthalpy change per mole of guest (ΔH) to a 1:1 binding isotherm.*

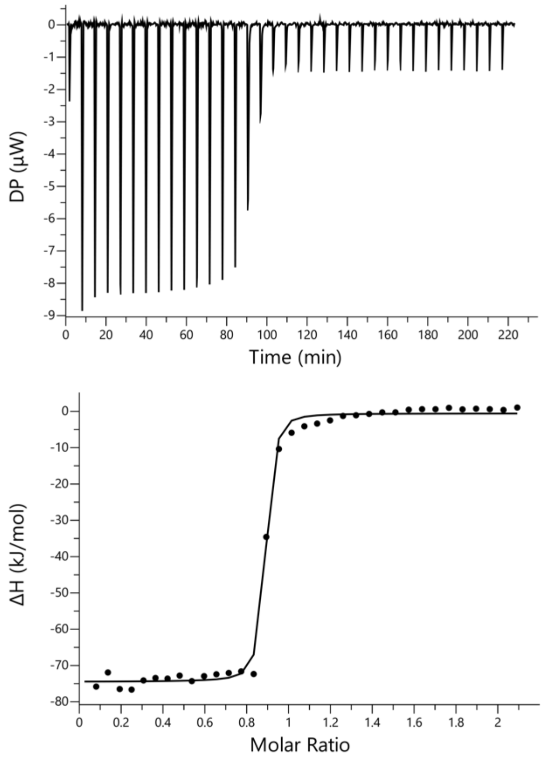

Figure S58. *ITC data for titration of* ***7*** *(0.30 mM) into* ***2*** *(0.03 mM) in chloroform at 298 K. The raw data for each injection is shown (differential power, DP), along with the least-squares-fit of the enthalpy change per mole of guest (ΔH) to a 1:1 binding isotherm.*

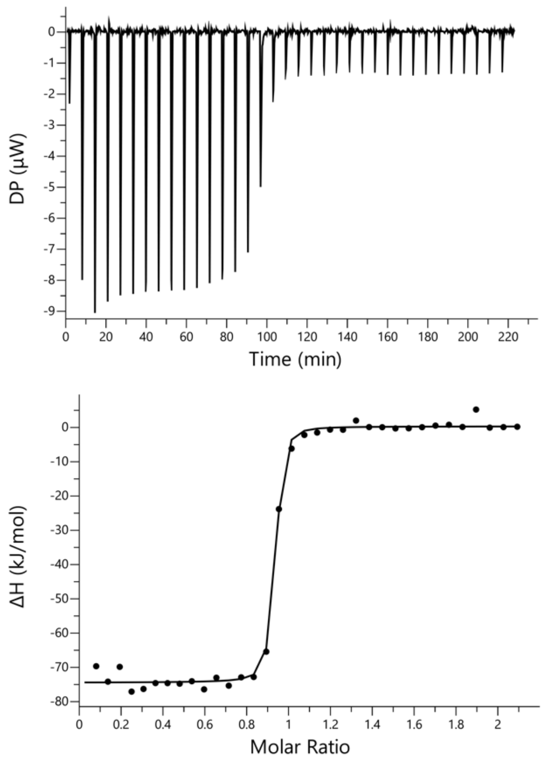

Figure S59. *ITC data for titration of **8** (0.30 mM) into **2** (0.03 mM) in chloroform at 298 K. The raw data for each injection is shown (differential power, DP), along with the least-squares-fit of the enthalpy change per mole of guest (ΔH) to a 1:1 binding isotherm.*

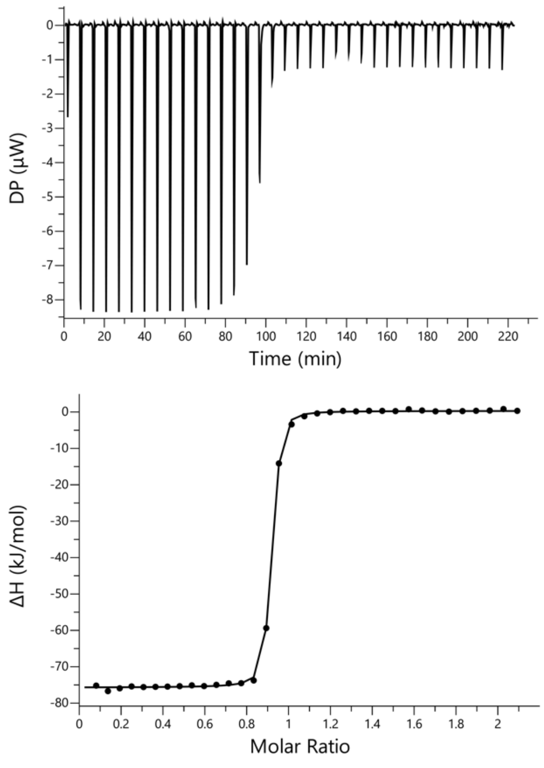

Figure S60. *ITC data for titration of **9** (0.30 mM) into **2** (0.03 mM) in chloroform at 298 K. The raw data for each injection is shown (differential power, DP), along with the least-squares-fit of the enthalpy change per mole of guest (ΔH) to a 1:1 binding isotherm.*

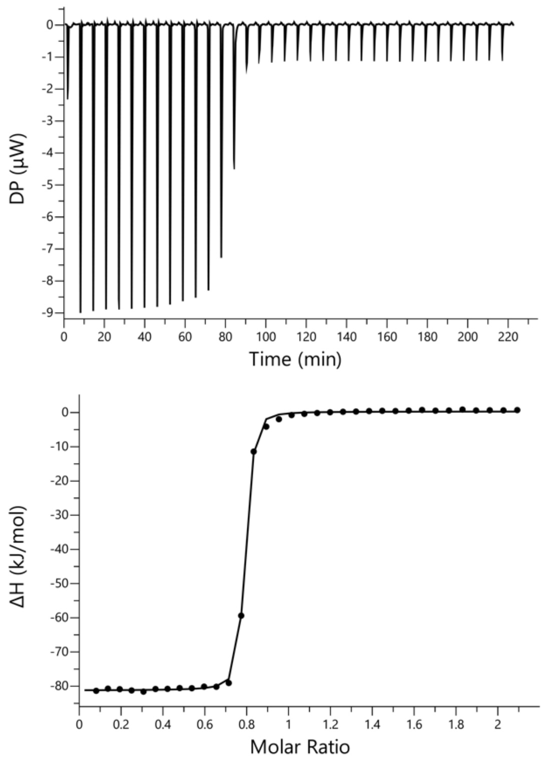

Figure S61. *ITC data for titration of **10** (0.30 mM) into **2** (0.03 mM) in chloroform at 298 K. The raw data for each injection is shown (differential power, DP), along with the least-squares-fit of the enthalpy change per mole of guest (ΔH) to a 1:1 binding isotherm.*

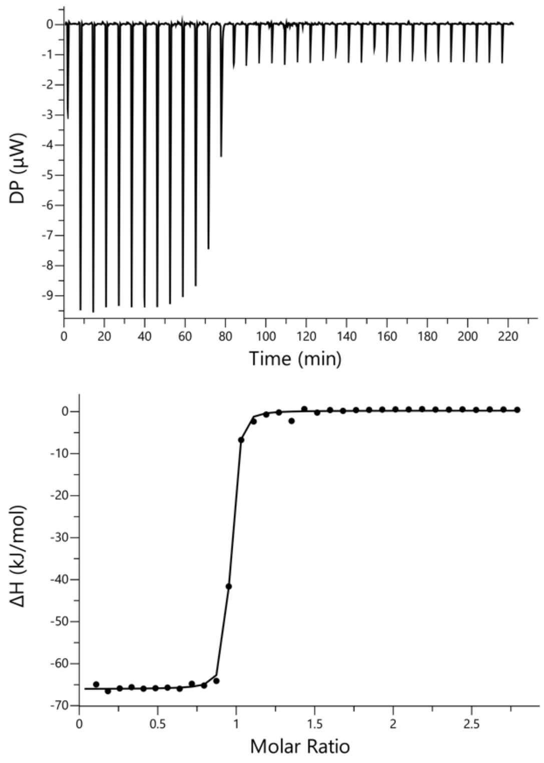

Figure S62. *ITC data for titration of **11** (0.30 mM) into **2** (0.03 mM) in chloroform at 298 K. The raw data for each injection is shown (differential power, DP), along with the least-squares-fit of the enthalpy change per mole of guest (ΔH) to a 1:1 binding isotherm.*

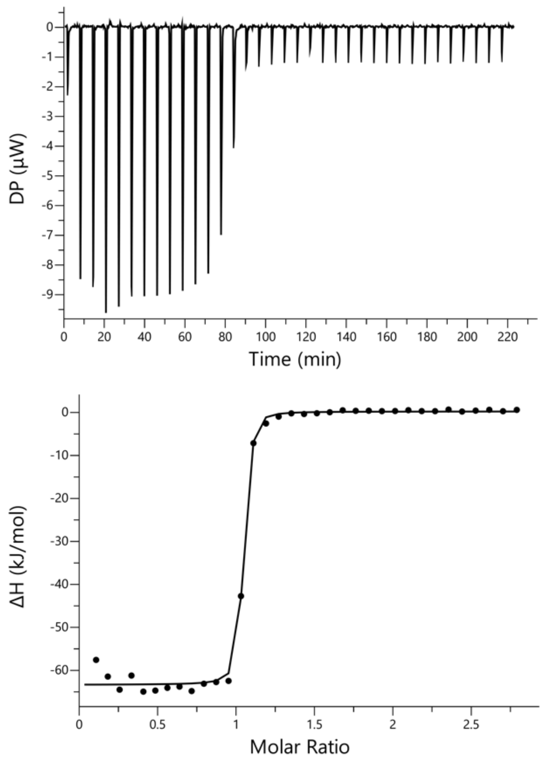

Figure S63. *ITC data for titration of **12** (0.30 mM) into **2** (0.03 mM) in chloroform at 298 K. The raw data for each injection is shown (differential power, DP), along with the least-squares-fit of the enthalpy change per mole of guest (ΔH) to a 1:1 binding isotherm.*

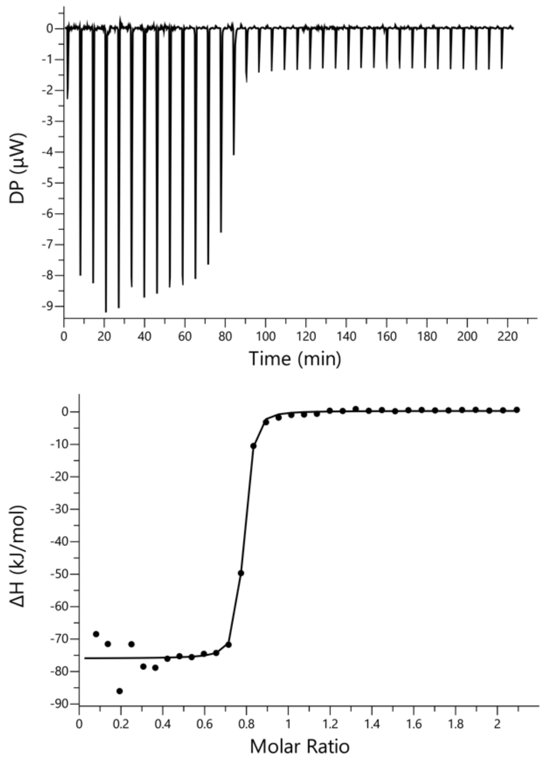

Figure S64. *ITC data for titration of **13** (0.30 mM) into **2** (0.03 mM) in chloroform at 298 K. The raw data for each injection is shown (differential power, DP), along with the least-squares-fit of the enthalpy change per mole of guest (ΔH) to a 1:1 binding isotherm.*

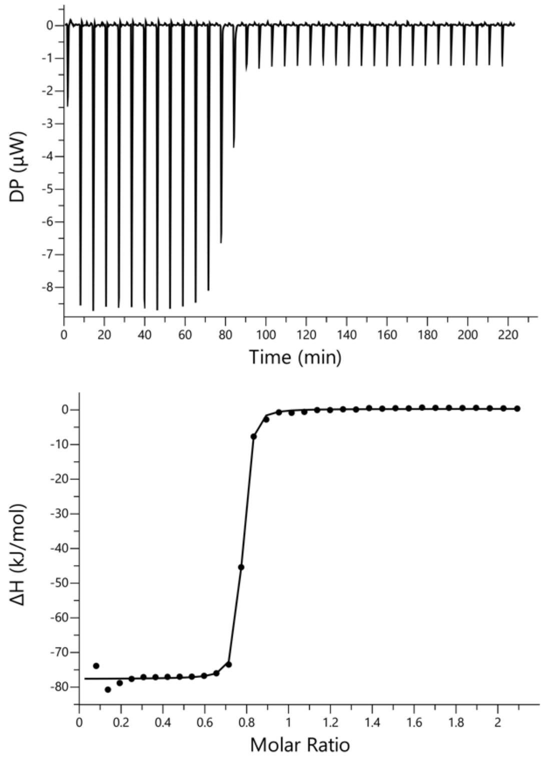

Figure S65. *ITC data for titration of **14** (0.30 mM) into **2** (0.03 mM) in chloroform at 298 K. The raw data for each injection is shown (differential power, DP), along with the least-squares-fit of the enthalpy change per mole of guest (ΔH) to a 1:1 binding isotherm.*

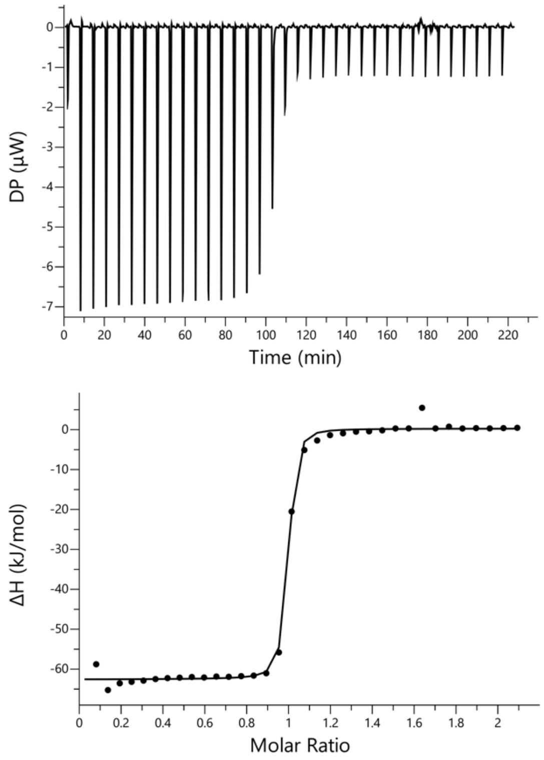

Figure S66. *ITC data for titration of **15** (0.30 mM) into **2** (0.03 mM) in chloroform at 298 K. The raw data for each injection is shown (differential power, DP), along with the least-squares-fit of the enthalpy change per mole of guest (ΔH) to a 1:1 binding isotherm.*

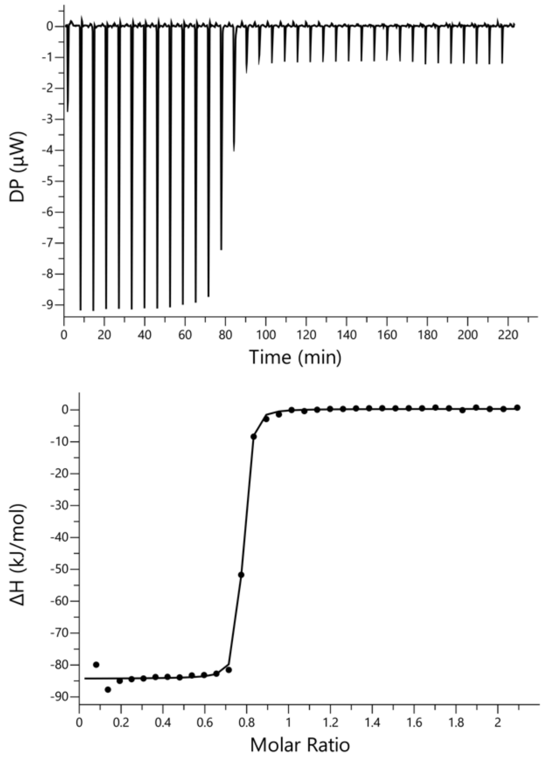

Figure S67. *ITC data for titration of* ***16*** *(0.30 mM) into* ***2*** *(0.03 mM) in chloroform at 298 K. The raw data for each injection is shown (differential power, DP), along with the least-squares-fit of the enthalpy change per mole of guest (ΔH) to a 1:1 binding isotherm.*

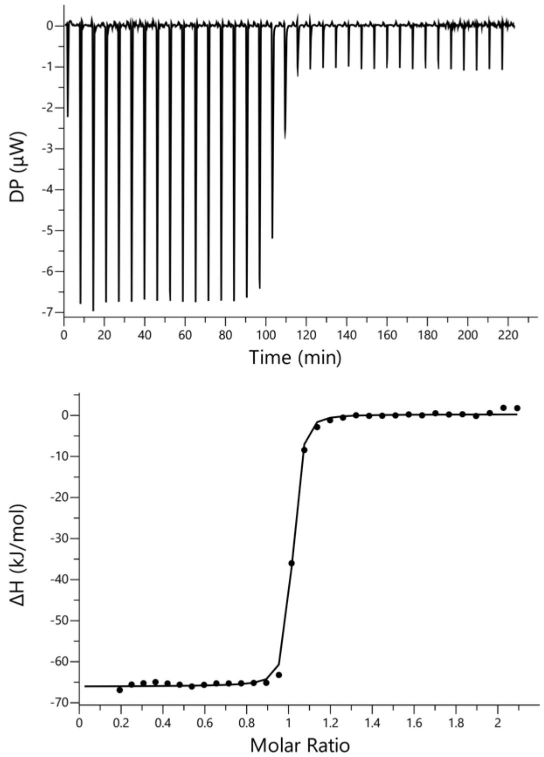

Figure S68. *ITC data for titration of* ***17*** *(0.30 mM) into* ***2*** *(0.03 mM) in chloroform at 298 K. The raw data for each injection is shown (differential power, DP), along with the least-squares-fit of the enthalpy change per mole of guest (ΔH) to a 1:1 binding isotherm.*

3.4. Tetrachloro-aryl-extened calix[4]pyrrole 4

3.4.1. Complex D of the DMC

The C-value for this experiment is 39, so the data could be used to determine both K and ΔH°

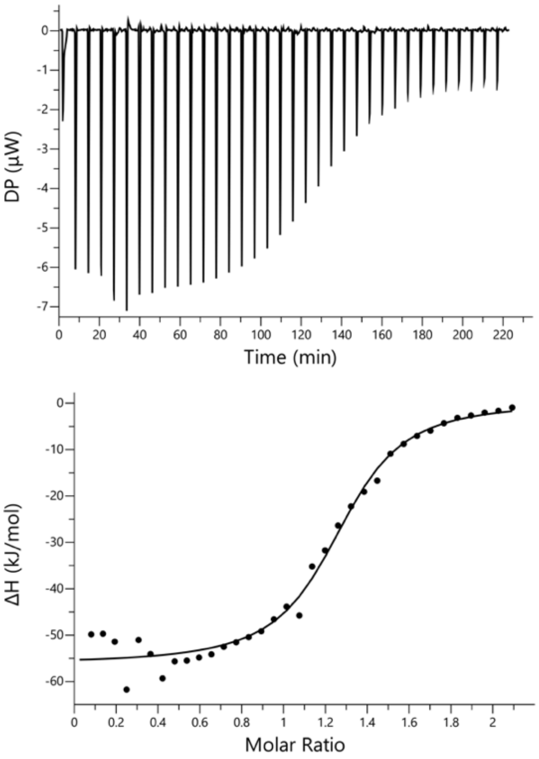

Figure S69. *ITC data for titration of* ***PNO*** *(0.30 mM) into* ***4*** *(0.03 mM) in chloroform at 298 K. The raw data for each injection is shown (differential power, DP), along with the least-squares-fit of the enthalpy change per mole of guest (ΔH) to a 1:1 binding isotherm.*

3.4.2. Complex B of the DMC

The C-values for these experiments are in the range 16 – 97, so the data could be used to determine both K and ΔH°.

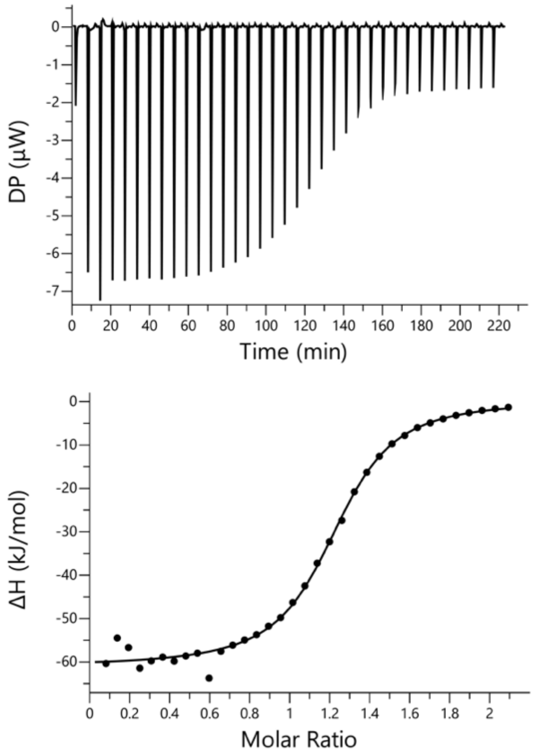

Figure S70. *ITC data for titration of **5** (0.30 mM) into **4** (0.03 mM) in chloroform at 298 K. The raw data for each injection is shown (differential power, DP), along with the least-squares-fit of the enthalpy change per mole of guest (ΔH) to a 1:1 binding isotherm.*

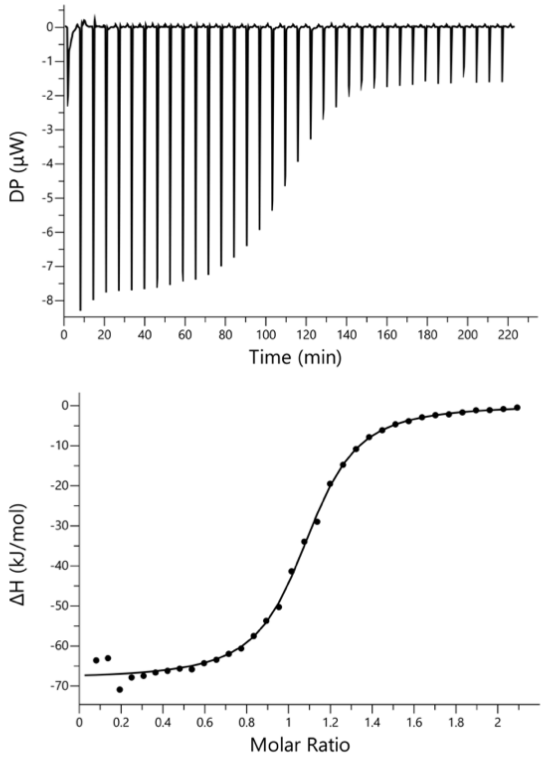

Figure S71. *ITC data for titration of* ***6*** *(0.30 mM) into* ***4*** *(0.03 mM) in chloroform at 298 K. The raw data for each injection is shown (differential power, DP), along with the least-squares-fit of the enthalpy change per mole of guest (ΔH) to a 1:1 binding isotherm.*

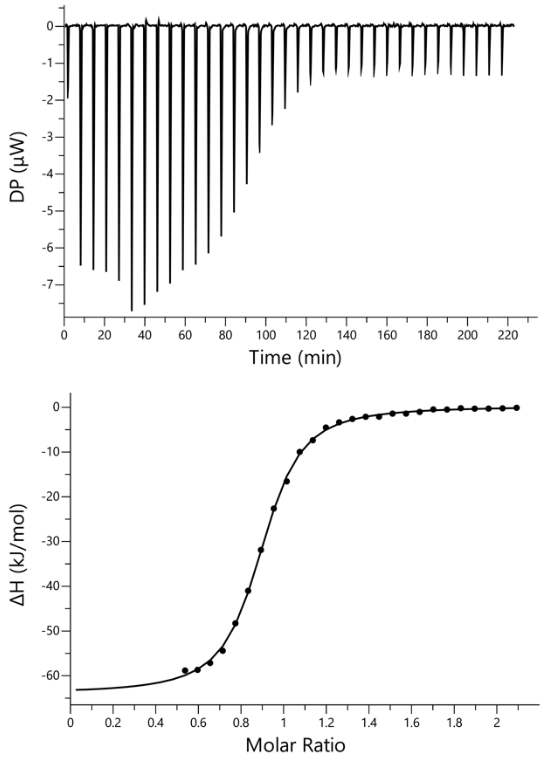

Figure S72. *ITC data for titration of* ***7*** *(0.30 mM) into* ***4*** *(0.03 mM) in chloroform at 298 K. The raw data for each injection is shown (differential power, DP), along with the least-squares-fit of the enthalpy change per mole of guest (ΔH) to a 1:1 binding isotherm.*

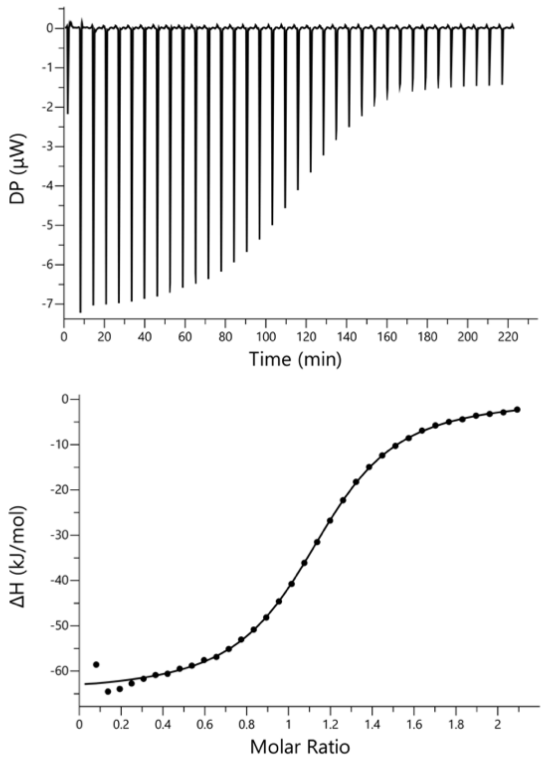

Figure S73. *ITC data for titration of* ***8*** *(0.30 mM) into* ***4*** *(0.03 mM) in chloroform at 298 K. The raw data for each injection is shown (differential power, DP), along with the least-squares-fit of the enthalpy change per mole of guest (ΔH) to a 1:1 binding isotherm.*

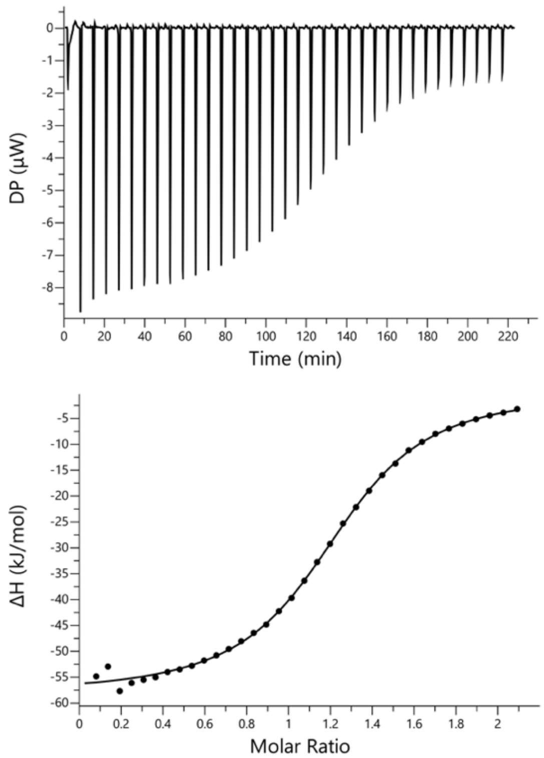

Figure S74. *ITC data for titration of* ***9*** *(0.30 mM) into* ***4*** *(0.03 mM) in chloroform at 298 K. The raw data for each injection is shown (differential power, DP), along with the least-squares-fit of the enthalpy change per mole of guest (ΔH) to a 1:1 binding isotherm.*

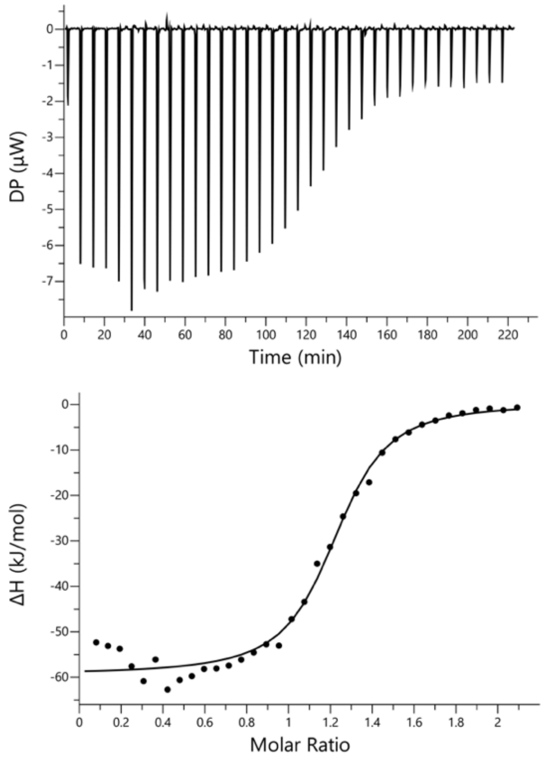

Figure S75. *ITC data for titration of **10** (0.30 mM) into **4** (0.03 mM) in chloroform at 298 K. The raw data for each injection is shown (differential power, DP), along with the least-squares-fit of the enthalpy change per mole of guest (ΔH) to a 1:1 binding isotherm.*

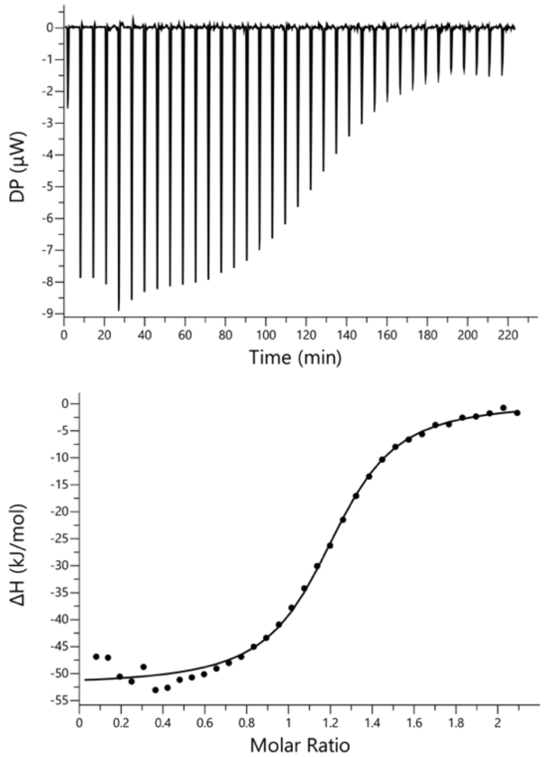

Figure S76. *ITC data for titration of **11** (0.30 mM) into **4** (0.03 mM) in chloroform at 298 K. The raw data for each injection is shown (differential power, DP), along with the least-squares-fit of the enthalpy change per mole of guest (ΔH) to a 1:1 binding isotherm.*

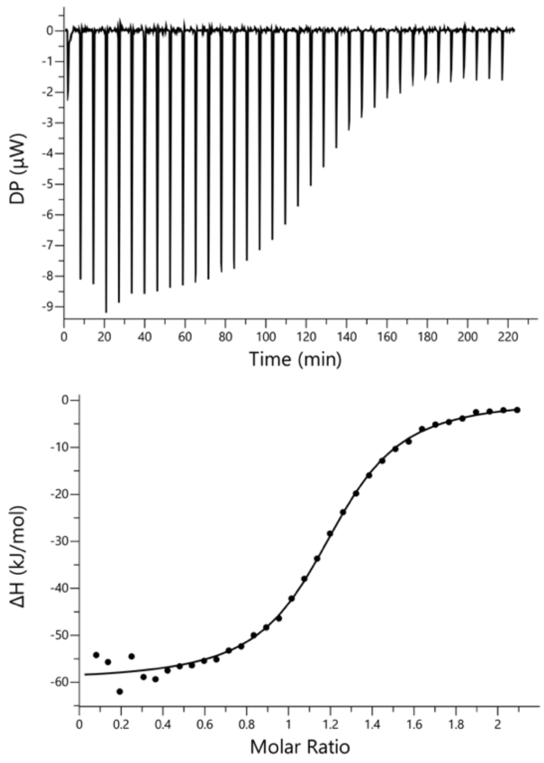

Figure S77. *ITC data for titration of **12** (0.30 mM) into **4** (0.03 mM) in chloroform at 298 K. The raw data for each injection is shown (differential power, DP), along with the least-squares-fit of the enthalpy change per mole of guest (ΔH) to a 1:1 binding isotherm.*

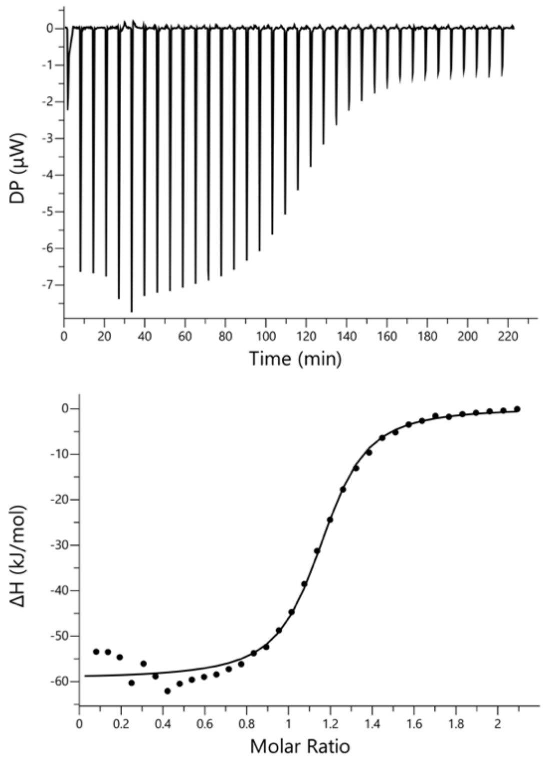

Figure S78. *ITC data for titration of **13** (0.30 mM) into **4** (0.03 mM) in chloroform at 298 K. The raw data for each injection is shown (differential power, DP), along with the least-squares-fit of the enthalpy change per mole of guest (ΔH) to a 1:1 binding isotherm.*

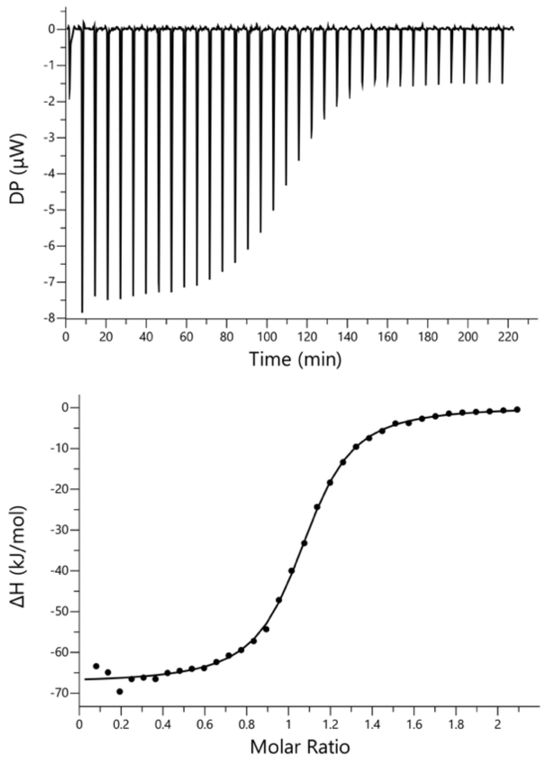

Figure S79. *ITC data for titration of **14** (0.30 mM) into **4** (0.03 mM) in chloroform at 298 K. The raw data for each injection is shown (differential power, DP), along with the least-squares-fit of the enthalpy change per mole of guest (ΔH) to a 1:1 binding isotherm.*

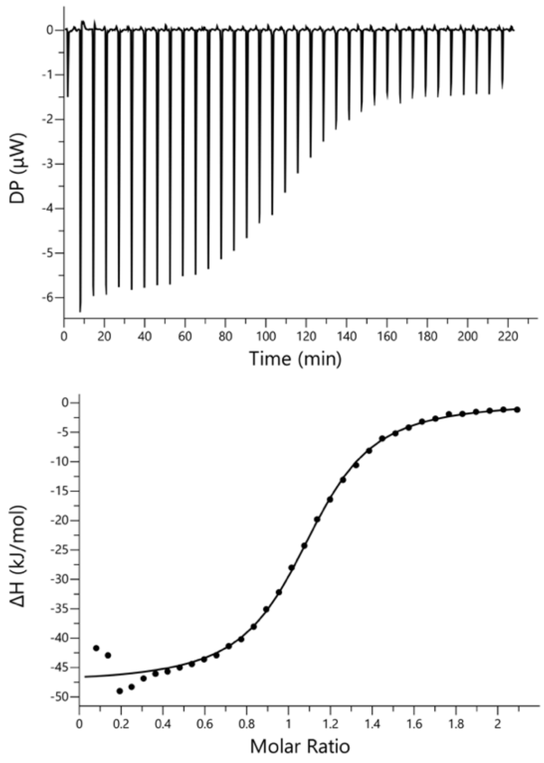

Figure S80. *ITC data for titration of **15** (0.30 mM) into **4** (0.03 mM) in chloroform at 298 K. The raw data for each injection is shown (differential power, DP), along with the least-squares-fit of the enthalpy change per mole of guest (ΔH) to a 1:1 binding isotherm.*

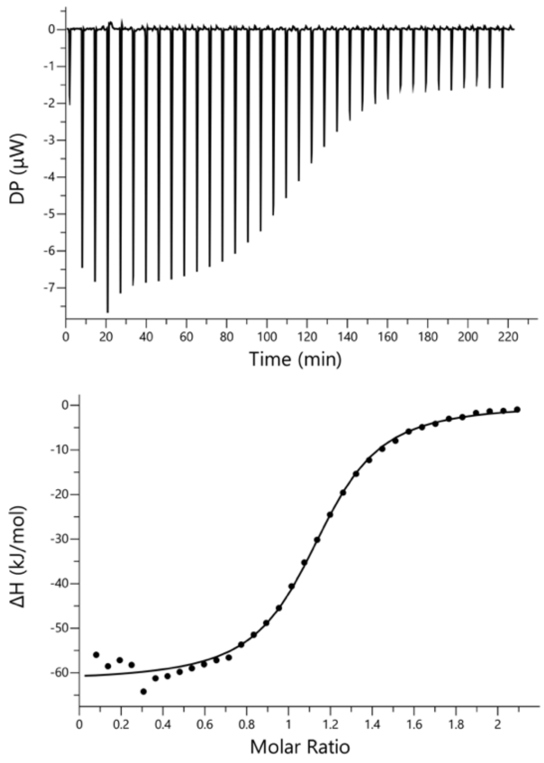

Figure S81. *ITC data for titration of* ***16*** *(0.30 mM) into* ***4*** *(0.03 mM) in chloroform at 298 K. The raw data for each injection is shown (differential power, DP), along with the least-squares-fit of the enthalpy change per mole of guest (ΔH) to a 1:1 binding isotherm.*

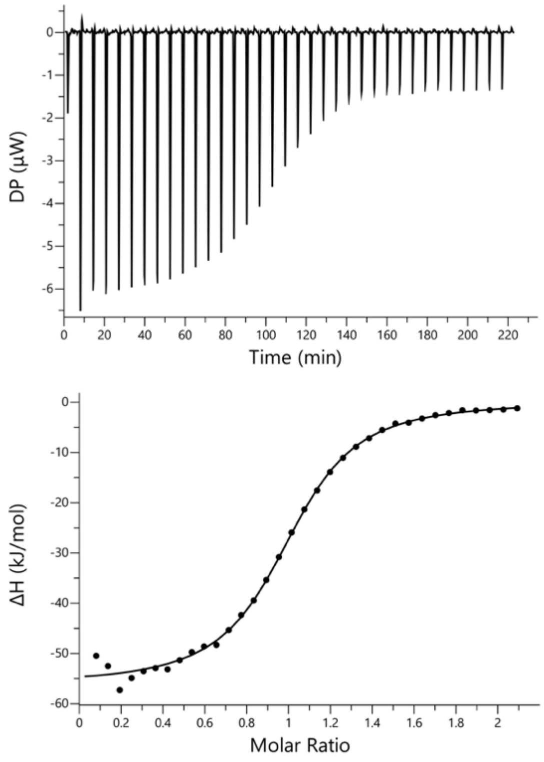

Figure S82. *ITC data for titration of* ***17*** *(0.30 mM) into* ***4*** *(0.03 mM) in chloroform at 298 K. The raw data for each injection is shown (differential power, DP), along with the least-squares-fit of the enthalpy change per mole of guest (ΔH) to a 1:1 binding isotherm.*

4. Pairwise ^{1}H NMR competitive titrations

Competitive titration experiments were performed using calix[4]pyrroles **1** and **2**, and pyridine *N*-oxides **5-17** and **PNO** in non-buffered deuterium oxide and deuterochloroform solutions. The association constant ratios between two competing complexes were determined by integrating selected proton signals in the acquired ^{1}H NMR spectra.

4.1. Octapyridinium-super-aryl-extended calix[4]pyrrole 1

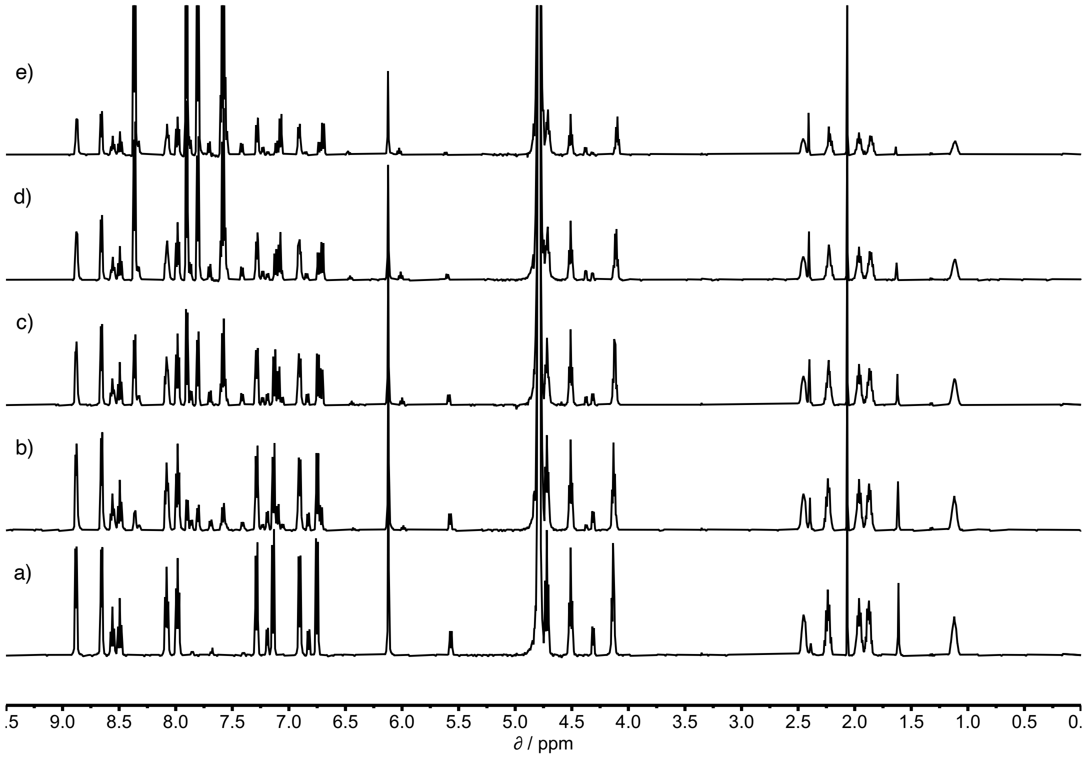

Figure S83. *500MHz ^{1}H NMR for titration of* ***5*** *into a mixture of* ***1*** *and* ***6*** *in D_2O at 298K. Concentrations are: a)* ***1****: 0.21 mM;* ***6****: 0.25 mM;* ***5****: 0 mM; b)* ***1****: 0.20 mM;* ***6****: 0.23 mM;* ***5****: 0.22 mM; c)* ***1****: 0.17 mM;* ***6****: 0.20 mM;* ***5****: 0.57 mM; d)* ***1****: 0.13 mM;* ***6****: 0.16 mM;* ***5****: 1.05 mM; e)* ***1****: 0.09 mM;* ***6****: 0.11 mM;* ***5****: 1.59 mM. Integration of selected proton signals indicated that K(**1•6**) = 7.9±0.6 x K(**1•5**).*

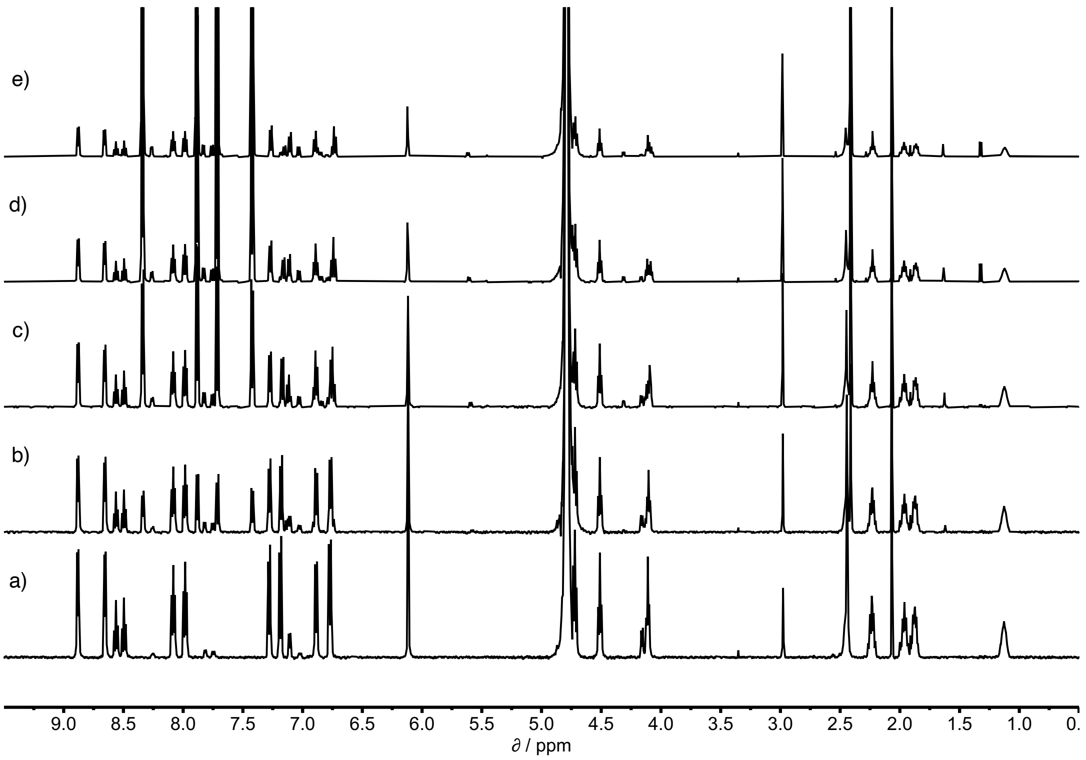

Figure S84. *500MHz ¹H NMR for titration of **6** into a mixture of **1** and **7** in D_2O at 298K. Concentrations are: a) **1**: 0.14 mM; **7**: 0.17 mM; **6**: 0 mM; b) **1**: 0.12 mM; **7**: 0.15 mM; **6**: 0.21 mM; c) **1**: 0.09mM; **7**: 0.11 mM; **6**: 0.49 mM; d) **1**: 0.07 mM; **7**: 0.08 mM; **6**: 0.79 mM; e) **1**: 0.05 mM; **7**: 0.05 mM; **6**: 1.00 mM. Integration of selected proton signals indicated that K(**1•7**) = 18.6±2.1 x K(**1•6**).*

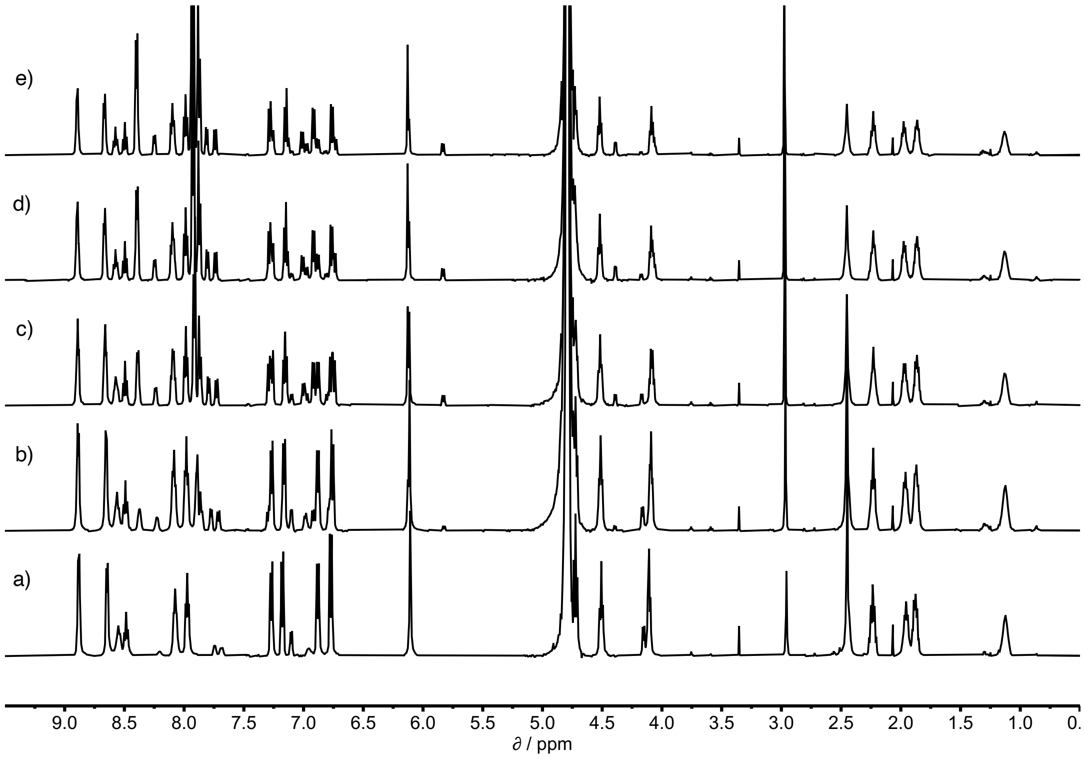

Figure S85. *500MHz ¹H NMR for titration of **8** into a mixture of **1** and **7** in D_2O at 298K. Concentrations are: a) **1**: 0.49 mM; **7**: 0.64 mM; **8**: 0 mM; b) **1**: 0.37 mM; **7**: 0.49 mM; **8**: 0.52 mM; c) **1**: 0.26 mM; **7**: 0.35 mM; **8**: 1.02 mM ; d) **1**: 0.17 mM; **7**: 0.23 mM; **8**: 1.42 mM; e) **1**: 0.13 mM; **7**: 0.15 mM; **8**: 1.70 mM. Integration of selected proton signals indicated that K(**1•7**) = 2.0±0.1 x K(**1•8**).*

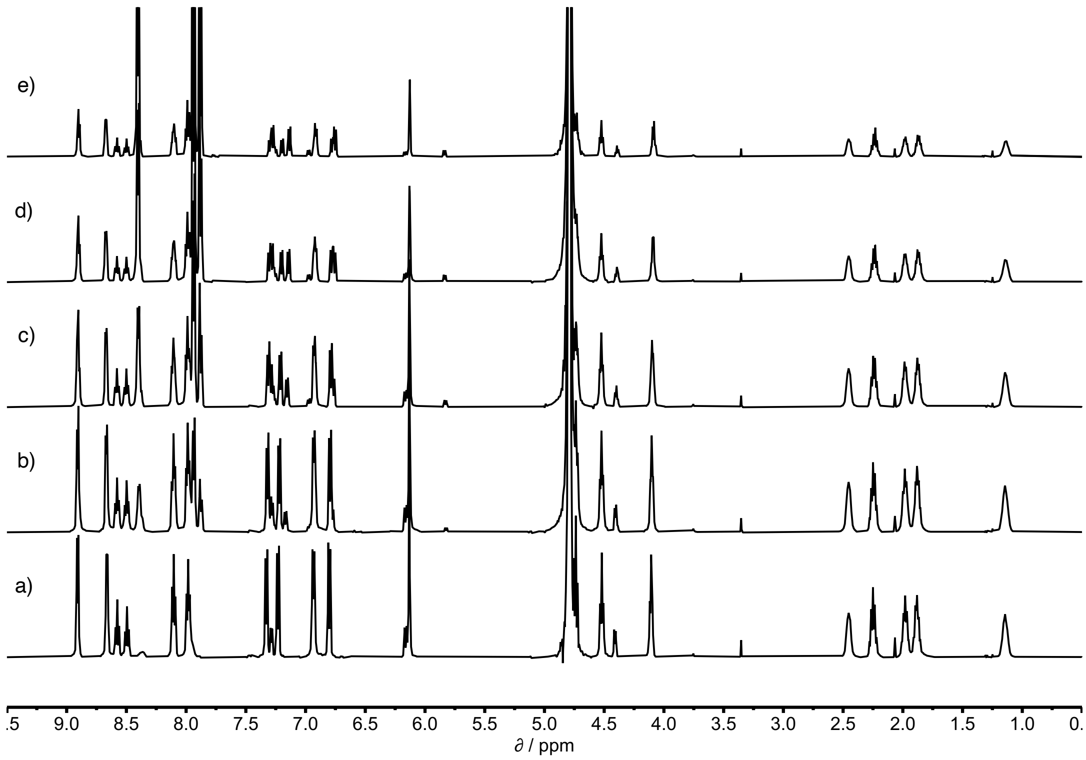

Figure S86. *500MHz ¹H NMR for titration of **8** into a mixture of **1** and **9** in D_2O at 298K. Concentrations are: a) **1**: 0.49 mM; **9**: 0.64 mM; **8**: 0 mM; b) **1**: 0.37 mM; **9**: 0.49 mM; **8**: 0.44 mM; c) **1**: 0.28 mM; **9**: 0.36 mM; **8**: 0.82 mM; d) **1**: 0.22 mM; **9**: 0.29 mM; **8**: 1.04 mM; e) **1**: 0.18 mM; **9**: 0.24 mM; **8**: 1.18 mM. Integration of selected proton signals indicated that K(**1•9**) = 14.6±0.5 x K(**1•8**).*

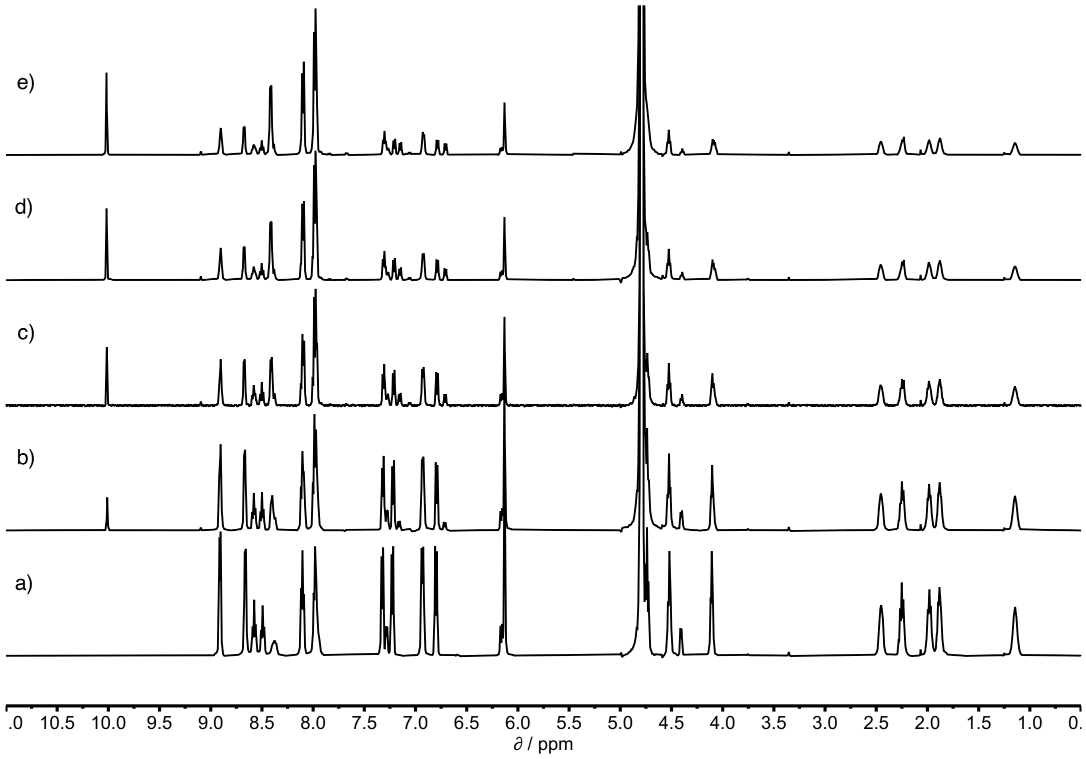

Figure S87. *500MHz ¹H NMR for titration of **11** into a mixture of **1** and **9** in D_2O at 298K. Concentrations are: a) **1**: 0.53 mM; **9**: 0.73 mM; **11**: 0 mM; b) **1**: 0.37 mM; **9**: 0.50 mM; **11**: 0.47 mM; c) **1**: 0.24 mM; **9**: 0.33 mM; **11**: 0.81 mM; d) **1**: 0.18 mM; **9**: 0.24 mM; **11**: 0.98 mM; e) **1**: 0.14 mM; **9**: 0.19 mM; **11**:1.08 mM. Integration of selected proton signals indicated that K(**1•9**) = 12.3±1.9 x K(**1•11**).*

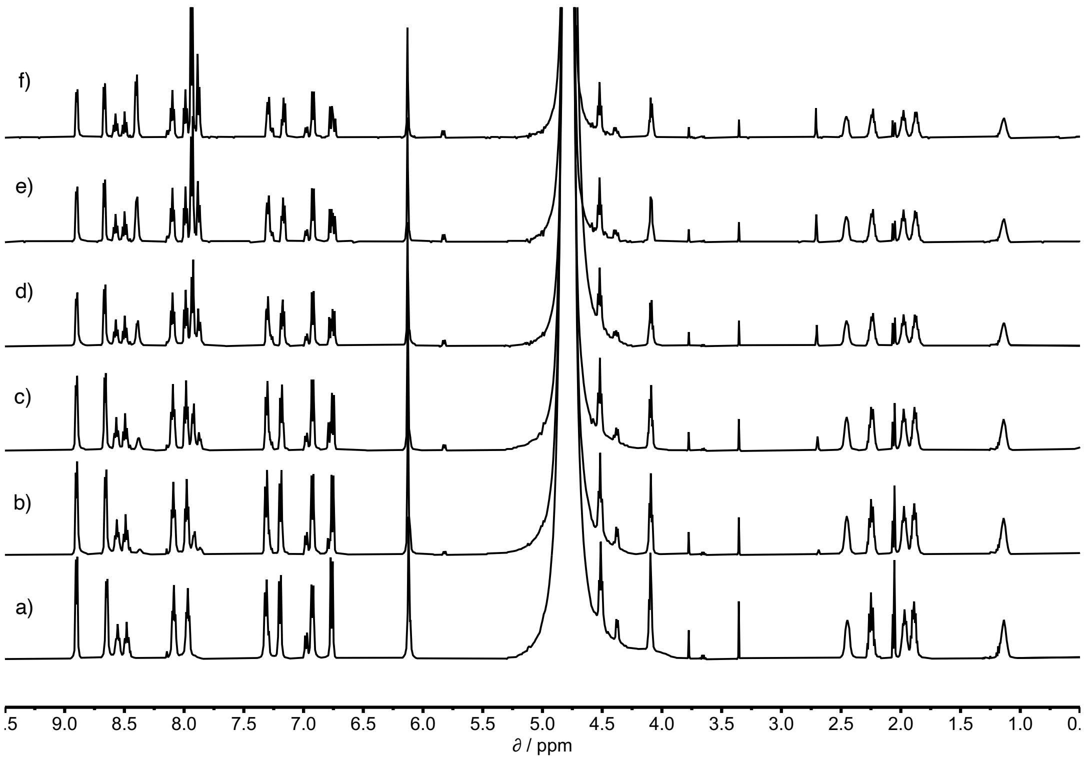

Figure S88. *500MHz ^{1}H NMR for titration of* ***8*** *into a mixture of* ***1*** *and* ***12*** *in D_2O at 298K. Concentrations are: a)* ***1****: 0.58 mM;* ***12****: 0.79 mM;* ***8****: 0 mM; b)* ***1****: 0.41 mM;* ***12****: 0.56 mM;* ***8****: 0.36 mM; c)* ***1****: 0.32 mM;* ***12****: 0.42 mM;* ***8****: 0.56 mM; d)* ***1****: 0.22 mM;* ***12****: 0.30 mM;* ***8****: 0.76 mM; e)* ***1****: 0.17 mM;* ***12****: 0.23 mM;* ***8****: 0.87 mM; f)* ***1****: 0.11 mM;* ***12****: 0.15 mM;* ***8****: 0.98 mM. Integration of selected proton signals indicated that K(****1•12****) = 6.3±1.8 x K(****1•8****).*

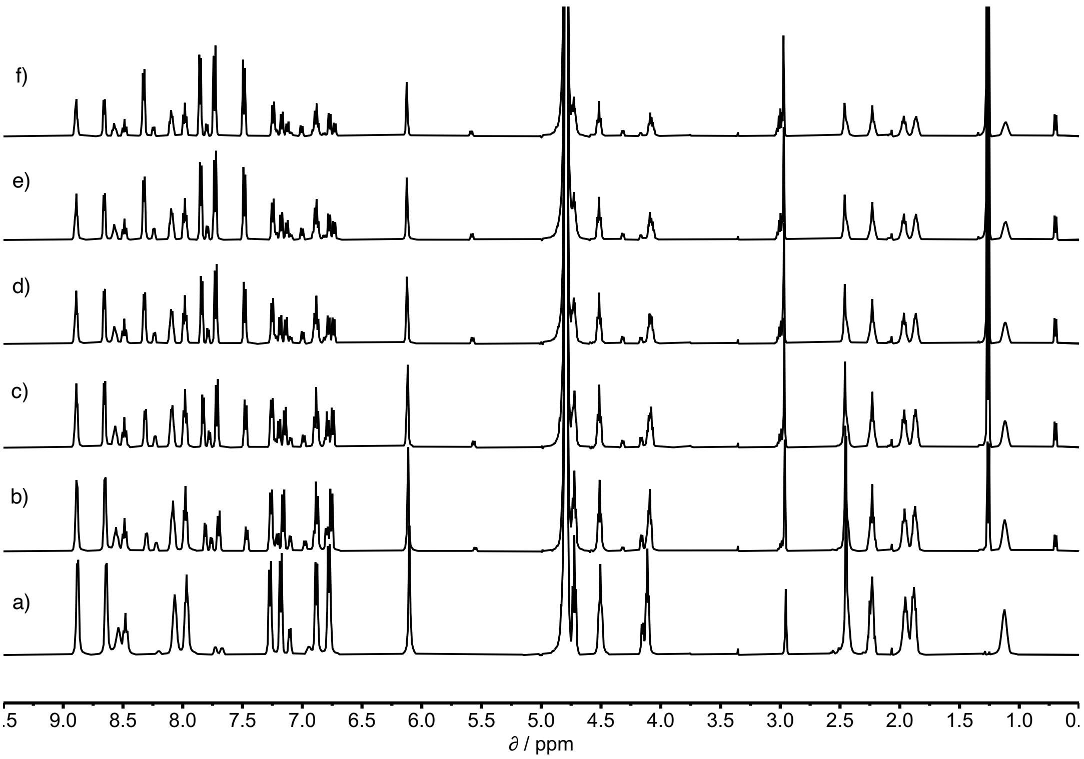

Figure S89. *500MHz ^{1}H NMR for titration of* ***13*** *into a mixture of* ***1*** *and* ***7*** *in D_2O at 298K. Concentrations are: a)* ***1****: 0.58 mM;* ***7****: 0.71 mM;* ***13****: 0 mM; b)* ***1****: 0.41 mM;* ***7****: 0.50 mM;* ***13****: 0.51 mM; c)* ***1****: 0.32 mM;* ***7****: 0.39 mM;* ***13****: 0.79 mM; d)* ***1****: 0.26 mM;* ***7****: 0.32 mM;* ***13****: 0.97 mM; e)* ***1****: 0.22 mM;* ***7****: 0.27 mM;* ***13****: 1.09 mM; f)* ***1****: 0.19 mM;* ***7****: 0.23 mM;* ***13****: 1.18 mM. Integration of selected proton signals indicated that K(****1•7****) = 4.2±1.0 x K(****1•13****)*

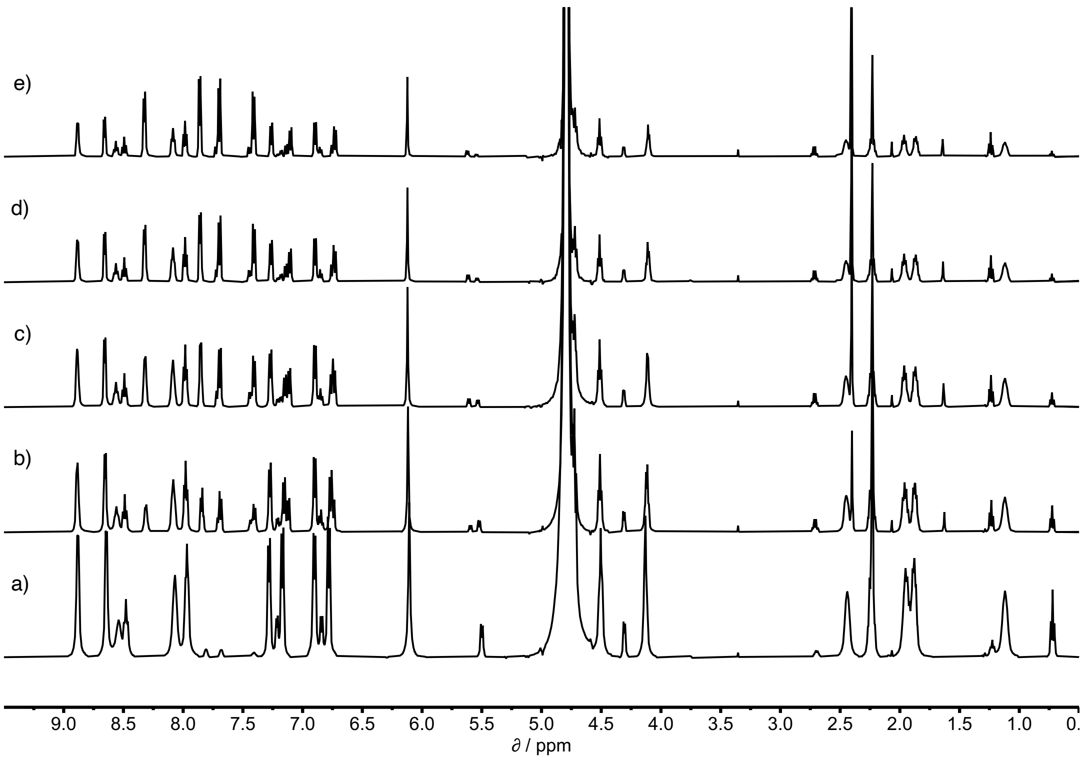

Figure S90. *500MHz* ^{1}H *NMR for titration of **6** into a mixture of **1** and **14** in* D_2O *at 298K. Concentrations are: a) **1**: 0.58 mM; **14**: 0.67 mM; **6**: 0 mM; b) **1**: 0.41 mM; **14**: 0.47 mM; **6**: 0.55 mM; c) **1**: 0.32 mM; **14**: 0.37 mM; **6**: 0.85 mM; d) **1**: 0.26 mM; **14**: 0.30 mM; **6**: 1.04 mM; e) **1**: 0.22 mM; **14**: 0.25 mM; **6**: 1.04 mM. Integration of selected proton signals indicated that K(**1•14**) = 2.3±0.3 x K(**1•6**).*

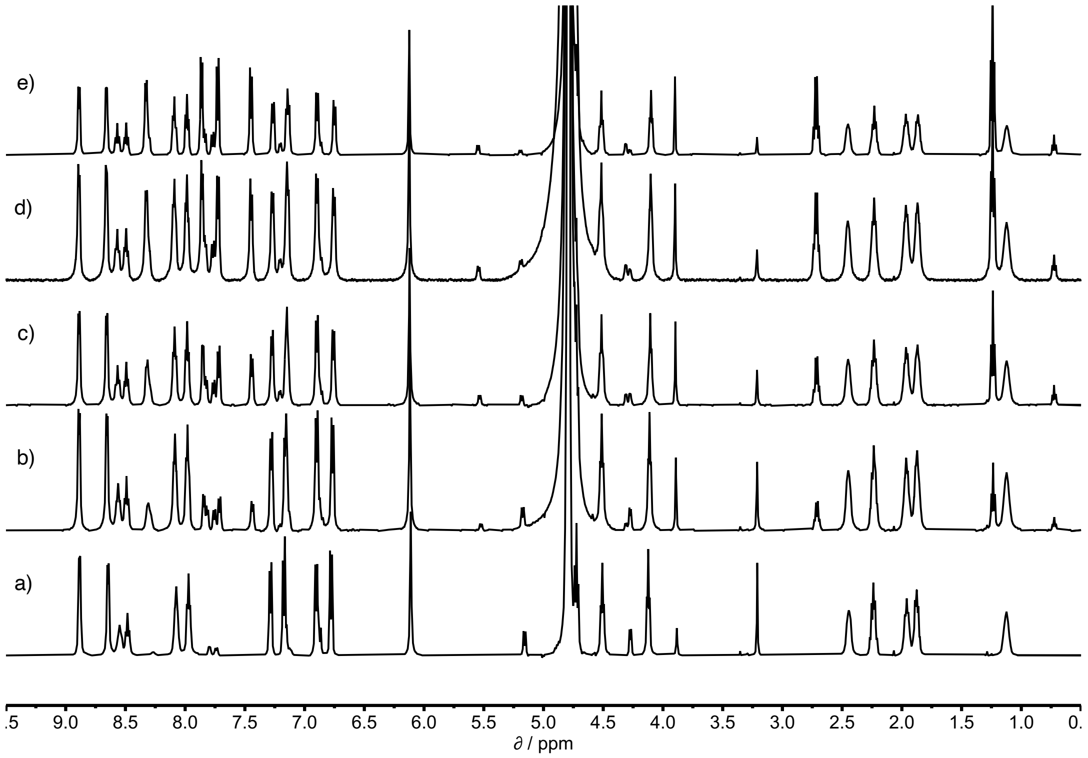

Figure S91. *500MHz* ^{1}H *NMR for titration of **14** into a mixture of **1** and **10** in* D_2O *at 298K. Concentrations are: a) **1**: 0.58 mM; **14**: 0 mM; **10**: 0.68 mM; b) **1**: 0.41 mM; **14**: 0.47 mM; **10**: 0.48 mM; c) **1**: 0.32 mM; **14**: 0.73 mM; **10**: 0.37 mM; d) **1**: 0.26 mM; **14**: 0.89 mM; **10**: 0.30 mM; e) **1**: 0.22 mM; **14**: 1.00 mM; **10**: 0.26 mM. Integration of selected proton signals indicated that K(**1•10**) = 2.4±0.4 x K(**1•14**).*

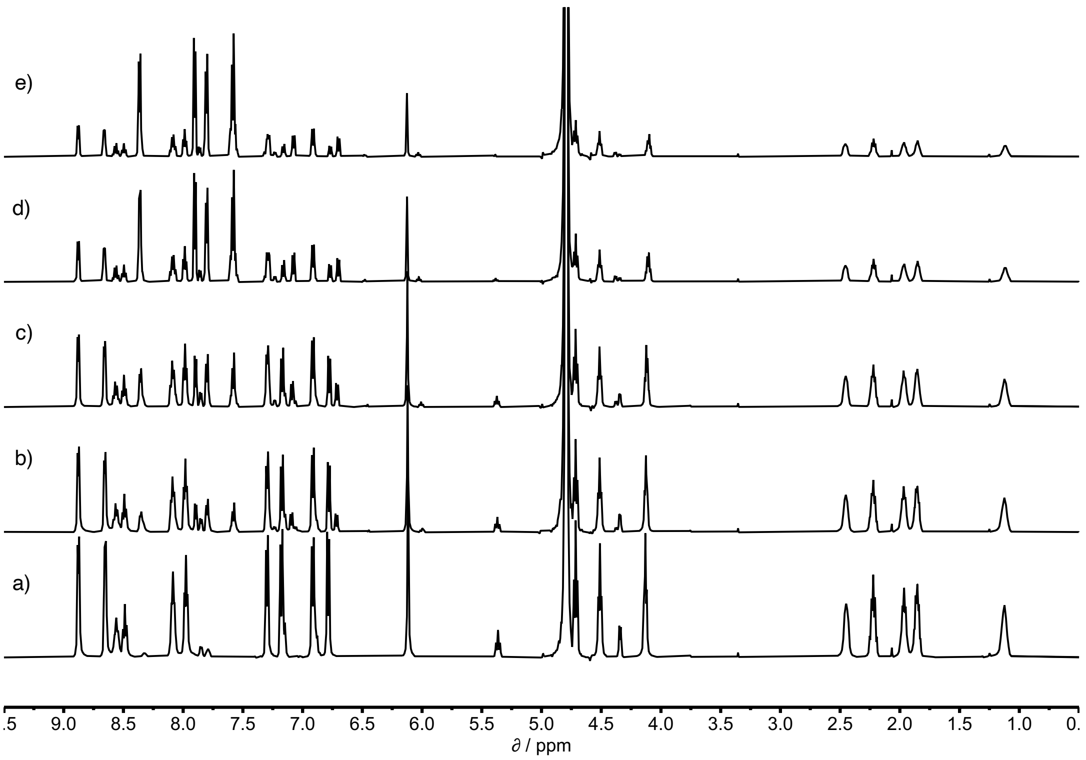

Figure S92. *500MHz ^{1}H NMR for titration of **5** into a mixture of **1** and **15** in D_2O at 298K. Concentrations are: a) **1**: 0.56 mM; **5**: 0 mM; **15**: 0.63 mM; b) **1**: 0.45 mM; **5**: 0.51 mM; **15**: 0.50 mM; c) **1**: 0.38 mM; **5**: 0.85 mM; **15**: 0.42 mM; d) **1**: 0.21 mM; **5**: 1.62 mM; **15**: 0.23 mM; e) **1**: 0.16 mM; **5**: 1.82 mM; **15**: 0.18 mM. Integration of selected proton signals indicated that K(**1•15**) = 8.4±1.5 x K(**1•5**).*

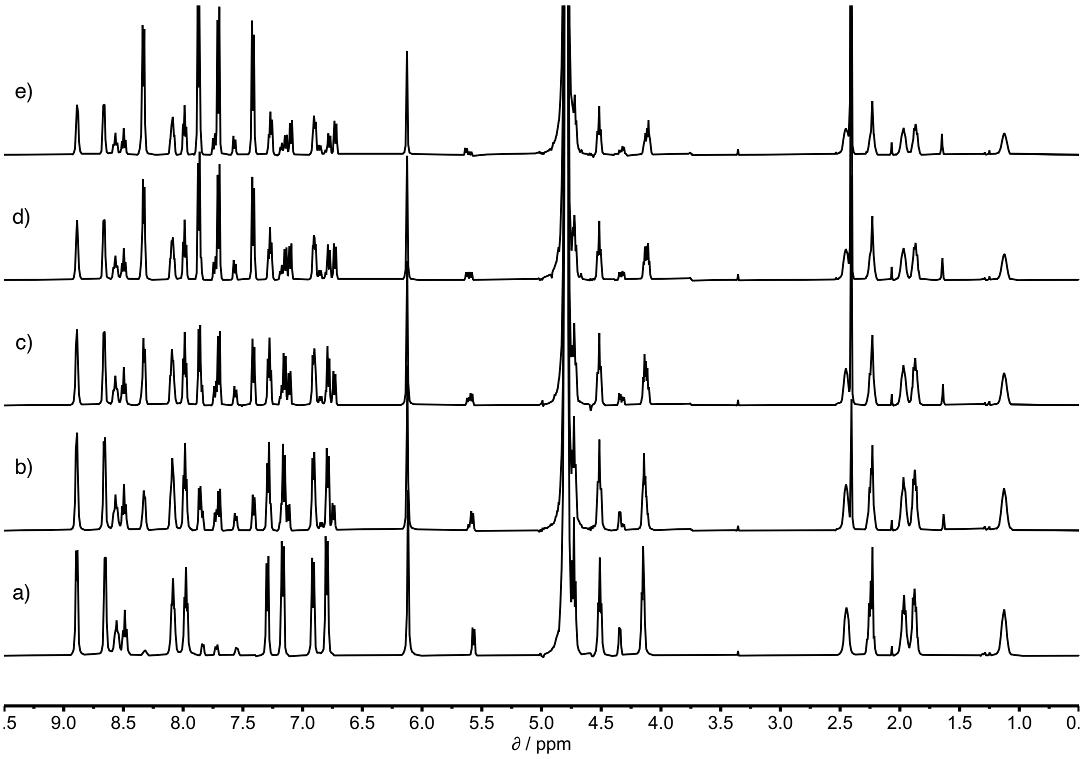

Figure S93. *500MHz ^{1}H NMR for titration of **6** into a mixture of **1** and **16** in D_2O at 298K. Concentrations are: a) **1**: 0.56 mM; **6**: 0 mM; **16**: 0.69 mM; b) **1**: 0.40 mM; **6**: 0.55 mM; **16**: 0.49 mM; c) **1**: 0.31 mM; **6**: 0.85 mM; **16**: 0.38 mM; d) **1**: 0.23 mM; **6**: 1.13 mM; **16**: 0.28 mM; e) **1**: 0.17 mM; **6**: 1.32 mM; **16**: 0.21 mM. Integration of selected proton signals indicated that K(**1•16**) = 5.1±1.4 x K(**1•6**).*

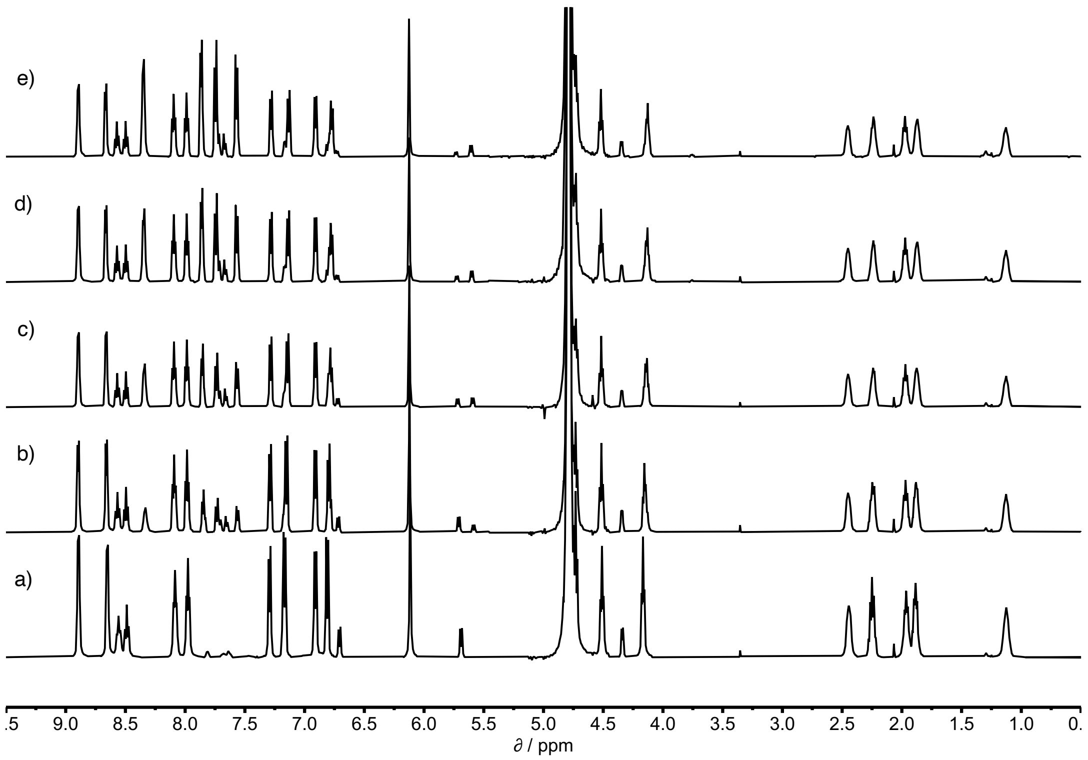

Figure S94. *500MHz ^{1}H NMR for titration of **16** into a mixture of **1** and **17** in D_2O at 298K. Concentrations are: a) **1**: 0.50 mM; **16**: 0 mM; **17**: 0.59 mM; b) **1**: 0.37 mM; **16**: 0.44 mM; **17**: 0.43 mM; c) **1**: 0.29 mM; **16**: 0.69 mM; **17**: 0.34 mM; d) **1**: 0.24 mM; **16**: 0.86 mM; **17**: 0.28 mM; e) **1**: 0.20 mM; **16**: 0.98 mM; **17**: 0.24 mM. Integration of selected proton signals indicated that K(**1•17**) = 1.8±0.1 x K(**1•16**).*

4.2. Octachloro-super-aryl-extended calix[4]pyrrole 2

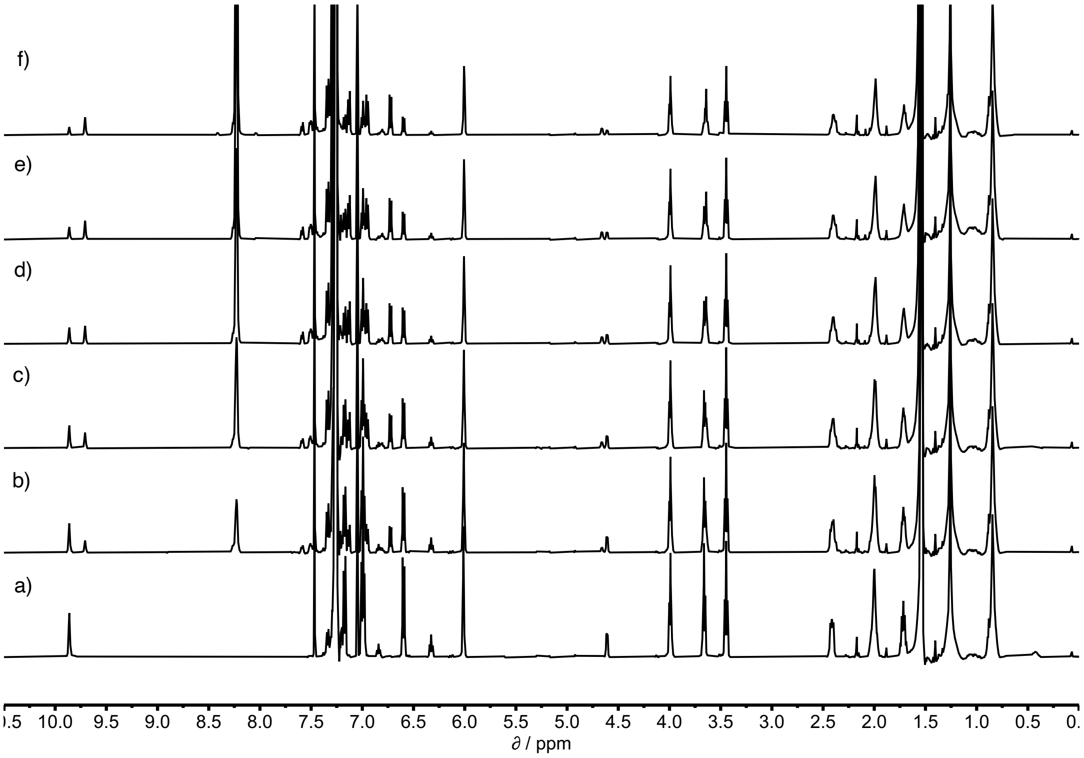

Figure S95. *500MHz ¹H NMR for titration of* ***PNO*** *into a mixture of* ***2*** *and* ***5*** *in $CDCl_3$ at 298K. Concentrations are: a)* ***2****: 0.13 mM;* ***PNO****: 0 mM;* ***5****: 0.18 mM; b)* ***2****: 0.12 mM;* ***PNO****: 0.53 mM;* ***5****: 0.16 mM; c)* ***2****: 0.11 mM;* ***PNO****: 0.99 mM;* ***5****: 0.15 mM; d)* ***2****: 0.10 mM;* ***PNO****: 1.62 mM;* ***5****: 0.14 mM; e)* ***2****: 0.09 mM;* ***PNO****: 2.13 mM;* ***5****: 0.12 mM; f)* ***2****: 0.08 mM;* ***PNO****: 2.90 mM;* ***5****: 0.10 mM. Integration of selected proton signals indicated that K(****2•5****) = 17.4±2.3 x K(****2•PNO****).*

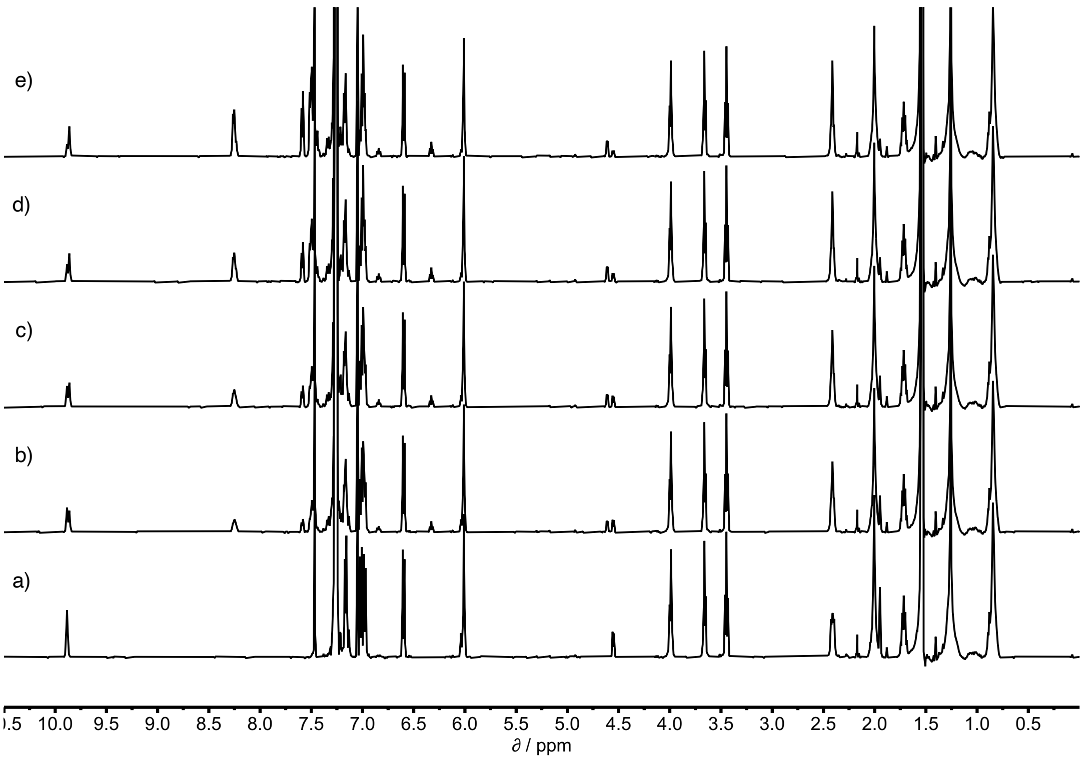

Figure S96. *500MHz* ^{1}*H NMR for titration of* ***5*** *into a mixture of* ***2*** *and* ***6*** *in* $CDCl_3$ *at 298K. Concentrations are: a)* ***2****: 0.13 mM;* ***5****: 0 mM;* ***6****: 0.16 mM; b)* ***2****: 0.12 mM;* ***5****: 0.17 mM;* ***6****: 0.16 mM; c)* ***2****: 0.12 mM;* ***5****: 0.22 mM;* ***6****: 0.16 mM; d)* ***2****: 0.12 mM;* ***5****: 0.32 mM;* ***6****: 0.15 mM; e)* ***2****: 0.12 mM;* ***5****: 0.47 mM;* ***6****: 0.15 mM. Integration of selected proton signals indicated that K(****2•6****) = 1.4±0.1 x K(****2•5****).*

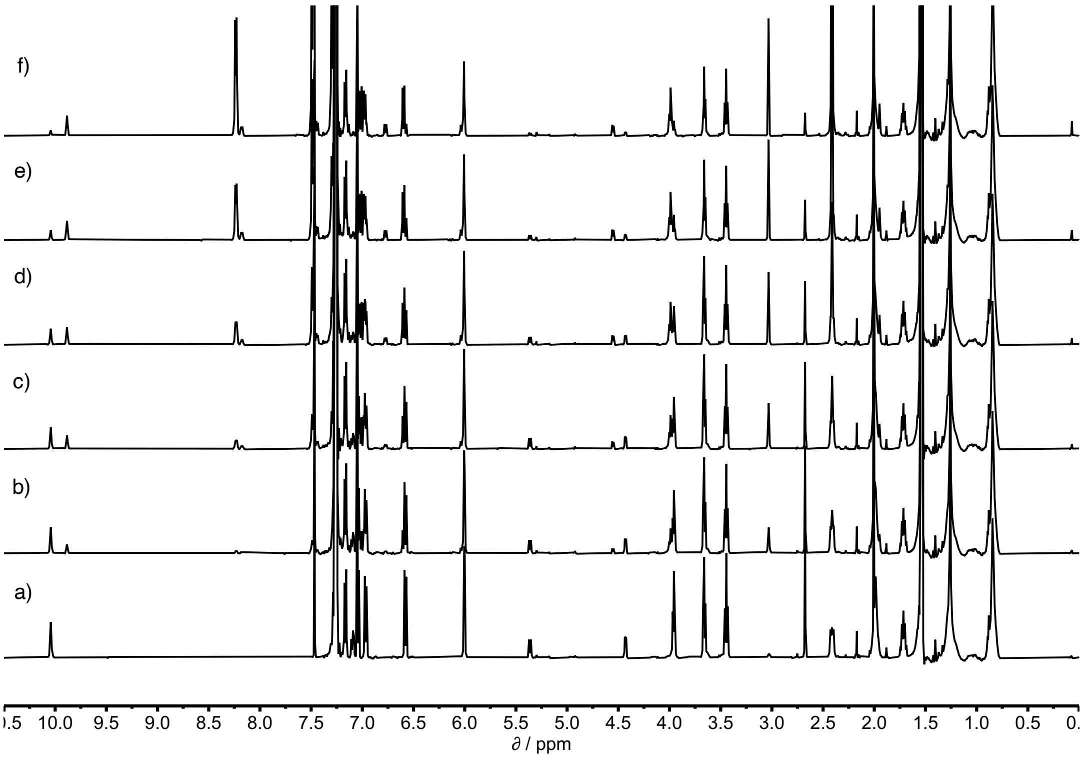

Figure S97. *500MHz* ^{1}*H NMR for titration of* ***6*** *into a mixture of* ***2*** *and* ***7*** *in* $CDCl_3$ *at 298K. Concentrations are: a)* ***2****: 0.13 mM;* ***6****: 0 mM;* ***7****: 0.15 mM; b)* ***2****: 0.12 mM;* ***6****: 0.08 mM;* ***7****: 0.15 mM; c)* ***2****: 0.12 mM;* ***6****: 0.25 mM;* ***7****: 0.15 mM; d)* ***2****: 0.12 mM;* ***6****: 0.29 mM;* ***7****: 0.14 mM; e)* ***2****: 0.11 mM;* ***6****: 0.53 mM;* ***7****: 0.13 mM; f)* ***2****: 0.09 mM;* ***6****: 0.88 mM;* ***7****: 0.11 mM. Integration of selected proton signals indicated that K(****2•7****) = 2.5±0.1 x K(****2•6****).*

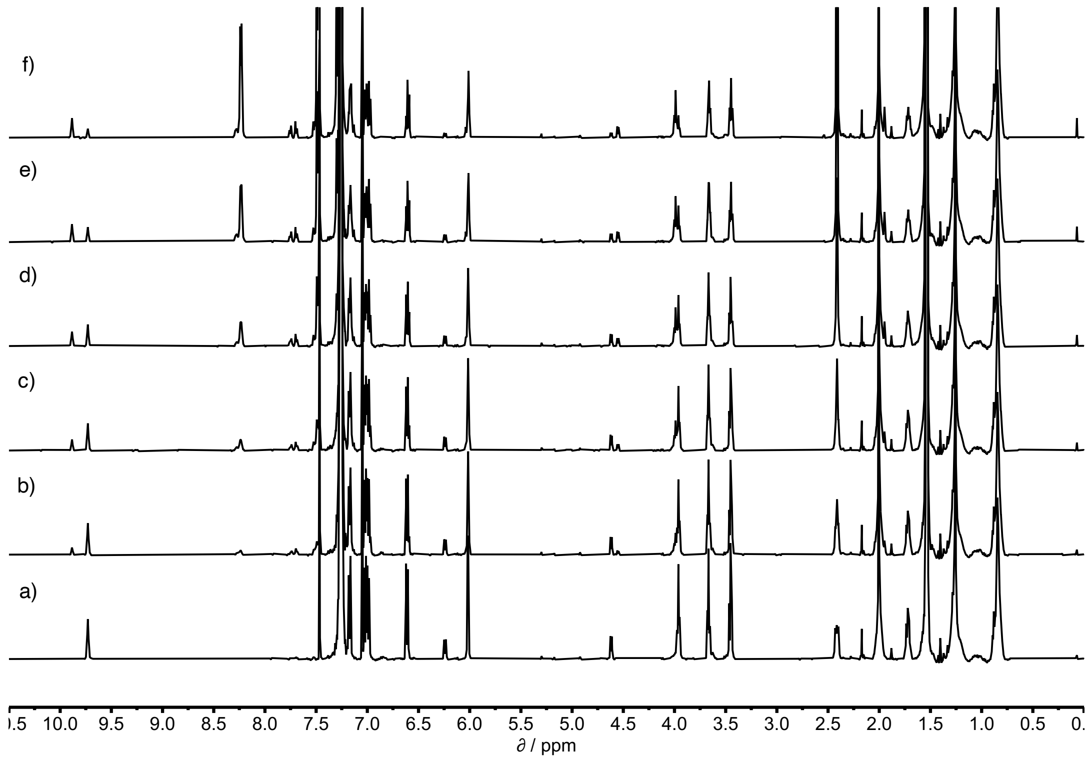

Figure S98. *500MHz ¹H NMR for titration of **6** into a mixture of **2** and **8** in $CDCl_3$ at 298K. Concentrations are: a) **2**: 0.13 mM; **6**: 0 mM; **8**: 0.15 mM; b) **2**: 0.12 mM; **6**: 0.08 mM; **8**: 0.15 mM; c) **2**: 0.12 mM; **6**: 0.25 mM; **8**: 0.15 mM; d) **2**: 0.11 mM; **6**: 0.29 mM; **8**: 0.14 mM; e) **2**: 0.10 mM; **6**: 0.52 mM; **8**: 0.13 mM; f) **2**: 0.09 mM; **6**: 0.87 mM; **8**: 0.11 mM. Integration of selected proton signals indicated that K(**2•8**) = 4.6±0.0 x K(**2•6**).*

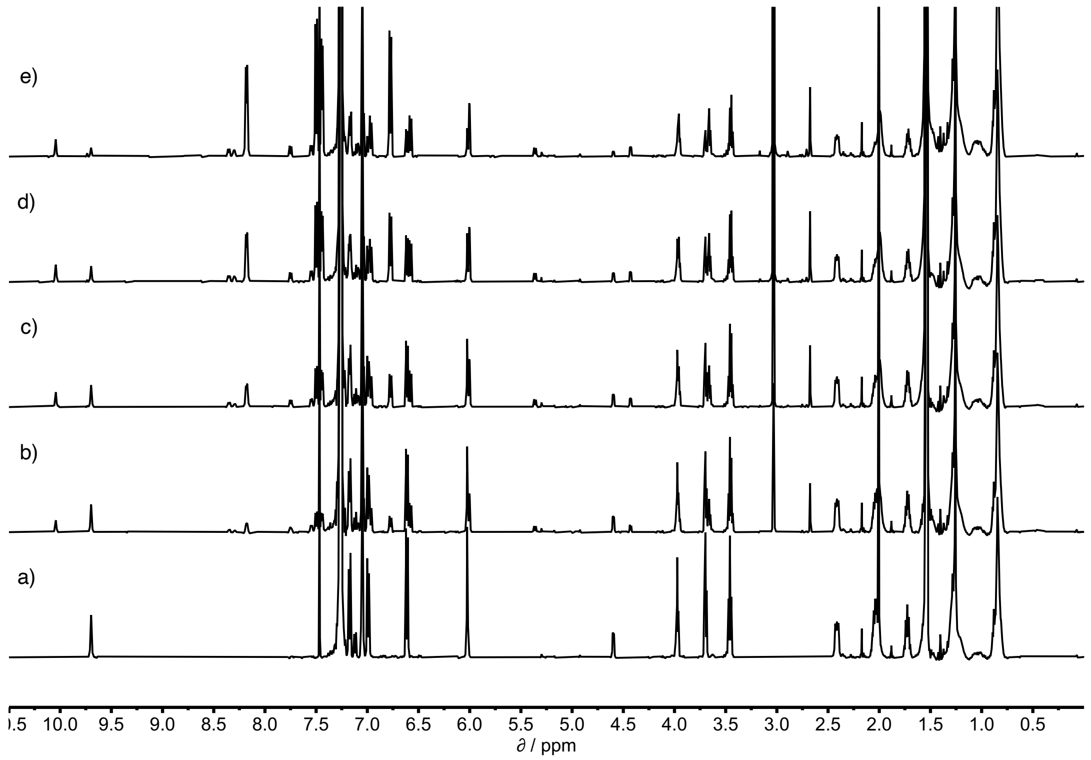

Figure S99. *500MHz ¹H NMR for titration of **7** into a mixture of **2** and **9** in $CDCl_3$ at 298K. Concentrations are: a) **2**: 0.13 mM; **7**: 0 mM; **9**: 0.15 mM; b) **2**: 0.12 mM; **7**: 0.16 mM; **9**: 0.14 mM; c) **2**: 0.11 mM; **7**: 0.28 mM; **9**: 0.13 mM; d) **2**: 0.10 mM; **7**: 0.47 mM; **9**: 0.11 mM; e) **2**: 0.08 mM; **7**: 0.75 mM; **9**: 0.09 mM. Integration of selected proton signals indicated that K(**2•9**) = 5.2±0.1 x K(**2•7**).*

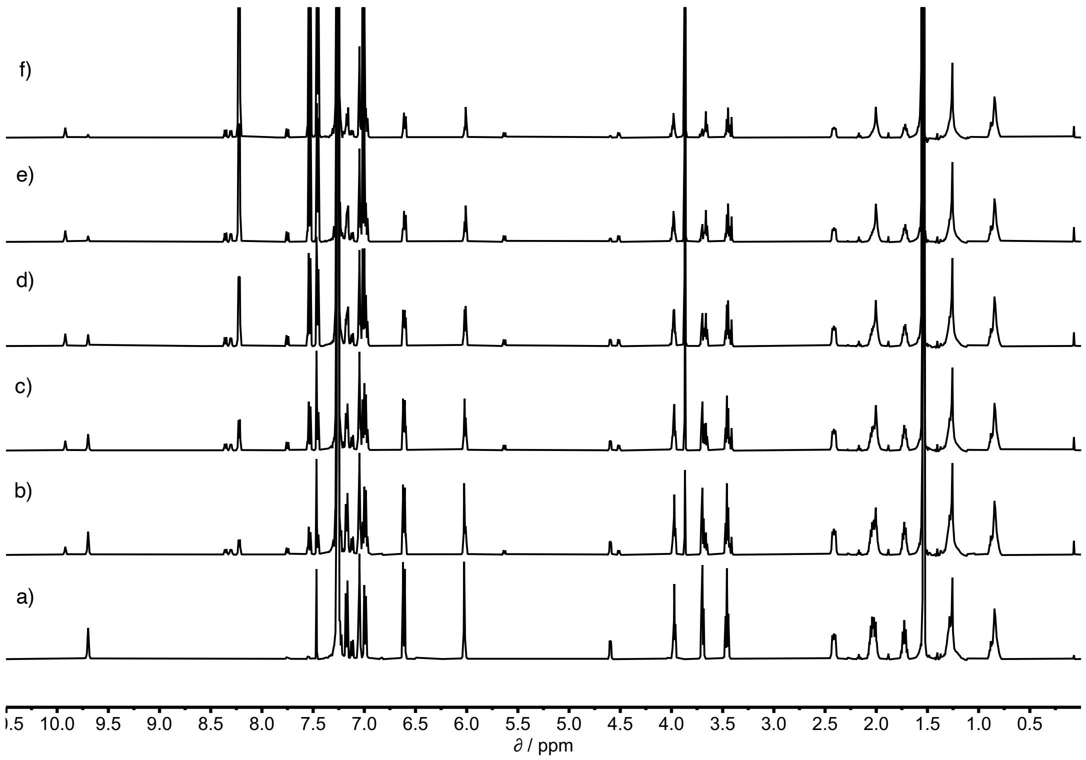

Figure S100. *500MHz* ${}^{1}H$ *NMR for titration of* ***10*** *into a mixture of* ***2*** *and* ***9*** *in* $CDCl_3$ *at 298K. Concentrations are: a)* ***2****: 0.20 mM;* ***10****: 0 mM;* ***9****: 0.26 mM; b)* ***2****: 0.18 mM;* ***10****: 0.23 mM;* ***9****: 0.23 mM; c)* ***2****: 0.17 mM;* ***10****: 0.42 mM;* ***9****: 0.21 mM; d)* ***2****: 0.14 mM;* ***10****: 0.73 mM;* ***9****: 0.18 mM; e)* ***2****: 0.11 mM;* ***10****: 1.14 mM;* ***9****: 0.14 mM; f)* ***2****: 0.08 mM;* ***10****: 1.51 mM;* ***9****: 0.10 mM. Integration of selected proton signals indicated that K(****2•9****) = 5.3±0.1 x K(****2•10****).*

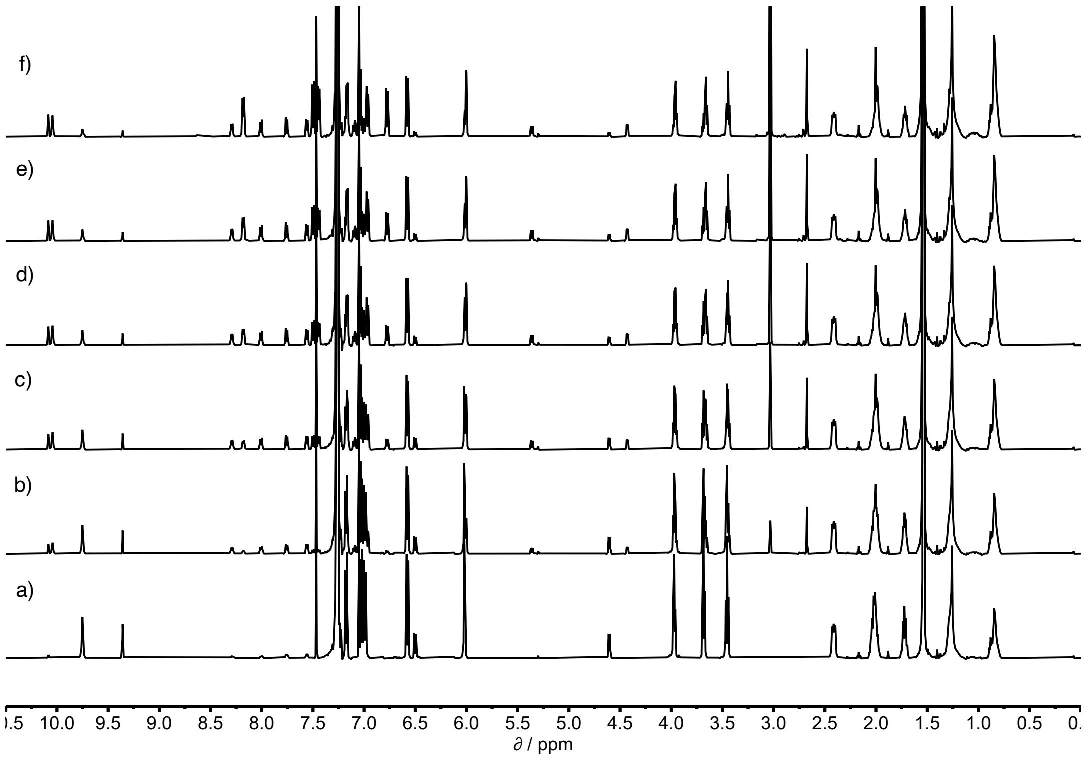

Figure S101. *500MHz* ${}^{1}H$ *NMR for titration of* ***7*** *into a mixture of* ***2*** *and* ***11*** *in* $CDCl_3$ *at 298K. Concentrations are: a)* ***2****: 0.20 mM;* ***7****: 0 mM;* ***11****: 0.26 mM; b)* ***2****: 0.19 mM;* ***7****: 0.11 mM;* ***11****: 0.25 mM; c)* ***2****: 0.18 mM;* ***7****: 0.23 mM;* ***11****: 0.23 mM; d)* ***2****: 0.17 mM;* ***7****: 0.33 mM;* ***11****: 0.22 mM; e)* ***2****: 0.16 mM;* ***7****: 0.42 mM;* ***11****: 0.21 mM; f)* ***2****: 0.14 mM;* ***7****: 0.59 mM;* ***11****: 0.19 mM. Integration of selected proton signals indicated that K(****2•11****) = 1.2±0.1 x K(****2•7****).*

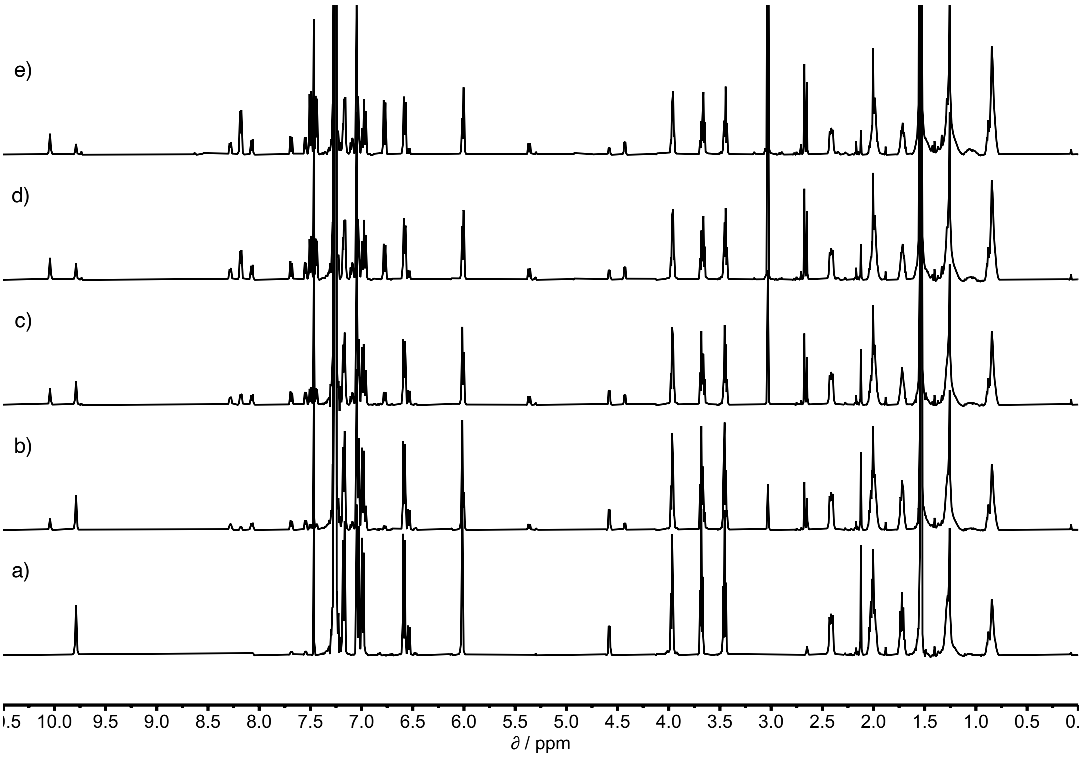

Figure S102. *500MHz ¹H NMR for titration of **7** into a mixture of **2** and **12** in $CDCl_3$ at 298K. Concentrations are: a) **2**: 0.21 mM; **7**: 0 mM; **12**: 0.27 mM; b) **2**: 0.20 mM; **7**: 0.11 mM; **12**: 0.25 mM; c) **2**: 0.18 mM; **7**: 0.23 mM; **12**: 0.23 mM; d) **2**: 0.16 mM; **7**: 0.43 mM; **12**: 0.21 mM; e) **2**: 0.14 mM; **7**: 0.59 mM; **12**: 0.19 mM. Integration of selected proton signals indicated that K(**2•12**) = 1.8±0.1 x K(**2•7**).*

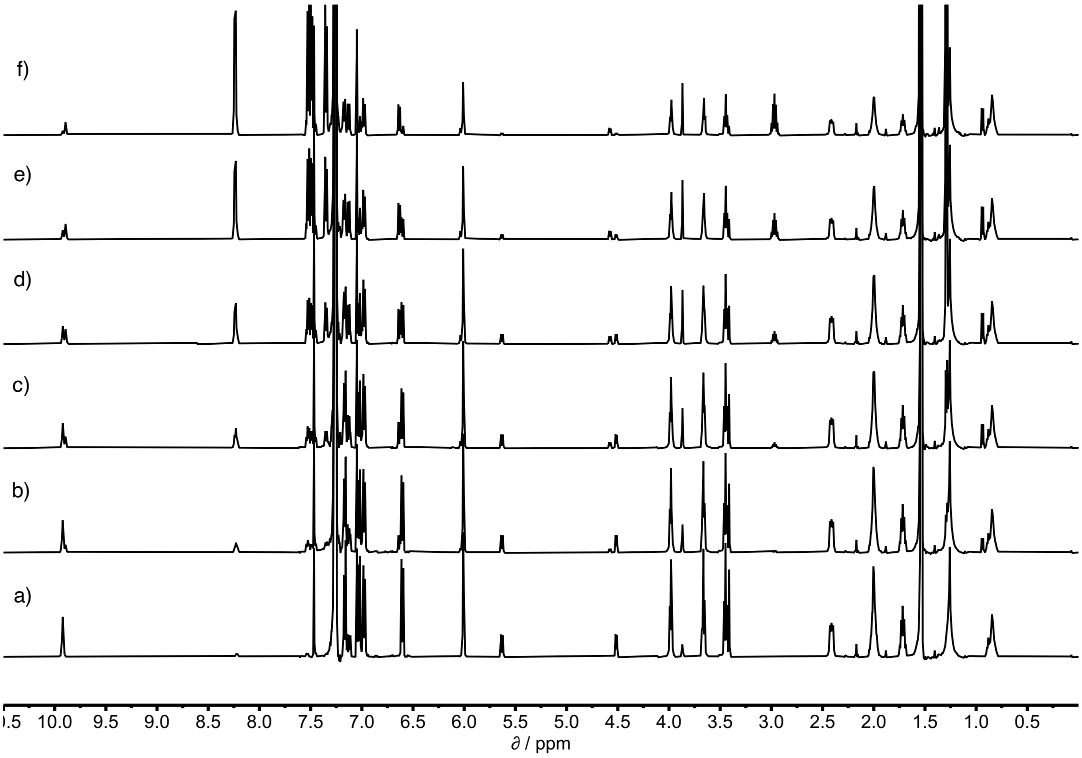

Figure S103. *500MHz ¹H NMR for titration of **13** into a mixture of **2** and **10** in $CDCl_3$ at 298K. Concentrations are: a) **2**: 0.20 mM; **13**: 0 mM; **10**: 0.26 mM; b) **2**: 0.19 mM; **13**: 0.10 mM; **10**: 0.24 mM; c) **2**: 0.18 mM; **13**: 0.22 mM; **10**: 0.23 mM; d) **2**: 0.16 mM; **13**: 0.41 mM; **10**: 0.20 mM; e) **2**: 0.13 mM; **13**: 0.68 mM; **10**: 0.16 mM; f) **2**: 0.09 mM; **13**: 1.00 mM; **10**: 0.12 mM. Integration of selected proton signals indicated that K(**2•10**) = 3.3±0.6 x K(**2•13**).*

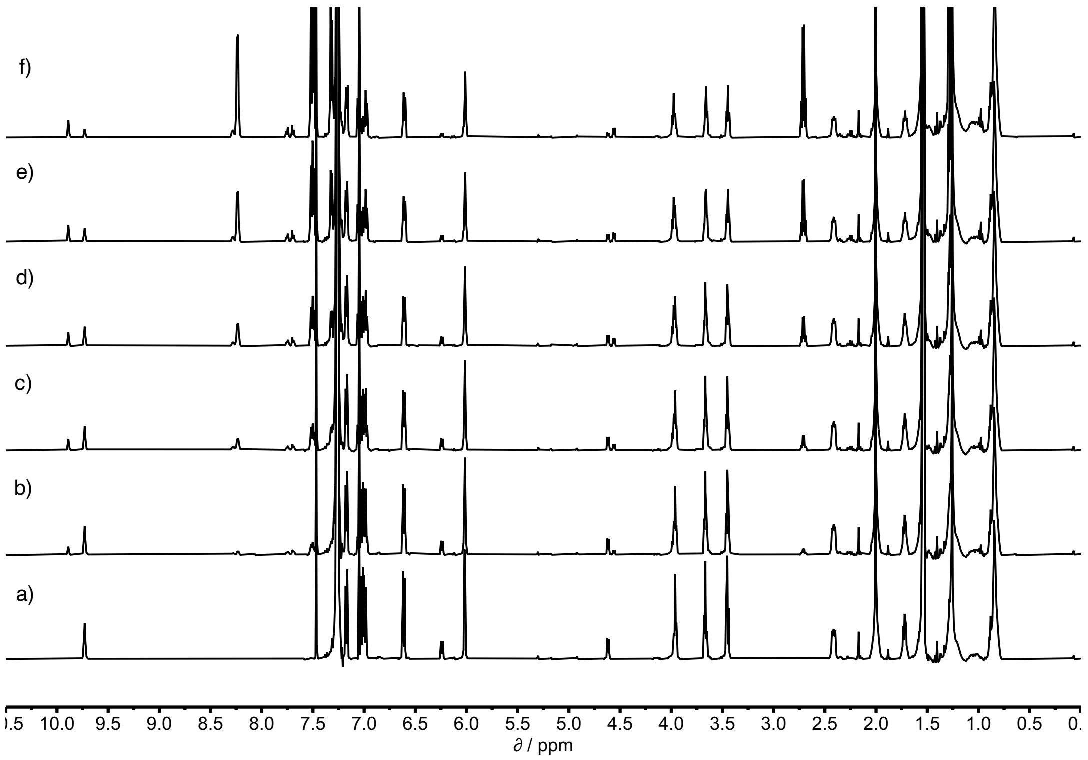

Figure S104. *500MHz* ^{1}*H NMR for titration of **14** into a mixture of **2** and **8** in* $CDCl_3$ *at 298K. Concentrations are: a) **2**: 0.13 mM; **14**: 0 mM; **8**: 0.15 mM; b) **2**: 0.12 mM; **14**: 0.09 mM; **8**: 0.15 mM; c) **2**: 0.12 mM; **14**: 0.17 mM; **8**: 0.14 mM; d) **2**: 0.11 mM; **14**: 0.28 mM; **8**: 0.14 mM; e) **2**: 0.10 mM; **14**: 0.50 mM; **8**: 0.12 mM; f) **2**: 0.08 mM; **14**: 0.81 mM; **8**: 0.08 mM. Integration of selected proton signals indicated that K(**2•8**) = 4.6±0.7 x K(**2•14**).*

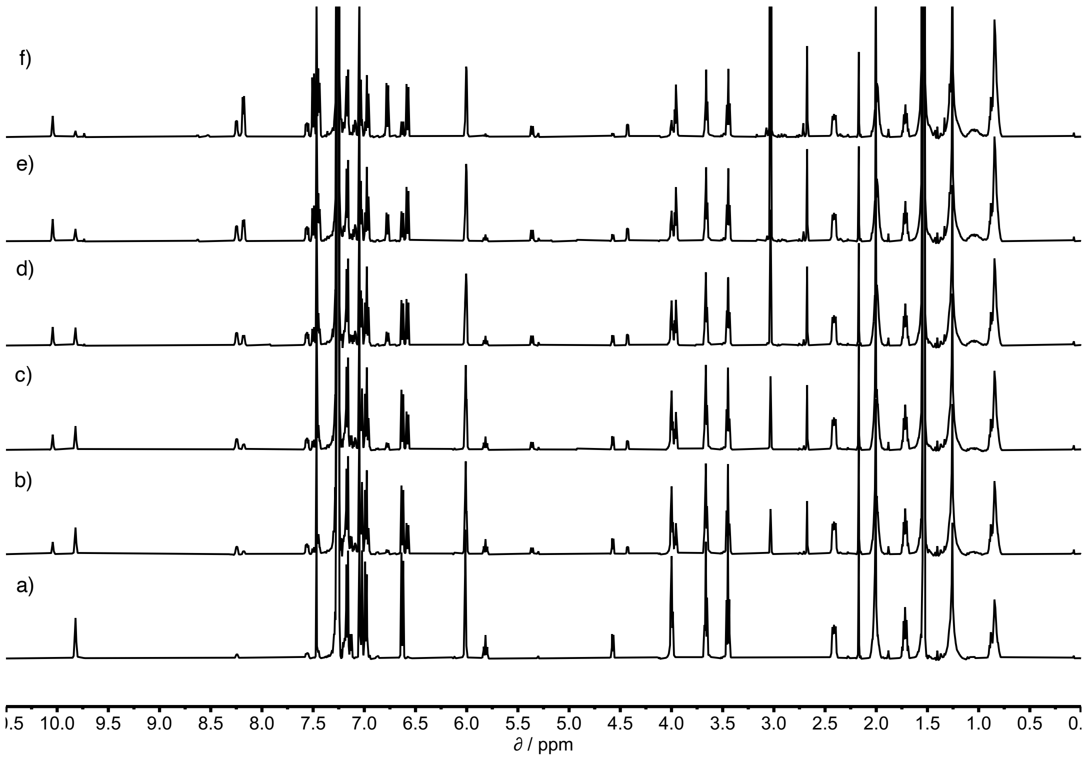

Figure S105. *500MHz* ^{1}*H NMR for titration of **7** into a mixture of **2** and **15** in* $CDCl_3$ *at 298K. Concentrations are: a) **2**: 0.19 mM; **7**: 0 mM; **15**: 0.25 mM; b) **2**: 0.18 mM; **7**: 0.13 mM; **15**: 0.23 mM; c) **2**: 0.18 mM; **7**: 0.17 mM; **15**: 0.22 mM; d) **2**: 0.17 mM; **7**: 0.26 mM; **15**: 0.21 mM; e) **2**: 0.15 mM; **7**: 0.41 mM; **15**: 0.19 mM; f) **2**: 0.13 mM; **7**: 0.63 mM; **15**: 0.17 mM. Integration of selected proton signals indicated that K(**2•15**) = 1.3±0.3 x K(**2•7**).*

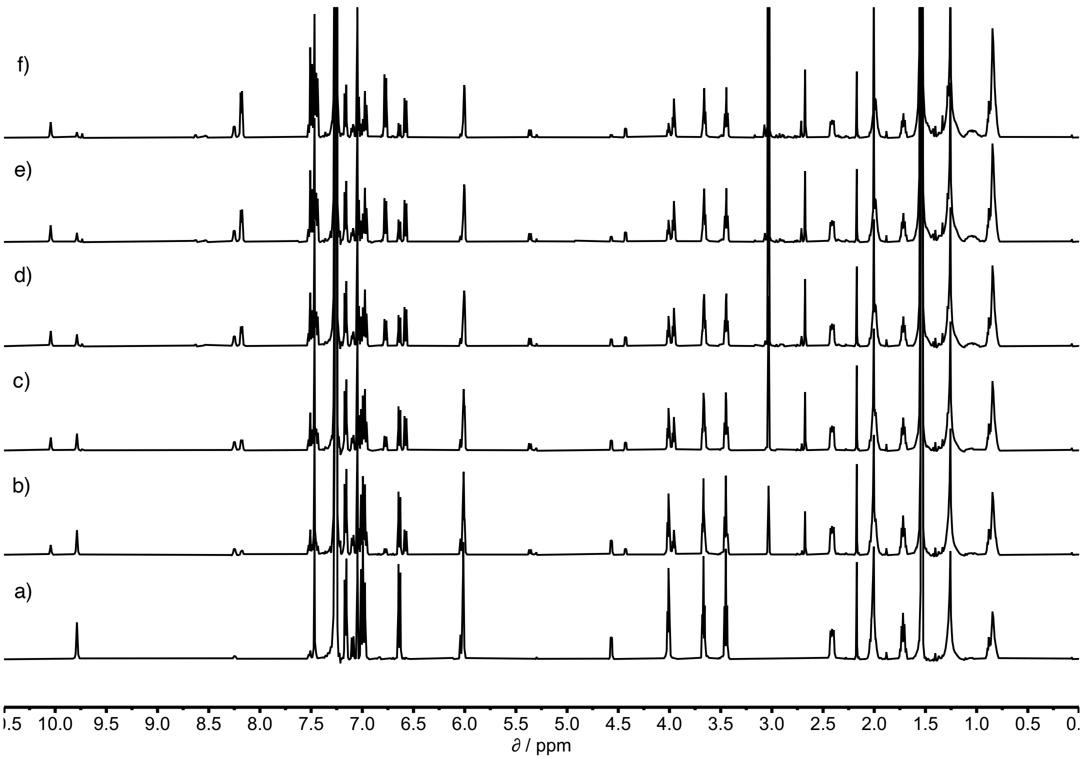

Figure S106. *500MHz ¹H NMR for titration of **7** into a mixture of **2** and **16** in $CDCl_3$ at 298K. Concentrations are: a) **2**: 0.20 mM; **7**: 0 mM; **16**: 0.26 mM; b) **2**: 0.18 mM; **7**: 0.16 mM; **16**: 0.24 mM; c) **2**: 0.17 mM; **7**: 0.29 mM; **16**: 0.22 mM; d) **2**: 0.16 mM; **7**: 0.44 mM; **16**: 0.20 mM; e) **2**: 0.14 mM; **7**: 0.60 mM; **16**: 0.18 mM; f) **2**: 0.12 mM; **7**: 0.78 mM; **16**: 0.16 mM. Integration of selected proton signals indicated that K(**2•16**) = 2.3±0.5 x K(**2•7**).*

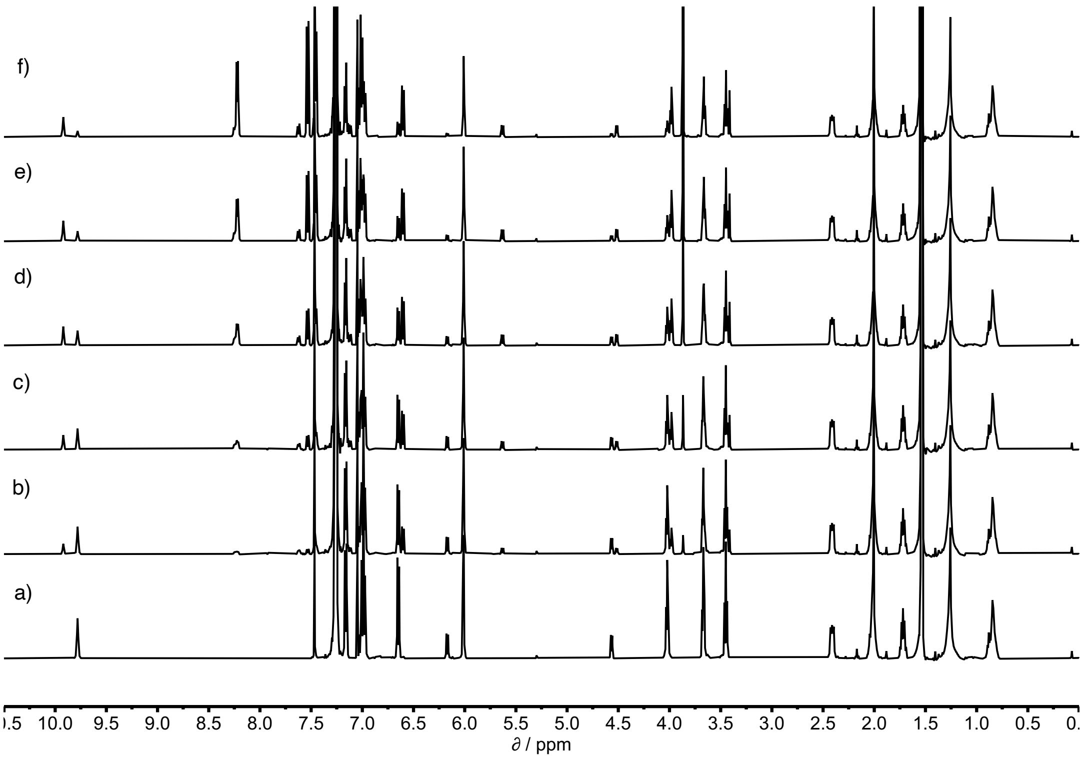

Figure S107. *500MHz ¹H NMR for titration of **10** into a mixture of **2** and **17** in $CDCl_3$ at 298K. Concentrations are: a) **2**: 0.20 mM; **10**: 0 mM; **17**: 0.25 mM; b) **2**: 0.19 mM; **10**: 0.10 mM; **17**: 0.24 mM; c) **2**: 0.18 mM; **10**: 0.20 mM; **17**: 0.23 mM; d) **2**: 0.17 mM; **10**: 0.36 mM; **17**: 0.22 mM; e) **2**: 0.15 mM; **10**: 0.57 mM; **17**: 0.20 mM; f) **2**: 0.13 mM; **10**: 0.84 mM; **17**: 0.17 mM. Integration of selected proton signals indicated that K(**2•17**) = 1.5±0.2 x K(**2•10**).*

4.3. Tetrachloro-aryl-extended calix[4]pyrrole 4

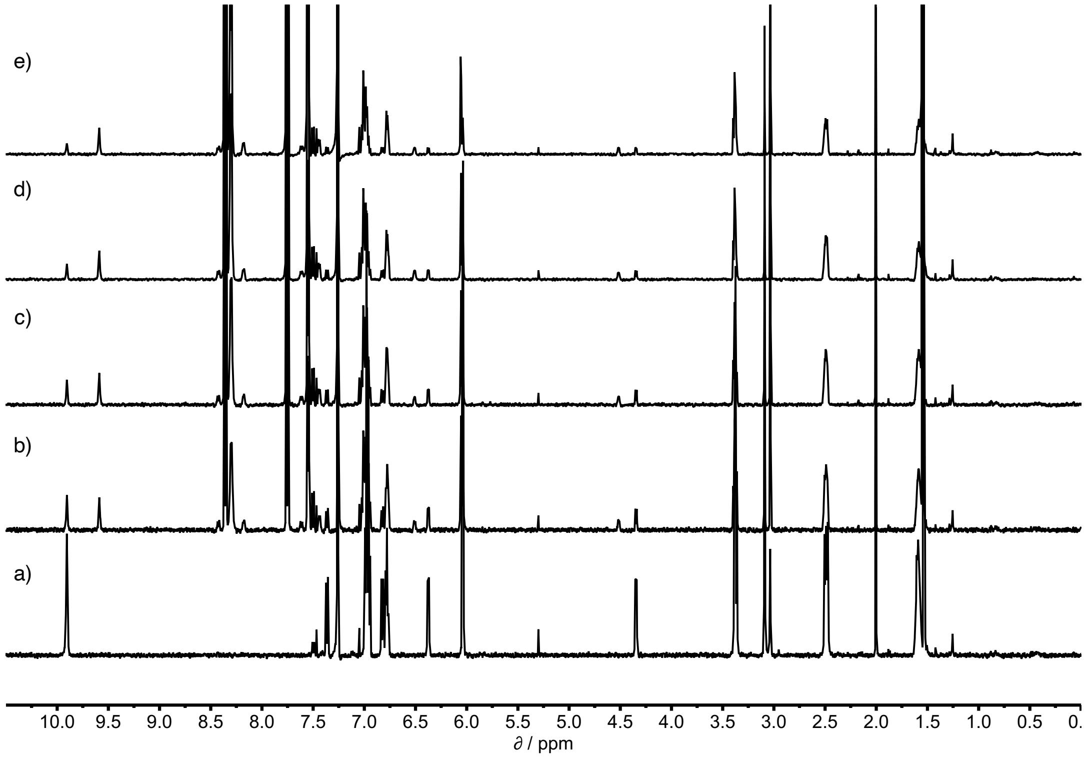

Figure S108. *500MHz ^{1}H NMR for titration of **9** into a mixture of **4** and **7** in $CDCl_3$ at 298K. Concentrations are: a) **4**: 0.79 mM; **7**: 0.93 mM; **9**: 0 mM; b) **4**: 0.45 mM; **7**: 0.53 mM; **9**: 2.12mM; c) **4**: 0.37 mM; **7**: 0.44 mM; **9**: 2.62mM; d) **4**: 0.29 mM; **7**: 0.34 mM; **9**: 3.15mM; e) **4**: 0.23 mM; **7**: 0.28 mM; **9**: 3.48 mM. Integration of selected proton signals indicated that K(**4•7**) = 6.3 ± 0.7 x K(**4•9***
